# Treatment of Posttraumatic Stress Disorder: A State-of-the-art Review

**DOI:** 10.2174/1570159X21666230428091433

**Published:** 2024-01-04

**Authors:** Lisa Burback, Suzette Brémault-Phillips, Mirjam J. Nijdam, Alexander McFarlane, Eric Vermetten

**Affiliations:** 1 Department of Psychiatry, University of Alberta, Edmonton, Canada;; 2 Department of Occupational Therapy, University of Alberta, Edmonton, Canada;; 3 ARQ National Psychotrauma Center, Diemen, The Netherlands;; 4 Department of Psychiatry, Amsterdam University Medical Centers, Amsterdam, The Netherlands;; 5 School of Medicine, The University of Adelaide, Adelaide, Australia;; 6 Department of Psychiatry, Leiden University Medical Center, Leiden, The Netherlands;; 7 Department of Psychiatry, New York University Grossman School of Medicine, New York, USA

**Keywords:** Posttraumatic stress disorder, moral injury, psychotherapy, intervention, psychotropic drugs, ketamine, psychedelic, neuromodulation

## Abstract

This narrative state-of-the-art review paper describes the progress in the understanding and treatment of Posttraumatic Stress Disorder (PTSD). Over the last four decades, the scientific landscape has matured, with many interdisciplinary contributions to understanding its diagnosis, etiology, and epidemiology. Advances in genetics, neurobiology, stress pathophysiology, and brain imaging have made it apparent that chronic PTSD is a systemic disorder with high allostatic load. The current state of PTSD treatment includes a wide variety of pharmacological and psychotherapeutic approaches, of which many are evidence-based. However, the myriad challenges inherent in the disorder, such as individual and systemic barriers to good treatment outcome, comorbidity, emotional dysregulation, suicidality, dissociation, substance use, and trauma-related guilt and shame, often render treatment response suboptimal. These challenges are discussed as drivers for emerging novel treatment approaches, including early interventions in the Golden Hours, pharmacological and psychotherapeutic interventions, medication augmentation interventions, the use of psychedelics, as well as interventions targeting the brain and nervous system. All of this aims to improve symptom relief and clinical outcomes. Finally, a phase orientation to treatment is recognized as a tool to strategize treatment of the disorder, and position interventions in step with the progression of the pathophysiology. Revisions to guidelines and systems of care will be needed to incorporate innovative treatments as evidence emerges and they become mainstream. This generation is well-positioned to address the devastating and often chronic disabling impact of traumatic stress events through holistic, cutting-edge clinical efforts and interdisciplinary research.

## 1 INTRODUCTION

### 1.1 Overview

Posttraumatic Stress Disorder (PTSD) was included in DSM III in 1980 and acknowledged the specific psychopathological impact of extreme life events that include confrontation with death, severe threat, or sexual assault, in contrast to other life stresses [1]. Over 40 years of research has demonstrated the pervasive impact of trauma, including evidence to suggest that PTSD is a systemic illness with neurobiological underpinnings that lead to a range of physical comorbidities [2]. The history of PTSD highlights the important role of cultural and political context, with groups such as Vietnam veterans, the women’s movement, and victims of crime focusing attention on the previous inadequate acknowledgement of the psychological suffering of such groups [3]. As epidemiological research has determined that traumatic events are far more common than originally anticipated, a repeated challenge in the revisions of the DSM has been the boundary of the definition of the stressor criterion, and the categorical nature of the diagnosis.

The long-term costs of PTSD remain substantial at a societal level, despite gains with evidence-based treatment, and has been argued to be similar to Major Depressive Disorder (MDD) in terms of the burden of disease [4]. Improving suboptimal clinical outcomes, particularly in groups such as veterans [5] and the survivors of child abuse, is therefore crucial and will remain so, as we face the consequences of the COVID-19 pandemic and the war in Ukraine [6]. In this context, this state-of-the-art review will first summarise current understandings of PTSD and PTSD treatments before exploring sources of treatment challenges, in part arising from the limitations of clinical and research conceptualizations. Emerging approaches and potential innovations to address these challenges will then be outlined, followed by the proposition of a future framework to catalyze progress.

This narrative review used a semi-structured approach. After creating a preliminary outline based on previous review articles, a MEDLINE search of English articles related to Posttraumatic stress disorders, limited to human clinical studies, guidelines, systematic reviews, and meta-analyses from 2012 to 2022, was conducted on June 22, 2022. The resultant 3700 citations were exported into Covidence software (Veritas Health Innovation) to facilitate screening of abstracts for relevant articles, which occurred until no new material arose. This was supplemented with landmark articles suggested by co-authors from their areas of expertise, and further targeted, iterative literature searches in MEDLINE (Ovid platform), EMBASE (OVID platform), APA PsycINFO (OVID platform), Google Scholar, and Clinicaltrials.gov, as necessary, to ensure broad capture of recent developments.

This body of knowledge will be presented in the context of a discussion of how the dominant conceptualisation frameworks of PTSD can minimise the importance of emerging findings that challenge these paradigms. One critical issue that will be highlighted is the importance of the field developing a systematic approach to treatment non-response. This starts with the diagnosis of PTSD itself, and related knowledge about etiological factors and epidemiology, which will be reviewed in the next sections.

### 1.2 Diagnosis

An ongoing debate within the PTSD field involves the boundaries of the diagnosis itself. This relates to how to classify subsyndromal PTSD and the separation of those with significant symptoms from full PTSD. The second boundary debate is about the specific stressors that can lead to the disorder, which plays a key gatekeeper role for the application of the diagnosis, for example excluding bullying [7]. PTSD is a heterogeneous disorder, and trauma is a risk factor for a variety of psychiatric illnesses ranging from anxiety, mood, personality, and psychotic disorders [8, 9], for which there is considerable overlap and comorbidity. PTSD is typically associated with high rates of comorbid MDD, anxiety disorders, and substance use disorders (SUDs) [10, 11]. For much of PTSD’s history, there have been calls for recognition of a spectrum of trauma-related conditions, which would include not only trauma and stressor-related disorders, mood disorders, and anxiety disorders, but also traumatic grief, somatization, dissociative disorders, and personality disorders [12]. Current PTSD diagnostic categories, including the newer categories of Complex PTSD, will be described below, including critiques relevant for PTSD research and treatment.

#### 1.2.1 Classic PTSD

The two major current diagnostic definitions include the Diagnostic and Statistical Manual of Mental Disorders 5^th^ Edition (DSM-5) and the International Classification of Diseases 11^th^ Revision (ICD-11) PTSD diagnoses, both of which require symptoms to develop following a traumatic experience. DSM-5 PTSD requires at least one month of symptoms following exposure to an actual or witnessed major traumatic event in which there was actual or threatened death, serious injury, or sexual violence (*i.e.* PTSD “Criterion A”) [13]. This may also include developmentally inappropriate sexual experiences without threatened or actual violence. Four symptom clusters must be represented: a) intrusion symptoms (recurrent, involuntary, intrusive distressing memories or dreams, dissociative reactions, such as flashbacks, or distressing psychological or physiological reactions upon exposure to traumatic reminders), b) avoidance of internal or external traumatic reminders, c) negative alterations in mood and cognition (amnesia for an aspect of the trauma, exaggerated negative beliefs, persistent negative emotional states, diminished interest in activities, feelings of estrangement or detachment from others, or inability to feel positive emotions), and d) altered arousal (irritability, angry outbursts, reckless or self-destructive behavior, hypervigilance, exaggerated startle response, or disturbed concentration or sleep). The ICD-11 PTSD diagnosis is similar to the DSM-5, but more narrow, focusing on traditional fear circuitry symptoms such as re-experiencing, avoidance, and hypervigilance [14]. In contrast with the ICD-11, DSM-5 criteria also include a broader range of persistent emotional responses such as guilt, shame, and inability to experience positive emotions, “alterations” in arousal and reactivity, and the addition of self-destructive and risk-taking behaviors.

Criticism of the diagnostic criteria are multiple. First, the DSM-5 criteria have been viewed as too broad relative to the DSM-IV version, making it difficult to compare and interpret results from studies with DSM-IV *vs.* DSM-5 inclusion criteria [7]. However, Heeke *et al.* noted good agreement for PTSD diagnosis between the DSM-5 and ICD-11 criteria in a group of traumatised refugees in Germany, with only 9% meeting criteria under only one diagnostic system, and noting overlap with anxiety and depression within both systems [15]. Second, the binary, cross-sectional nature of the diagnosis is problematic. PTSD has many faces and potential subtypes based on differences in symptoms, treatment response, and neurobiological profiles, which are not captured in diagnostic criteria. Criteria also fail to discern severity and duration of illness or distinguish between the illness and its consequences, which may be important for treatment response [16]. As the field was shaped by exposure-based animal models of PTSD and cognitive models of psychotherapy, fear-based memory and cognitive elements of PTSD became prominent in diagnostic criteria and have led research inquiry and clinical guidelines.

Physical symptoms were seen as a core component of PTSD in its original formulation, a *physioneurosis*, but are now only included as “physiological reactions to internal or external (traumatic) cues”. What the current criteria do not address is the almost ubiquitous presence of physical symptoms such as headaches, gastrointestinal distress and fatigue, which are frequently the symptoms that lead people with PTSD to seek treatment [17]. Research indicates that, for many, PTSD starts with nonspecific, subsyndromal symptoms that follow different trajectories, and may cause as much functional impairment as full PTSD [18]. These symptoms are a major risk factor for full PTSD, especially upon exposure to a subsequent stressor [19]. Further, there is little differentiating the psychophysiological reactivity preceding development of PTSD from that experienced once the full criteria are finally met. Finally, it is clear that trauma is necessary but not sufficient for PTSD, as the substantial majority of trauma survivors do not develop the disorder. Unlike in animal models, individual human differences in executive functioning, coping style, and meaning-making, borne of personality, interpersonal, social, cultural, and personal experiences, play a role in both disease and resilience. These facts contradict the notion of an acute illness beginning after a single trauma as the primary causal factor, the arbitrary diagnostic cut-offs, and the tendency to normalise or minimize early posttraumatic distress symptoms as distinct from the disorder [19].

#### 1.2.2 PTSD Dissociative Subtype and Complex PTSD

PTSD dissociative subtype (PTSD-DT) and Complex PTSD (CPTSD), added to the DSM-5 and ICD-11 respectively, are new diagnostic categories recognizing the existence of PTSD subsets with significant complexity. Both are associated with chronic trauma-related dissociative symptoms, childhood trauma and neglect, greater trauma burden, more severe and chronic course, and greater suicidal ideation, anxiety, depression, and borderline personality disorder comorbidity [20-27]. This matches with latent class analyses indicating the existence of a distinct group of individuals with both high PTSD symptoms and high disturbances in self-organisation, as well as a group with high PTSD symptoms without these disturbances [20, 22, 24, 26-28]. It is important to note that throughout this article, the term “dissociation” will refer to trauma-related dissociation, unless otherwise indicated [29]. This distinction is necessary since this term has been used to describe multiple distinct mental processes and states, ranging from ordinary absent-mindedness, hypnotic states, trauma-related dissociation, Dissociative Identity Disorder, medical states such as partial complex seizures, and states induced by medications like ketamine.

PTSD-DT is characterized by PTSD in addition to persistent depersonalization, derealization, and dissociative amnesia [30]. This subtype, consisting of 12 to 44% of PTSD samples [22], was established based on epidemiological, clinical, and neurobiological evidence of a subset of PTSD patients with prominent dissociation and overmodulation, rather than undermodulation, of affect [30]. There remains controversy as to whether a) depersonalization and derealization adequately encapsulate the dissociative symptoms present in this patient population, b) PTSD-DT is separate and can be distinguished from dissociative disorders, personality disorders, and CPTSD, and c) the subtype predicts treatment outcome [22]. Studies have shown that seemingly distinct dissociative symptom categories are highly associated with each other, refuting the notion that specific symptoms (dissociative amnesia, fugue, depersonalization) are wholly separate [31, 32]. However, this new category may allow needed research in this area and help determine if current treatments based on fear and cognitive paradigms can adequately address this population.

CPTSD, in addition to meeting core PTSD criteria, includes additional features. These include severe and persistent emotional dysregulation, such as self-destructive and impulsive behaviours, altered negative cognitions about the self, with prominent shame or guilt related to the traumatic event, and difficulties sustaining relationships. Originating from Judith Herman’s work *Trauma and Recovery* [33], the CPTSD diagnosis was added to the ICD-11 in 2019 on the basis that this group may require different treatment, such as stabilization before trauma-focused work, and to facilitate research in this area [34]. CPTSD is thought to evolve after prolonged or extreme stressors from which escape was difficult or impossible, such as slavery, torture, prolonged domestic violence, or repeated childhood abuse. Literature has shown the broad and enduring consequences of childhood-onset interpersonal trauma, often leading to characterological issues, chronic dissociation and shame, relational problems, suicidality, and vulnerability to subsequent trauma and physical health conditions [35-37]. While not explicitly stated, the broader DSM-5 PTSD criteria accommodates those who would be diagnosed with CPTSD under the ICD, representing a severe form of the same illness. Given the overlap and associated comorbidity, it is not surprising that studies of CPTSD populations have focused on determining diagnostic boundaries and treatment implications [38].

### 1.3 Etiology

The question of what trauma events are significant enough to elicit PTSD continues to be debated. The social discourse about “trauma” has been overgeneralised in modern society, which risks diluting the significance of traumatic stresses as defined in DSM-5. It is important to avoid overdiagnosis and trivialization of the disorder because of its importance in legal and disability compensation settings. However, overly rigid application of the diagnostic cut-offs might lead to withholding treatment from those who might not fully meet criteria but have essentially the same illness. The progressive revisions of DSM have reflected the increasing understanding of the complexity of the types of trauma exposure that lead to PTSD, such as vicarious traumatization or witnessing threatening events happening to close contacts, family, or friends. While initially included in the DSM-III as an anxiety disorder, subsequent research prompted a move to include it within a new “Trauma and Stressor-related Disorders” section, related to but distinct from surrounding chapters [13].

Further, the risk of developing PTSD varies between classes of traumatic events, suggesting factors beyond fear neurocircuitry in the etiology of PTSD. Interpersonal violence, especially sexual assault, portends a greater risk of developing PTSD than natural disasters [11, 39-42]. Cumulative trauma is an important issue, as risk of PTSD increases with repeated exposure, through sensitization [43]. Prolonged trauma is higher risk than brief experiences [44], as are traumas with subjective responses characterised by acute dissociation [45] or catastrophic cognitive appraisals [46]. Trauma following a Traumatic Brain Injury (TBI) is especially complicated and is associated with over twice the risk of PTSD relative to trauma-exposed groups without head injury, with greater impact in military groups compared to civilians (4.18 *vs.* 1.26 times greater), where TBI is more prevalent [47]. For men, traumas associated with PTSD are more often combat-related or due to physical violence, while PTSD in women is more commonly associated with rape or sexual molestation [11]. The following sections touch on specific etiologic factors important to PTSD and its heterogeneity, such as childhood trauma, interpersonal trauma, and trauma related to occupational, military or disaster exposure [11].

#### 1.3.1 Childhood Trauma

Since the Adverse Childhood Experiences Study (ACES), there is clear evidence that early childhood adversity is a risk factor for psychological and medical disorders later in life [48], including PTSD, likely mediated through epigenetic changes intertwined with psychological and brain development [49], and through intergenerational factors [50]. Four childhood adversities (physical and sexual abuse, neglect, and parental psychopathology) are each associated with a 80% increase in the odds of developing PTSD [51]. Childhood traumatic stress does not occur in a vacuum, however, but is usually accompanied by multiple types of adverse experiences [35, 52, 53], with individuals often having limited social support to buffer the impact [54]. Hughes *et al.* (2005) surveyed 1699 children receiving trauma-focused treatment and reported 78% were exposed to multiple and/or prolonged interpersonal traumas. While less than half met PTSD criteria, most exhibited other posttraumatic symptoms, with half exhibiting emotional dysregulation, inattention, poor concentration, negative self-image, poor impulse control, aggression, and risk-taking behaviors [48, 54]. This can set the stage for later, often atypical or complex forms of PTSD and other psychopathology in adulthood [25, 37, 55, 56], which is often less treatment responsive [56].

#### 1.3.2 Interpersonal Trauma

Interpersonal trauma, now recognized as common [57] and resulting in a high likelihood of PTSD relative to other traumas [11, 39-41], is distinct in that it involves intentional harm. Physical and sexual assault are known to predict PTSD severity and chronicity [39, 58] and poor response to treatment [59]. This may be due to the compounding effects of comorbid childhood trauma or the greater role of shame, guilt, anger, disgust, and trauma-specific rumination [60-64], contributing to lack of trust, emotional numbing, and chronicity of the disorder [61, 65]. Interpersonal trauma can both lead to and exacerbate relational problems, which may promote PTSD chronicity [66]. Childhood maltreatment increases the risk of both insecure attachment and emotional dysregulation, and subsequent relational dysfunction, which may increase the risk of betrayal trauma as an adult [67]. The intersections between childhood trauma, CPTSD, PTSD-DT, and common comorbidities such as MDD, substance use, and Borderline Personality Disorder (BPD) are complex and overlapping [55, 62, 68-72]. The diagnostic, research, and treatment implications are myriad, especially given the impact on trust and therapeutic relationships [73]. Betrayal trauma, perpetrated by a close and often trusted other, has an especially pernicious impact on physical health and psychopathology, including PTSD, dissociation, and shame [71, 74-76], which may be partly mediated by emotion regulation difficulties [77]. Military sexual trauma (MST) resulting from sexual misconduct at the hand of a military colleague, akin to a family member, has more recently been described as a profound from of betrayal [78]. Among Canadian Armed Forces actively serving Regular Force members, 70%, in 2018, reported having witnessed or experienced at least one form of sexual misconduct during the previous 12 months of military service, with 15.4% of respondents (women 28.1%, men 13%) stating they had been personally targeted [78]. This includes inappropriate verbal or non-verbal communication, sexually explicit materials, physical contact or suggested sexual relations, or discrimination on the basis of sex, sexual orientation, or gender identity [79]. Sexual misconduct has been associated with adverse health outcomes, such as increased rates of depression, substance use, sexual health problems, physical health problems, and PTSD in the U.S. military population [80-82]. Interpersonal trauma can fundamentally fracture a person’s self-perception, impacting daily function and ability to engage in relationships.

#### 1.3.3 Occupational Trauma

Occupational PTSD also varies with trauma exposure and various intrapersonal, interpersonal, and systemic factors. For example, health care professionals, including nurses, physicians, and other medical responders may have elevated PTSD and depression risk, with risk increasing with years of service, older age, previous exposure to violence, history of mental disorders, and non-graduate status [83, 84]. Occupational PTSD is of particular relevance to emergency service personnel who have double the risk of developing PTSD during their working career, compared to the general population; ambulance officers are at greatest risk [85, 86]. This includes exposure to a range of traumatic experiences including disasters, mass casualty events, and pandemics, as well as being at risk of interpersonal violence [83-85, 87]. A significantly greater proportion of emergency service personnel have subsyndromal PTSD and represent a group where early intervention has the potential for substantial benefit. For example, Pietrzak *et al.* (2012) studied police involved in the World Trade Centre collapse and found 5.4% had full-blown PTSD four years later, whereas 15.4% had subsyndromal PTSD [88]. There were significant associations with alcohol abuse and somatic symptoms in both groups. They concluded it was important to have a dimensional perspective of posttraumatic stress disorder “... particularly in professions such as police, as operations definitions and conventional screening cut points may underestimate the psychological burden for this population”.

Cumulative trauma exposure in emergency service populations is also a documented risk factor for increasing the onset of PTSD, and mapping cumulative exposure has utility in managing PTSD risk in emergency service personnel [89]. The cumulative risk to emergency service personnel by repeatedly being exposed to death, in particular, has been identified [90]. A study of United Kingdom police states “... that particular care should be taken to assess long-serving officers who have not been promoted for cumulative levels of trauma exposure and PTSD symptoms, as well as to monitor officers who have felt humiliated or sexually harassed” [91].

The cumulative, far-reaching, and long-lasting mental health impacts of the COVID-19 pandemic on health care professionals are also increasingly recognized [92]. During the pandemic, health care professionals were not only at a higher risk of PTSD, estimated at 17 to 29% prevalence [93-95], and its comorbidities, but also burnout and moral injury (see section 4.6 Moral Injury) [96-98], owing to a combination of cumulative traumatic exposures, high infection risk, and work-related stressors, such as staff shortages, lack of personal protective equipment, and moral dilemmas that demand effective organizational responses [98, 99]. PTSD may also elevate the already higher baseline risk for suicide in some health care workers, especially physicians and nurses who possess knowledge and means to complete suicide [92, 100]. The above section clearly highlights the need for monitoring and intervention to manage the specific and unusual risks associated with these particular forms of employment.

#### 1.3.4 Military-related Trauma

The impact of cumulative traumatic stress exposure among military veterans has also been clearly demonstrated. Studies with veterans have found that lifetime trauma exposure is an important predictor of both PTSD and depression in military populations [101, 102], over and above the effects of combat experiences. It is not simply exposure to a single traumatic event, but rather repeated trauma exposures that ultimately results in further sensitisation and neurobiological dysregulation, which eventually leads to the onset of a clinical disorder [103].

Military combat exposure is, however, a particularly strong risk factor for developing PTSD. Length of exposure, seeing others wounded or killed, being a participant in committing atrocities, and peritraumatic dissociation are important risk factors [104, 105]. A dose-response curve has been observed, with a plateau around 25% to 30% developing PTSD, suggesting resilience in the majority [106, 107]. Numerous studies of military personnel describe the aggregated rates of the leading mental health disorders in veterans such as PTSD, TBI, depressive disorders, anxiety, and substance (alcohol or drugs) dependence. Risk factors for adverse mental health outcomes from exposure to combat extend from before military service through to post-military life and thus encompass both armed forces personnel and veterans. The mental health consequences of such exposure may be delayed and first present after military service. PTSD rates are also very high among public safety personnel, including firefighters and military personnel, with one-third to more than one-half exposed to potentially traumatic events [108]. The prevalence of PTSD in combat veterans is estimated to be between 10% and 15%, with lifetime prevalence estimates ranging from 12% to 30% [109]. Demographic factors, job factors, social support, injuries, physical and psychological factors, and individual traits may be important predictors of PTSD in this population [110].

#### 1.3.5 Disaster-related Trauma

Human-made and natural disasters are an important part of trauma research as they can have large scale impact on populations. Disasters test civil administrations’ and health services’ capacity to act in a flexible but well-coordinated manner as each disaster is unique and poses unique challenges. The health services required differ markedly according to the nature of the disaster and the geographical spread of those affected. Increased rates of psychological distress and psychiatric disorders follow natural disasters. Health interventions need to take into account the estimate that at least 20% of the exposed population will be at risk of exacerbation of their pre-existing psychiatric disorders, which are also risk factors for the development of PTSD [19, 111, 112]. There will be a further group who will develop PTSD in the absence of any prior psychiatric symptoms. It is important to anticipate a long period of emergence of the disorder due to the prevalence of delayed onset PTSD. This will necessitate that a health service for the disaster survivors be in place for at least 5 years. However, the effects of the trauma on children can have long-term impacts on separation anxiety, which becomes a traumatic reminder of the disaster for decades [112]. Altered gene expression and intergenerational effects following disaster-related trauma are also of concern and under study [50, 113, 114]. Further, high levels of heterogeneity between studies suggest that disaster variables and post-disaster responses have the potential to mitigate adverse effects [115]. Consistent predictors of adverse outcomes in disaster studies include female gender, socioeconomic disadvantage, high disaster exposure, and low psychosocial resources [116].

#### 1.3.6 COVID-19 Related Trauma

A global catastrophe, COVID-19 and its necessary containment measures led to stark and interacting physical, psychological, medical, economic, social, and cultural sequelae, including elevated PTSD risk [98, 117, 118], which will likely shape society for decades [117, 119-121]. Important risk factors for PTSD during COVID-19 have been identified, including fear of contagion, social discrimination, loneliness, poor health status, poor sleep quality, receiving too much information about COVID-19, financial stresses or instability, depression and anxiety symptoms, history of traumatic events, and psychiatric history [117, 122]. In addition, the unique neuro-pathological characteristics of the virus play an important role.

PTSD and COVID-19 have a complex bidirectional relationship, which is incompletely understood. PTSD development implicates inflammation (see part 2, Progress in Understanding PTSD), and PTSD also alters the immune system, reducing resistance to infection [123]. A history of mental disorder, although not specifically PTSD, has been shown to increase the risk of COVID-19 infection, severity, hospitalization, and mortality [120]. COVID-19 has been shown to activate the HPA axis, and it is associated with insufficient cortisol response [119, 124]. Compared to previous coronaviruses, COVID-19 is more likely to invade the central nervous system and disrupt the blood-brain barrier, causing brain inflammation and entry of stress response molecules that can dysfunctionally activate the medial prefrontal cortex (mPFC), hippocampus, and amygdala. These factors may set the stage for acute, late, and persistent neuropsychiatric symptoms [117, 120, 121, 124]. Mitochondrial dysfunction and subsequent lactate accumulation in the brain, which can trigger panic attacks and flashbacks in PTSD patients, may also play a role (see Sfera * et al.,* 2021 [124] for a review of COVID-19 molecular effects on mitochondria, blood-brain barrier, stress-related disorders and brain-derived neurotrophic factor (BDNF)). Acutely, delirium, insomnia, and psychosis may occur, which are also linked to development of PTSD [125-127], and persistent fatigue, pain, and cognitive dysfunction may impede recovery [117, 120]. Acute damage resulting from brain inflammation may also be exacerbated by certain ICU practices, made worse by isolation policies and mechanical ventilation (see Sankar * et al.,* 2022 [119] for a review).

COVID-19 also “primes” brain microglia responsible for regulating immune responses to pathogens, tissue damage, and stress, adding to priming effects from premorbid influences such as exposure to adversity or deprivation during development, traumatic brain injuries, severe mental illnesses, advanced age, obesity, allergies, autoimmune illnesses, and previous severe infections. Thus, a “reciprocal double vulnerability and sequential triggering” effect has been proposed, wherein previous predisposing proinflammatory conditions create a vulnerability for COVID-19 to trigger a chronic neuropsychiatric condition. In addition, COVID-19 infection also causes an additional vulnerability so that subsequent mild immune, psychological, or traumatic stressors act as future persistent triggers and perpetuators for the COVID-related neuropsychiatric condition (see Tizenberg *et al.* 2021 [120] for a review of this topic). This fits with the allostatic load model of stress-related illness (see section 2.6 Allostatic Load Model of PTSD). Ongoing stressors and uncertainty, and the behavioral consequences of PTSD, such as health, coping and risk-taking behaviors, relationship dysfunction, and avoidance may also influence the onset and course of both illnesses [98, 120, 123]. Like the need to adapt health services for the pandemic [121, 123], including increased use of telemedicine, there is now a need to monitor for and intervene to minimize the long-term neuropsychiatric consequences of COVID-19 [121].

### 1.4 Epidemiology

Far from being “outside the range of usual human experience”, most people will have experienced a major trauma, with threat to life or a loved one, at some point in life [128]. While most will respond with resilience [129], PTSD is highly prevalent. Across epidemiological studies, lifetime prevalence of PTSD ranges from 10% to 20% for women and 6% to 8% for men [11], with variation attributable to distributions of trauma type and severity, economic, cultural, and social factors, and study methodology. A recent systematic review of American studies since 2013 indicated one-year prevalence rates from 2.3% to 9.1% in civilian populations and 6.7% to 50.2% in military samples. Civilian lifetime prevalence ranged from 3.4% to 26.9%, with military lifetime rates from 7.7% to 17.0%, indicating variability depending on samples and methods [130]. Rates of PTSD also considerably vary depending on the type of index trauma and trauma history [11, 128, 130]. Reported PTSD prevalence during the COVID-19 pandemic varied depending on population and time period, ranging from 12 to 27% for the general population, 30% for high-risk groups (*i.e.* pregnant women, and those with cancer, HIV, and other chronic diseases), 17 to 29% for health care workers, and 6.5% to 61% for those infected with the virus [93-95, 117, 122].

#### 1.4.1 Risk Factors

The risk of PTSD following a traumatic event is estimated at 9%, but can depend on many factors related to genetic predisposition, type and number of traumas, and protective factors such as social context [44, 102, 131, 132]. Risk factors overlap with other psychiatric disorders: female sex (twice as likely to develop PTSD), younger age, low socioeconomic background, prior mental disorder, family history of mental disorders, and childhood trauma [44, 108, 130]. While Acute Stress Disorder indicates higher risk for PTSD, at least half who develop PTSD do not initially have Acute Stress Disorder [133]. Attachment style and personality factors, including neuroticism and introversion, can also indicate vulnerability [103, 134-136]. Conversely, positive expectancies are consistently associated with protection against the development of PTSD, with stronger impact for coping-specific self-efficacy and hope, compared with general self-efficacy and optimism. Ongoing stressors and lack of social support after trauma are also important, which may be impacted by stigma [137] and genetic traits [132].

#### 1.4.2 Course

PTSD may present acutely after a traumatic event, have a waxing and waning course, or present acutely after a delayed onset of months or years. Delayed onset, which is more common in military populations, is a diagnosable DSM-5 specifier “with delayed expression” and diagnosed if the illness onset is at least 6 months after the traumatic event [13]. Recent studies indicate that the vast majority of those with delayed onset may have the progressive accumulation of PTSD symptoms during the first year following the traumatic incident [138], but there can also be a significantly longer delay in the emergence of symptoms [139, 140]. In a large sample of Dutch veterans, there was a long-term symptom increase 5 years after deployment [140]. PTSD symptoms are dynamic, and subgroups include a resilient class with few PTSD symptoms, a group with recovery epitomised by gradual remission, a typical delayed onset group, and a group with chronic, consistently high PTSD symptoms [11, 139, 141-143]. A recent metaanalysis of 78 studies on PTSD course in the year following trauma found 27% of individuals presented with PTSD by one month post-trauma, which declined to 18% at 3 months, with minimal reductions thereafter [108]. However, relatively short-term studies like these do not take into account the proportion of cases with delayed onset decades after the index trauma. A metaanalysis of PTSD in community settings [144] found that approximately 56% of individuals with PTSD have a chronic outcome despite receiving treatment, with persistent disability compromising function well into later life. However, the exact proportion of this disability due to inadequate or poorly matched treatment, especially late in the illness, is unknown [19, 145].

#### 1.4.3 Societal Impact

In addition to personal suffering, PTSD costs society in terms of physical and mental health consequences, and the burden of chronic disability. In 2018, the total excess economic burden of PTSD in the US was estimated at $232.2 billion ($19,630 per individual with PTSD), which was driven by direct healthcare costs, unemployment, and disability [146]. PTSD, as a stress-related disorder, is associated with immune dysregulation and many physical health problems, including cardiovascular and cerebrovascular disease, sleep disorders, chronic pain, irritable bowel syndrome, and dementia [8, 19, 147-153]. PTSD is an important source of physical disability and potential mortality, although this may be confounded by the impact of comorbid major depression, especially following critical illness [154]. PTSD is highly associated with cardiovascular disease [153], and risk factors such as chronic hyperarousal, smoking, metabolic syndrome, hyperglycemia, and obesity [2, 155]. PTSD is linked with greater risk of subsequent myocardial infarction and coronary heart disease, even after adjustment for depression [156-158], and higher risk of all-cause mortality in heart failure patients [159]. There is also greater risk of autoimmune disease, cancer, premature mortality, and indices of aging [2, 160, 161]. Taken together, PTSD can be viewed as a systemic disorder, which is not addressed in clinical guidelines. The total disease burden from PTSD, encompassing physical, mental health, and economic consequences is immense, given that PTSD is prevalent cross-nationally, with half of all global cases being persistent [128]. Only half of those with severe PTSD report receiving any treatment and only a minority receive specialty mental health care. Striking disparities in PTSD treatment exist by country income level. Increasing access to effective treatment, especially in low- and middle-income countries remains critical for reducing the population burden of PTSD [128].

## 2 PROGRESS IN UNDERSTANDING PTSD

While conventional models of PTSD have focused on aberrant fear neurocircuitry, stress sensitization, and altered neurohormonal responses, recent investigations have highlighted the complex interplay between genetics and early life experiences, contextual aspects of trauma, and maintaining and exacerbating factors. Investigations of brain circuits and connectivity, including the impact of the disorder on learning, emotion regulation, and executive function, are shedding light on how the illness influences multiple aspects of brain function and behavior. Findings in neurobiology, neuroimaging, endocrinology, immunology, and stress physiology demonstrate that PTSD is a complex and heterogeneous systemic disorder. In the following sections, aspects of PTSD biology will be reviewed, including genetic factors, stress and the hypothalamic-pituitary-adrenal (HPA) axis, fear circuitry and its impact on memory, brain imaging studies, brain connectivity and circuits, and the allostatic load model of PTSD.

### 2.1 Genetic Factors

Genetic and epigenetic changes may predispose individuals to PTSD. Thirty years of genetic research suggest polygenetic factors may account for the vulnerability to develop PTSD; estimates of heritability range from less than 20% to 70%, for female samples [49, 132]. Genes associated with PTSD overlap considerably with those associated with other common psychiatric disorders, such as MDD, alcohol use disorder, bipolar disorder, and schizophrenia [132, 162, 163], including genes involved in the HPA axis, noradrenergic, dopaminergic, and serotonergic systems, and neurotrophins [164, 165] such as BDNF.

Genetic vulnerability to PTSD is highly moderated by early life stress, childhood trauma, and other environmental factors, which regulate gene expression [165, 166]. Epigenetic studies of gene expression following trauma have implicated DNA methylation and gene expression changes in the HPA axis, including glucocorticoid receptors (GRs), GR response elements [167], and inflammatory genes [168, 169]. Few studies in this area have examined resilience patterns. However, there is some evidence that low resilience is associated with gene expression patterns in immune and dopamine genes, and high resilience with a blunted inflammatory response [169]. Child abuse may occur during critical developmental periods during which the limbic system and HPA axis are developing, along with neural circuits important for emotional regulation, highly influenced by the quality of the child’s attachments. Therefore, both nature and nurture are interwoven in the process. PTSD genetics research has expanded to include study of gene transcription products and interactions between gene products and other cell molecules, an important step to find biomarkers of the illness and of resilience following trauma, and to differentiate those related to the development of PTSD from MDD [49, 170].

### 2.2 Stress, the Hypothalamic-pituitary-adrenal Axis, and Inflammation

Hormonal and immunological impacts of PTSD are associated with a range of physical consequences [171]. Classically, based on the early [172] and later [50] work of Yehuda, PTSD is associated with paradoxically low basal cortisol and high catecholamine levels, although the relationship between the HPA axis and glucocorticoids is nuanced and may be influenced by early life stress, epigenetic influences, and comorbid depression [173, 174]. For a detailed review of the pathways between cortisol-related regulation of genes and PTSD therapy, see Castro-Vale and Carvalho (2020) [175]. Genetic variants of the GR and its binding protein, which regulates the amount of glucocorticoids available to the GR, may lead to reduced glucocorticoid signalling. Lower cortisol levels may fail to bind enough to GRs to ensure stress homeostasis, leading to chronically elevated catecholamines, contributing to trauma memory over-consolidation and overgeneralization, which will be described in Section 2.3 However, other research indicates that although there is evidence of HPA axis and cortisol dysregulation, relationships are complex. There may be a tendency for “hyperregulation” of the cortisol system, leading to either hyper or hypocortisolemia, depending on factors such as moment to moment stressors, level of emotional experiencing, expression and sensitivity of the GR, and comorbidities such as MDD [1]. Sex hormones may also be implicated as females have a greater noradrenergic response, amygdala activation and startle response to highly arousing negative emotional stimuli [176-179]. The menstrual cycle impacts PTSD phenomena, including greater flashbacks and poorer extinction retention in the mid-luteal phase for women with PTSD [180, 181]. This may be related to reduction in the conversion of progesterone to GABAergic metabolites allopregnanolone and pregnanolone, and influences on GRs [182]. The result of this HPA axis dysfunction is chronic hyperarousal, an important predictor for PTSD chronicity [183], memory and attention deficits [184, 185], and anger, which may be associated with catastrophic cognitive appraisals that further exacerbate perception of threat and avoidance behaviors that maintain PTSD [186].

As numerous studies have demonstrated, chronic stress and inflammation have many other consequences, including reduced dopaminergic function [187] leading to dysphoria and anhedonia, and serotonergic dysfunction, which may be related to impulsivity, suicidality, and aggression [188]. PTSD is overall associated with higher levels of proinflammatory cytokines and inflammatory mediators, such as interferon gamma (IFNɣ), tumor necrosis factor alpha (TNF-α), C-reactive protein, white blood cells, and interleukin (IL)-1beta (IL-1β) [189, 190]. It has been suggested that the impact of MDD must be disentangled from PTSD, however, since both are associated with increased proinflammatory cytokines, decreased neurogenesis, mitochondrial and HPA axis dysfunction, and oxidative stress [171, 187]. Depressive episodes can also lead to the sensitization of immune-inflammatory pathways [191]. However, both PTSD and MDD are arguably part of the same spectrum of physiologic and immune dysregulation occurring after trauma and chronic stress. Endocannabinoids are also associated with regulation of glucocorticoid and stress responses, and cannabinoid type 1 (CB1) receptors mediate glucocorticoid action to consolidate aversive memories [192-194]. Other neurosteroids, such as allopregnanolone may also be involved in reducing noradrenaline and glucocorticoid signalling [195]. These will be discussed in relevant sections of Part 5 of this review.

### 2.3 Fear Circuitry and Memory

In addition to abnormal stress responses, aberrant fear conditioning and fear extinction, and their impact on traumatic memory, are among the most researched PTSD paradigms. At the time of a threatening experience, sensory input is compared to previous experience and subsequently activates two parallel threat systems: 1) a subcortical, sensorimotor response and 2) cortical awareness and cognitive appraisal [196]. These two processes are associated with psychophysiological, emotional, and behavioural responses which are often a mixture of automatic defensive responses (such as orienting, fight, flight, and freeze) and those modified by top-down processing (for example, overriding a flight response and remaining still if fleeing would be harmful, as in the case of a mugger threatening to shoot if the victim moves) [197, 198]. Trauma overwhelms these natural threat responses, leading to alterations in executive function, limbic activation, and both explicit and implicit memory systems; hence, the traditional frontolimbic model implicates the hippocampus, amygdala, and prefrontal cortex. The hippocampus, in part, mediates emotional responses to the context of a stressor and integrates elements of recalled declarative memory into a coherent whole, including space and time. It is therefore thought that the hippocampus may be involved in the disorganised, fragmented, and often sensory nature of traumatic memories [1]. The hippocampus is stress-sensitive and structural magnetic resonance imaging studies have shown smaller hippocampal volume in patients with PTSD [137, 199], leading some to view PTSD as a hippocampal-based disorder. However, most of these studies were cross-sectional and did not address whether the smaller volume was secondary to stress-induced damage or pre-existing factors, such as childhood adversity [200].

Fear-associated release of stress hormones promotes limbic-mediated associative learning between cues present at the time of trauma and fear responses. Reactivation of these memories, often by minor reminders, causes a re-experiencing of the sensory and emotional components of the experience, along with psychophysiological arousal. Because of the poor integration into autobiographical memory systems, it can feel like it is happening again in the present. The resulting negative emotion may also further increase attentional bias towards threat [201]. This can cause a vicious cycle of fear and arousal, which strengthens trauma memories and the associative learning to trauma cues and leads to a chronic stress response, with increasing allostatic load. In addition, freeze responses, thought to involve altered states of consciousness and opioid release as a response to imminent, inescapable threat, may worsen or further alter memory recall, or worsen subjective experiences of powerlessness or incapacity [197]. All these contribute to executive dysfunction and emotion dysregulation.

However, while important, the fear circuitry model misses the fact that some traumatic experiences causing PTSD do not primarily involve fear, but rather a cascade of emotions including horror, disgust, and revulsion, such as witnessing mutilated and decomposing bodies. Many events that induce fear can also include these emotional aspects, as well as anger, guilt, and shame. There has been little research into these types of events and how they might be differentiated from events that involve intense fear. Like fear, these “moral emotions” are associated with activations of the amygdala, prefrontal cortex (PFC), and insula [202], and impact sense of the self, implying a role for the precuneus, which will be discussed below. Knowledge about these emotions and the amplifying effect of cumulative stress through sensitization [203, 204] remains to be fully understood. Animal and preclinical research findings may, therefore, not translate to clinical care.

Memory research in the last 25 years, however, is leading to a better understanding of how memory reconsolidation is impacted by context, with bearing on therapeutics. Memory reconsolidation theory states that remembering an event can cause the memory trace to transition from a stable to an unstable state. Once destabilized, it can be altered either pharmacologically or through new experiences before it is restabilized through protein synthesis dependent memory reconsolidation processes [205]. This allows an organism to update long-term memories as needed based on subsequent experiences. Recall alone is not enough to alter old memories; new information must be present at the time of recall for this labilization to occur. Recent data suggests there must be a mismatch (prediction error) between expectation and what actually happens at the time a traumatic memory is recalled [206, 207]. This may have bearing on timing of pharmacological interventions, and how specific psychotherapeutic interventions are carried out. Reconsolidation processes are complex, with contributions from the N-methyl-D-aspartate (NMDA) glutamate receptor (NMDAR), metabotropic glutamate receptor (mGluR), beta-adrenergic receptor, mitogen-activated protein kinase kinase (activated by NMDAR), mammalian target of rapamycin (activated by mGluR), GR, g-aminobutyric acid (GABA) receptor, cannabinoid receptor type 1 (CB1), and serotonin receptors, with downstream impacts on protein synthesis required for synaptic remodelling. For a review, see Raut *et al.* [205]. These will be further explored in Section 5.3 Emerging Pharmacological Treatments.

### 2.4 Brain Imaging Studies

PTSD has been lifted by developments in brain imaging studies, from structural to functional imaging, of which functional Magnetic Resonance Imaging (fMRI) and Positron Emission Tomography have yielded many novel insights. Two decades ago, the disorder was mostly viewed as a hippocampal-based disorder, based on structural imaging studies. Due to novel fMRI and Positron Emission Tomography methods, including script-driven paradigms, PTSD may be viewed as a disorder of emotional regulation [1]. It is now recognized that a wide variety of interrelated brain areas are implicated in PTSD, in addition to the traditional frontolimbic model emphasizing areas of the limbic system, including the hippocampus, amygdala and parahippocampal gyrus, and the PFC. Hyperactivation of the amygdala and dorsal anterior cingulate cortex (dACC), hypoactivation of the ventromedial prefrontal cortex (vmPFC), and hippocampal atrophy are as of yet the most robust findings in PTSD [208]. Compared to MDD, which is also associated with reduced hippocampal volume, PTSD is associated with reduced total brain volume [208-210]. Hyperactive insulae and reduced volumes of the insula, mPFC, and anterior cingulate, associated with attention and emotion regulation, are other findings [209, 210]. Fear conditioning and extinction fMRI studies report increased activation in the a) anterior hippocampus (extending to the amygdala) and mPFC during conditioning, b) anterior hippocampus-amygdala regions during extinction learning, and c) anterior hippocampus-amygdala and mPFC areas during extinction recall, as well as decreased activation in the thalamus [211]. Reduced capacity for contextual processing, leading to impaired differentiation between safety and threat, may involve the vmPFC, hippocampus, and thalamus. This suggests that PTSD is characterized by increased activation in areas related to salience and threat, lower activation in the thalamus (a key relay hub between subcortical areas) and vmPFC [211], and failure of inhibition of conditioned fear responses.

The precuneus and insula are important areas for understanding how the brain integrates information, including that related to self-perception and threat. The precuneus is thought to be involved in memory retrieval, creativity, sense of self and associated processes, such as perspective taking, and sense of agency, and consciousness [212-216]. Closely related to the precuneus, the insula is a neural integration hub associated with salience detection, physical and emotional pain, interoception, autonomic regulation, empathy, emotional and self-awareness, and emotional valence [202, 217-219]. The insula integrates information needed to monitor the internal and external environment, playing a role in reinforcement learning, emotional control and decision making [202, 220]. Recently, the insula has been shown, in an animal model, to remember the location and nature of peripheral immune responses and, when reactivated, cause a resurgence of the illness [221], demonstrating connection to the systemic immune system.

Another area of interest involves differential brain activation in PTSD-DT, in contrast to classical PTSD. As stated, in classical PTSD, there is prominent underactivation of the vmPFC and overactivation of the amygdala, insula, and dACC during fear provocation tasks, which corresponds to hyperarousal symptoms. However, patients with PTSD-DT exhibit the opposite during exposure to cues: overactivity of the vmPFC, with decreased amygdala and insula activity consistent with increased inhibition of the limbic region [208, 222]. PTSD-DT is also associated with greater functional connectivity between the amygdala and PFC, and to the parietal areas involved in consciousness, awareness and proprioception, compared to classic PTSD [208, 223]. This supports the idea that classic PTSD involves overactivation and under-regulation of emotional responses, while the opposite may be true for the dissociative subtype, which may explain dissociative and numbing symptoms [222].

Generally, most people with PTSD experience both extremes, to some degree. Models have been proposed to explain such vacillations, including alterations in attentional biases, with reciprocal inhibition between the amygdala and vmPFC, which predict behavior and symptoms [224]. The reciprocal inhibition model predicts that when the amygdala is dominant, patients enter an emotional undermodulatory state, in which they show attentional bias toward threat and manifest re-experiencing symptoms. In contrast, when the vmPFC is dominant, patients are predicted to enter an emotional overmodulatory state, in which they show attentional bias away from threat and avoidance symptoms, associated with decreased amygdala activity. The role of the periaqueductal gray has also been implicated in regulating active and passive threat responses, including dissociation [197]. Given the potential impact on symptom measures, the role of dissociative states must be taken into account in future PTSD treatment studies.

The impact of Adverse Childhood Experiences (ACEs) type, timing, severity, and chronicity to induce specific effects on stress-sensitive brain structures, predisposing to PTSD, has also been an area of focus. Amygdala and hippocampal volume are associated with ACEs severity during a period covering preadolescence and early adolescence, which may be driven by the severity of neglect [225, 226]. PTSD is also associated with disrupted white matter in the corpus callosum [227, 228], a structure connecting the hippocampi bilaterally. This association persists, however, even after accounting for childhood trauma exposure, comorbid depression, history of traumatic brain injury, current alcohol abuse or dependence, and current use of psychotropic medications [227]. An analysis, which took trauma context and age into account, suggested that these white matter changes vary depending on traumatic experience type and are associated with changes in brain circuits related to the emotional and cognitive processing of contexts [229]. A metaanalysis on childhood maltreatment-related PTSD reported differences in the corpus callosum, total cerebral volume, cerebellum, hippocampus, and amygdala, which appeared significantly smaller in maltreatment-related PTSD participants [228]. Altered network centrality of the cingulate, precuneus, and insula has also been found in those with a history of childhood maltreatment [230]. Noted limitations include lack of longitudinal studies, confounding psychiatric comorbidities and maltreatment severity, and inadequate power [228]. These findings, however, indicate that the pathophysiologic development of PTSD may precede the index traumatic event, rather than originate from it.

A further body of research has also recently demonstrated the role of the brain stem in the differential patterns of cortical activation in dissociative and non-dissociative PTSD [231]. Vestibular and periaqueductal grey activation are important drivers of patterns of cortical activity, highlighting the role of brain stem arousal in the engagement of cortical networks [232]. This reflects the changed patterns of internal and external perception and information processing as being core elements of PTSD [233].

### 2.5 Brain Connectivity, Synaptic Plasticity, and Circuits

The newest approach to brain imaging is brain connectivity studies. The frontolimbic model of PTSD has given way to a triple network model based on the Triple Network Model of Menon, which proposed that three main core neurocognitive networks, the Default Mode Network (DMN), Central Executive Network (CEN) and Salience Network (SN), are implicated in a range of psychiatric disorders [208, 234]. Core structures, brain imaging findings, and implications for PTSD within these networks are found in Table **1** [235-240].

Shifting brain states in PTSD may be accounted for by variations in activity within these circuits. PTSD is typically associated with overactivation of the SN and hypoactivation of the DMN and CEN [208, 235]. The SN is involved in stimulus detection, both threat and stimuli associated with homeostatic regulation, interoception, autonomic function, and reward processing, with extensive subcortical and limbic connectivity. It is thought that the SN, *via* the insula, modulates switching between the CEN and DMN, depending on tasks. The anterior insula and dACC are hyperactive in PTSD, implying that the SN is involved in heightened threat detection and autonomic dysfunction in PTSD; impaired SN mediated regulation of the DMN and CEN may also lead to poor regulation of the limbic system. Further, overactivation of the sensory cortex, due to activation of sensory trauma memories, may overwhelm the PFC and further disrupt the CEN [235]. The DMN is implicated in self-referential thoughts and introspection [236]. PTSD patients have been found to have altered self-referential processing, altered DMN structures, and less connectivity between them. DMN alterations in PTSD have been associated with chronic trauma and both hyperarousal and dissociative symptoms [235, 241]. While the amygdala is not normally associated with the DMN state, in hyperarousal and hypervigilance PTSD states, the DMN exhibits altered DMN functional connectivity with the amygdala [235]. Similarly, reduced connectivity in the CEN has been linked with PTSD, and it is thought this may underlie altered working memory and poor emotional control [235]. Impaired emotion regulation, including impulsivity, irritability, emotional dysregulation, and concentration deficits may therefore be a consequence of multiple factors, including intense emotion triggered by memory reactivations, altered attention and executive functions, and activity in areas related to self-referential processing.

This model is likely incomplete, however. While the DMN is often thought to be anticorrelated to dorsal attention networks, this may not be the case, and more subtle relationships may exist that allow balance between reflexive behaviors and constraints of the social and physical environment [236]. Some authors have noted the overlap between networks impacted by PTSD and those of social cognition, which broadly includes a) the DMN, associated with mentalization capacity, empathy, morality and introspection, and b) the mirror neuron system, associated with action identification, encoding of facial expressions and body language, and overlap with attention and frontoparietal control networks [239]. Further, the vmPFC and orbitofrontal cortex are involved in networks conveying sensory and visceromotor information to subcortical areas and may link this information to areas involved in social behavior, mood control, and motivation [236]. Further, it has been proposed that the DMN is intimately tied to the replay of memories during mental simulation, planning, and evaluation, with important implications for memory consolidation, learning, and envisioning and predicting the future [236, 238]. Therefore, understanding of the implications for PTSD will likely continue to evolve.

Importantly, these brain networks are impacted by chronic stress, stress sensitivity, and inflammation, which are associated with excitotoxicity, alterations in glutamate neurotransmission, and NMDA and α-amino-3-hydroxy-5-methyl-4-isoxazolepropionic acid receptors (AMPAR), lower BDNF levels, and synaptic loss in the PFC and hippocampus [242]. Literature suggests the possibility of neuro-progression of PTSD, parallel to clinical deterioration, in a subset of patients, related to neuroinflammation, oxidative stress, and structural brain changes. Neurodegeneration may be particularly associated with a subset of patients whose symptoms worsen or are maintained at a high intensity over time, with progressive change in the frontal lobe (reduced size) and worsening of both neurocognition and physical, psychological, social, and environmental functioning [243]. The following section reviews the related concept of allostatic load.

### 2.6 Allostatic Load Model of PTSD

To better understand the link between exposure to stressors and health outcomes, the model of allostasis has been found helpful. Allostasis, kindling, and sensitisation are constructs that assist in conceptualizing the progressive dysregulation and symptomatic distress following trauma exposure that lead to the onset and course of PTSD [244]. Sensitisation refers to how environmental triggers progressively provoke a greater amplitude of response over time, leading to an enduring increase in response amplitude [245]. Sensitisation can occur in a range of biological systems that underpin PTSD and is manifest as an increasing amplitude of the response to challenge of the physiological system. Kindling is a related construct [245] that has been used to characterise the underlying pathophysiological mechanisms during the emergence of progressive limbic abnormalities in PTSD. Furthermore, sensitisation and kindling are also used to describe a secondary process following the first episode of PTSD and predict the increased risk of subsequent episodes. In essence, the interacting roles of stress sensitisation, fear conditioning, and the failure of extinction in PTSD are central to its onset and maintenance [246, 247].

Allostatic load is a measure of cumulative biological burden; the cumulative cost of repeated stress exposures that challenge the individual’s ability to achieve stability following challenge [248]. This requires the maintenance of biological homeostasis by systems that are activated and then downregulated in the face of extreme stress, which underpins the success or failure of adaptation. In essence, allostatic load is the consequence of repeated cycles of allostasis (*i.e.*, adaptations to threat). The allostatic load model has been used to refocus the stress disease literature, emphasising that their multiple biological systems are vulnerable to a temporal cascade of dysregulation due to exposure to repeated stress and triggers in the environment [204]. In turn, these progressive dysregulations lead to the emergence of a range of possible trajectories of symptom development and chronicity. The essence of the allostatic load model is that the body is subject to wear and tear with repeated activation during stressful situations [249]. It includes the physiological consequences of health-damaging behaviors, such as poor sleep and circadian disruption, lack of exercise, smoking, alcohol consumption, and unhealthy diet. When environmental challenges exceed the individual ability to cope, then allostatic overload ensues [250] as a transition to an extreme state where stress response systems are repeatedly activated and buffering factors are not adequate. These stresses and threats dysregulate homeostasis by initiating activation of multiple neurohormonal, inflammatory, and neural systems.

Several studies have tried to identify allostatic load through biological markers. Some approaches define an allostatic load battery model. For example, Seeman *et al.* identified primary, secondary, and tertiary biomarkers, as well as additional biomarkers in the allostatic load response [251] (Table **2**). There are neuroendocrine and immune systems that respond to internal or external challenges and promote adaptation to threats or adversities: a) the hypothalamic-pituitary-adrenal axis plays a key role in the pathophysiology of allostatic load, b) brain architecture and neurochemical functions are affected by both genomic and nongenomic mechanisms, c) adjustments in the immune system (*e.g.*, leukocytes, cytokines, inflammation) occur, with immunosuppressive effects in the long run, and d) alterations in body functions involving cardiovascular and gastrointestinal systems, endocrine-metabolic balances and sleep may ensue [250].

In summary, allostatic load is the manifest consequence of cumulative exposure to traumatic events on stress systems and their increasing dysregulation [252]. It derives from the definition of allostasis as the ability of the organism to achieve stability through change, and the view that healthy functioning requires continual adjustments of the internal physiological milieu. Hence, one approach to conceptualising the different forms of PTSD and their psychological and physical comorbidities uses the allostatic load model to characterise the impact of stress over time on an individual’s adaptation and dysregulation. The allostatic load model explains the risk of increasing severity of symptoms and a worse outcome from treatment if ongoing exposures occur once PTSD has begun to emerge [253].

## 3 CURRENT STATE OF PTSD TREATMENT

### 3.1 Overview

Treatment for PTSD has, like neurobiological studies, historically focused on remediation of abnormal fear circuitry and its consequences, such as trauma-related cognitions. Exposure-based psychotherapies and medications targeting anxiety and hyperarousal have been the mainstay of PTSD treatment. Multiple treatment guidelines exist globally, and reviews of guidelines can be consulted for a comparison of recommendations across them [254, 255]. Recently, the quality of international treatment guidelines for PTSD was reviewed and differences between guideline recommendations were evaluated. Fourteen guidelines, published between 2004 to 2020, were identified [255]. Recommendations for core PTSD symptoms do not differ greatly between guidelines, which generally consider both psychological and pharmacological therapies as first-line treatments. All but one guideline recommended Cognitive Behavioural Therapy (CBT) as first-line psychological treatment and selective serotonin reuptake inhibitors (SSRIs) as first-line pharmacological treatment. Prazosin is discussed in several guidelines for the treatment of nightmares, but recommendations varied widely. Most PTSD guidelines were deemed to be of good quality; however, many could be considered out of date. Newly updated Australian guidelines now also include recommendations for CPTSD [256]. Medication recommendations for PTSD vary considerably across guidelines, with divergent recommendations for or against pharmacotherapy as first-line therapies in light of recognition of the limitations of medication treatment [254, 255], the greater effect sizes for Trauma Focused Psychotherapies (TFPs), personal preferences, and the lack of access to evidence-based psychotherapy [257].

One of the important debates in the development of treatment guidelines is the question of legitimate control or placebo interventions [258]. There has been debate as to whether treatment guidelines have overstated the effectiveness of certain therapies at the exclusion of others by allowing wait list control studies to be included in meta-analyses that examine the size of treatment effects [259]. Others have argued that the superiority of treatment should only be determined when there are head-to-head trials of active treatments [259]. Such studies have produced surprising findings such as the equivalence of sertraline with enhanced medication management and prolonged exposure when it has been claimed that in veterans psychological therapies are superior [260]. These debates have led to a degree of disaffection in some clinicians who have come to see treatment guidelines as a method of restricting the use of therapies that are equally effective. The following sections review the current state of evidence-based pharmacological and psychotherapeutic treatment of PTSD.

### 3.2 Pharmacological Treatment

There are no specific pharmacologic treatments developed for PTSD and, in the whole of the history of PTSD, there remain only two FDA approved medications for the disorder, sertraline and paroxetine. Both were registered for treatment of PTSD in 2001. Pharmacologic PTSD treatment is, generally, only marginally efficacious [254, 255, 261]. Clinical guidelines generally recognize that, aside from sertraline, paroxetine, fluoxetine, and venlafaxine, few of the currently prescribed PTSD treatments are adequately supported by quality randomised controlled trials [254, 256]. Positive results for SSRIs, initially found helpful for comorbid anxiety and depression, prompted trials of tricyclic antidepressants, monoamine oxidase inhibitors, and other serotonergic medications for PTSD, which are often used as second-line agents due to increased adverse effects and lower quality of evidence.

Prazosin, an alpha1-adrenergic antagonist frequently used in clinical practice, has been an area of discussion and some controversy [254, 255]. Prazosin was found effective in small randomized controlled trials (RCTs), especially for the treatment of nightmares, which are strongly linked to suicidality [262]. However, later results from large RCTs cast doubt and prompted intense scrutiny of available evidence [254, 255, 263]. Recent meta-analyses indicate prazosin is likely effective, especially in those with more severe adrenergic dysfunction, who may have been excluded from negative trials [255, 264]. Furthermore, high placebo response rates in some trials may have led to underestimation of effect. Effect size for overall improved PTSD symptoms scores are small, but medium for nightmares and sleep quality, indicating clinical utility, especially for hyperarousal and nightmares [264].

Second-line medications, supported mostly by open trials or small RCTs, include imipramine and phenelzine, and adjunctive antipsychotic medications, such as risperidone [261], quetiapine, olanzapine, and aripiprazole [265], recommended in treatment-resistant cases or when there are disabling symptoms [254]. Recent evidence indicates quetiapine monotherapy may be useful [261]. Multiple other medications commonly used in clinical practice for PTSD symptoms, including mirtazapine, other antipsychotics, mood stabilizers, buspirone, clonidine, guanfacine, propranolol, and trazodone have a limited evidence base to support them [254, 266]. Trazodone, for example, is prescribed for PTSD-related insomnia and nightmares in up to 21% of veterans and active duty military members in the United States [267, 268]. However, there are no RCTs for trazodone for PTSD in the adult population. Its use is empirical; its evidence base for PTSD includes one veteran survey, case reports, a small open study, and a trial of trazodone as an adjunct to fluoxetine in adolescents for insomnia [269-272]. Benzodiazepines are often prescribed in the context of PTSD to quell anxiety, but they are generally discouraged due to: a) evidence of a 150% increased risk of PTSD development when given in the aftermath of trauma [273], b) lack of efficacy for PTSD and c) increased risk of benzodiazepine abuse. Despite the small effect sizes, limited evidence base, significant relapse after discontinuation, and potential for adverse effects from currently prescribed PTSD pharmacotherapy, it is recognized that many of these treatments can be useful to treat PTSD comorbidities or individual PTSD symptoms, such as depression, anxiety, hyperarousal, and insomnia. Therefore, many of these medications remain in the clinical armamentarium (Table **3**) (See Holder *et al.* [267] and Loeffler *et al.* [268]). There is limited information, however, to inform appropriate combinations of medications and psychotherapy.

### 3.3 Psychotherapeutic Treatment

Trauma-focused psychotherapies (TFPs) are amongst the most evidence-based treatments for PTSD, with larger effect sizes than currently accepted pharmacotherapies and good evidence for long-term impact [274, 275]. However, they are generally underutilized [276, 277]. Psychotherapies are essentially experiential treatments that attempt to induce corrective states of habituation, safety, coping, self-efficacy, compassion, acceptance, and emotional experience. Both trauma- and non-trauma-focused psychotherapies have been utilized for PTSD. In addition to non-specific therapy factors, such as therapeutic alliance and instillation of hope, TFPs focus on desensitization of traumatic memories and amelioration of the consequences of trauma, in keeping with current models of PTSD. Evidence-based TFPs typically include many of the following elements: trauma psychoeducation, establishing a sense of safety, exposure to and desensitisation of trauma-related cues, skills development, cognitive restructuring, and construction of a coherent trauma narrative. Exposure-based and cognitive interventions are based on the idea that promoting emotion regulation while exposing the person to the trauma memory or its narrative will result in extinction learning, changes in trauma-related cognitions, and resolution of avoidance behaviors. Focusing on traumatic memories allows them to enter working memory. They then become labile and vulnerable to modification, allowing new contextual information, including new meanings, to modify the memory during reconsolidation [278] after the therapy session. Non-trauma-focused therapies do not specifically focus on the trauma or traumatic memories but may address their consequences, symptoms, and current life problems. They may focus on coping skills acquisition, problem solving, meaning-making and acceptance, addressing functional problems and relationships, offering support, and reducing shame [279].

Generally, Prolonged Exposure (PE), Cognitive Processing Therapy (CPT), Trauma-focused Cognitive Behavioural Therapy (TF-CBT), and Eye Movement Desensitization and Reprocessing (EMDR) are recommended for adults, and TF-CBT or EMDR for children and adolescents [254, 255, 266, 280-282]. Some guidelines also recommend Narrative Exposure Therapy (NET), group TF-CBT, Brief Eclectic Psychotherapy, and guided internet-based TF-CBT, as well as the following non-trauma-focused therapies: Interpersonal Psychotherapy (IPT), Present Centred Therapy, and Stress Inoculation Training [254-256], with some disagreement amongst guidelines. Table **4** outlines psychotherapies recommended in at least one clinical guideline for PTSD. Trauma-focused therapies are generally more effective for reducing PTSD symptoms than non-trauma-focused therapies [283]. However, non-trauma-focused therapies can also reduce PTSD symptoms, challenging the notion that exposure is always necessary. Efficacy of the four main evidence-based trauma treatments (PE, CPT, TF-CBT, and EMDR) are considered comparable, although there is some debate over whether one may be more effective, efficient, or cost-effective than another [274, 275, 284-287]. Narrative Exposure Therapy (NET) and Brief Eclectic Psychotherapy for PTSD (BEP), both newer treatments, are now supported by multiple studies, with moderate to large effect [280, 288-290]. Generally, therapies aim to be delivered weekly in 8-16 sessions (Table **4**) [291].

### 3.4 Factors Associated with Treatment Outcome

Successful PTSD psychotherapeutic treatment is associated with improved PTSD biological and psychological symptoms. Hyperarousal symptoms tend to change first and predict alterations in both avoidance [292] and markers of sympathetic autonomic dysregulation such as galvanic skin response, resting heart rate, and heart rate reactivity [293, 294]. Functional changes have been reported in the activity of the amygdala, thalamus, caudate nucleus, precuneus, and the ventromedial and dorsolateral prefrontal cortex [295] following therapy. Successful TFP may improve verbal memory, information processing speed, and executive functioning, related to decreased PTSD symptoms, especially in those with comorbid MDD [296]. Improvements in biomarkers, such as DNA methylation changes [175], have also been reported.

Psychological symptom reduction outcomes associated with TFPs are numerous. “Sudden gains”, defined as large, rapid, and stable symptom reduction in a one-session interval, are associated with greater expression of negative emotion [297] and enhanced therapy engagement [298]. Decreases in negative posttraumatic cognitions have been reported following successful therapy and may be a factor mediating differential therapy outcomes in military versus civilian populations [299]. TFPs can also reduce symptoms of depression and anxiety, and improve quality of life [275, 300], with secondary analyses indicating that PTSD and depressive symptom reduction are reciprocal [301, 302]. Treating depression may reduce comorbid PTSD symptoms, and vice versa [303], even when the treatment does not specifically focus on traumatic memory or address depressive symptoms [302], as in the case of non-trauma-focused PTSD therapies and pure exposure therapy, respectively. Further, TFPs such as EMDR have been used specifically to treat Major Depression and other disorders [304-306], and are associated with increased measures of posttraumatic growth even if posttraumatic growth is not specifically addressed by the therapy [307].

## 4 CHALLENGES FOR PTSD TREATMENT

### 4.1 Individual and Systemic Barriers to Good Outcomes

Despite the incredible advances in the understanding and treatment of PTSD, formidable challenges remain. PTSD in clinical practice is often complex, heterogeneous, and difficult to treat. Even TFPs, which have the most robust effect sizes of all currently accepted PTSD treatments, are inadequate for the majority [308], with worse outcomes in military samples [309], where two-thirds retain their diagnosis posttreatment [5]. Significant residual symptoms can follow even successful treatment, especially sleeping difficulties, hypervigilance, concentration problems, and nightmares [310-312]. An estimated quarter to a third of patients who receive TFP drop out, with even higher rates in some studies [313]. Psychopharmacological treatments fare even worse, with few evidence-based treatments offering more than modest benefit that often doesn't justify the adverse effects, cost, and long-term treatment.

Therapeutic challenges may stem from a myriad of intra and interpersonal, trauma-related, social, and systemic factors [314-316], often not directly related to fear conditioning or extinction, and exist even within publicly funded civilian and military systems of care [317]. Poor access to mental health care, which is often delivered in piecemeal fashion and lacks systematic symptom assessment and monitoring, misses the opportunity to promote early appropriate treatment when the illness is more responsive [19, 145]. This may be compounded by tendency towards minimization of prodromal symptoms, stigma, confidentiality concerns, poor social supports, impact of disability awards or secondary gain, perceived treatment inefficacy, and practical barriers to treatment such as insurance, finances, work schedules, childcare, competing priorities, and transportation [5, 318, 319]. Values, beliefs, past experiences with treatment, discomfort with the therapist, medical care needs and illness burden may also impact help-seeking behavior [320].

Patient complexity can present other challenges. Premorbid personality, attachment, and psychological defense styles can impact therapeutic engagement, which can interact with overtaxed systems and clinicians ill-equipped to deliver trauma-informed care. Trauma-related shame and guilt, especially in the context of relational trauma, are associated with and dynamically linked to greater PTSD symptoms [321, 322]. Psychiatric or medical comorbidities, especially MDD, BPD, substance use disorders, and brain injuries often complicate PTSD treatment. Depression, shame, guilt, anxiety, and chronic hyperarousal have been associated with worse outcomes. Such comorbidities can worsen executive dysfunction [323], emotional dysregulation, and dysfunctional coping, such as behavioural avoidance, social withdrawal, substance use, or suicidality, and lead to both poor engagement in and lower responsiveness to treatment. [324-327]. For some individuals, such as those with difficulty accessing and verbalizing traumatic memories, some first-line treatments may be especially challenging [328]. More extensive childhood trauma may predict higher baseline PTSD symptoms and somewhat lower impact of manualized, time-limited TFPs [329]. As the illness progresses, occupational, interpersonal, and social functioning becomes impaired, and sufferers often lose social and economic resources, such as jobs and relationships. This involves complex bidirectional relationships between PTSD and social fragmentation [330], including the impact of irritability and emotional numbing that can disrupt empathy and promote social withdrawal. These problems are often viewed as “comorbid” rather than part of a posttraumatic spectrum of illness progressing over time, partly due to fragmented systems of care, the acute, single-event conceptualization of PTSD, and its heterogeneous, fluctuating illness course. Unfortunately, treatment is frequently only offered or available at the chronic stage, when avoidance, chronic inflammation, multimorbidity, and emotional dysregulation are entrenched, and treatment outcomes are suboptimal. This can add demoralization to rigid negative belief systems that keep sufferers in a perpetual state of negative emotion and chronic stress. It is no wonder that many feel broken and become depressed, hopeless, and even suicidal.

Clinician factors play a role, including underdiagnosis of PTSD, challenges establishing therapeutic alliance [331], and availability of skills and training to provide quality TFPs [320]. PTSD is especially underdiagnosed in conditions like BPD or psychosis where other aspects of the illness are given priority and PTSD treatment is rarely offered, despite evidence for safety and efficacy in these populations [306, 332, 333]. This interacts with patient factors, such as physical and psychiatric comorbidity, denial, compensation, shame, and self-medication [333]. Avoidance and numbing symptoms can also lead clinicians to underestimate severity. Presentations with prominent physical symptoms, rather than psychological distress, are often unrecognized and poorly treated. This is true despite their central role in both PTSD and MDD, representing the physiological dysregulation that drives such disorders [17]. In addition, even when clinicians have the training, many do not use their skills in practice, impacting access [334]. Therapy protocol drift can occur and may represent a form of avoidance on the part of the therapist or patient. Lack of standardized symptom monitoring in usual practice makes it difficult to detect the 20% minimum reduction in symptoms in the first 8 weeks that is predictive of subsequent change [335]. Even if this were in place, clinicians often have few referral options. Clinicians may not feel adequately trained or comfortable delivering TFPs, especially when workload is high, TFPs are perceived as inflexible, there is fear of client distress or suicidal ideation [336], or there is lack of support [337-339]. Clinicians may fear that TFP can negatively impact therapeutic alliance and retention. However, this has not been supported by the literature [340].

Clinical guidelines and clinical practices can also hamper optimal treatment. When utilized, guidelines may overemphasize certain evidence-based TFPs, which were largely developed based on exposure and cognitive models and may not address the full range of PTSD sequelae. Certain treatments used in practice are inadequately studied due to academic interest or practical barriers. Further, clinical trials of interventions based on preclinical research are often performed in chronic populations, without attention to subtypes [19], and with waitlist controls, which may overstate effectiveness and do not allow adequate comparison of interventions [259]. PTSD recommendations are also essentially the same for new-onset PTSD as for chronic PTSD, ignoring data on how chronicity and complexity impact treatment outcomes. Similarly, treatment guidelines do not address whether the treatment response is impacted by single or multiple trauma exposure. Guidelines also do not distinguish between different PTSD presentations or give adequate guidance on how to select treatment, when to abandon interventions, or how to address treatment resistance [19]. Another critical issue that has not been investigated is the role of inflammation as a determinant of treatment response. In MDD, background inflammation is a predictor of antidepressant response, where higher levels of inflammation as measured by C-reactive protein predicted treatment nonresponse to antidepressants [341]. No such study has been conducted for PTSD despite the demonstrated role of inflammation, especially as the illness progresses [342].

The following section highlights specific challenges complicating PTSD treatment that have been a focus of research and may impact treatment response. These include: a) the impact of emotional dysregulation, b) suicidal ideation, c) dissociation, d) substance use disorders, e) trauma-related shame, guilt, and moral injury, and f) challenges related to military trauma.

### 4.2 Emotional Dysregulation

As described in sections 2.4 and 2.5, PTSD is associated with emotional dysregulation, often oscillating between emotional over- and undermodulation, which may be a factor in PTSD maintenance [343]. There is likely a bidirectional relationship, with emotional dysregulation both predisposing to and maintaining PTSD symptoms, especially for those with childhood adversity [63, 344, 345]. Emotion regulation deficits in PTSD include impaired distress tolerance [345], poor emotion recognition [344], and distorted trauma-related cognitions [346]; the relationship between distress tolerance and PTSD symptoms may be moderated by number of traumatic event types experienced (*i.e.*, trauma load) [345]. Over time, emotions themselves can become avoided, disconnecting people from themselves and their loved ones. Nervous system dysregulation from trauma cues also challenges cognitive and social function, especially for those with alterations in consciousness such as dissociation. Shame, either related to the trauma or its sequelae, can further exacerbate emotional dysregulation, especially when there is agitation, aggression, or role failure (see subsection 4.6), and can lead to a sense of brokenness, hopelessness, or exhaustion, and potentially suicidality. It is not surprising that severe, complex PTSD is often comorbid with other diagnoses that also feature emotional dysregulation: mood, anxiety, dissociative, substance use and personality disorders.

Given these challenges, research concerning the safety and efficacy of TFPs in populations with severe PTSD and high rates of emotional dysregulation, such as CPTSD, PTSD-DT, BPD, and childhood sexual abuse, have been undertaken. It has been suggested that those with high degrees of emotional undermodulation might have difficulty tolerating TFPs, with potential for PTSD symptoms and distress to increase in the initial stages of treatment. However, even with BPD, a condition with high rates of suicidality and challenges in therapeutic engagement [347], studies utilizing TFPs are encouraging, demonstrating large effect sizes and no significantly higher risk for dropout [348]. One study, comparing Dialectical Behavior Therapy (DBT) for PTSD (DBT-PTSD) and CPT, reported both interventions significantly reduced PTSD and BPD symptoms, and dissociative symptoms improved following treatment [349]. This is similar to other reports that BPD symptoms decrease with trauma-focused treatment, both in populations with [306] and without comorbid PTSD [350, 351]. Zeifman *et al.* provide a review of this area [347]. It is much discussed whether multi-component, multimodal therapies, as compared with trauma-focused unimodal approaches, are more effective for people with complex PTSD. Although head-to-head comparisons are currently investigated, some caution is warranted as these two, often polarised, characterisations of trauma treatments could be more theoretical than real, given that some unimodal approaches in real life incorporate diverse interventions and, on the other hand, multicomponent therapies rarely exclude trauma-focused work [21].

### 4.3 Suicidal Ideation

It is well known that 90% of those who die by suicide suffer from a diagnosable mental disorder, especially MDD and SUDs [352]. That PTSD is associated with substantial suicide risk is less widely appreciated [336]. Baseline or treatment emergent suicidal ideation can impact access to and engagement in trauma treatment, given the associated distress and suicide-specific beliefs such as unlovability, unbearability, or unsolvability [353]. Numbing, risk-taking and anger symptoms of PTSD might theoretically increase the translation of ideation to action [354, 355]. Further, a historical lack of consensus regarding whether to include or exclude participants with suicidal ideation from psychotherapy clinical trials and, until recently, how best to report adverse events related to suicide risk [356] provided little guidance to support clinicians. Since TFPs can temporarily increase PTSD symptoms and emotional intensity, clinicians may be hesitant to use trauma therapy in those with suicidal ideation, which may be fostered by medicolegal concerns, clinical training and guidelines that encourage stabilisation strategies over trauma-focused work in this population.

Except for active suicidality, the literature appears to favour treating PTSD in those with suicidal ideation, at least for populations enrolled in clinical trials. A metaanalysis comparing results from 48 randomized controlled trials of PTSD psychotherapies, stratified based on their suicide exclusion criteria, indicated no significant difference in PTSD outcomes between the trials that excluded and those not excluding suicidal ideation. This suggests that the effects observed in clinical trials are not significantly impacted by suicidal ideation-related exclusion criteria and TFPs are likely effective regardless of its presence [356]. Further, TFPs for PTSD are associated with reductions in suicidal ideation [353, 357-360]. A recent systematic review on the impact of treatments specifically designed to treat PTSD, suicidality, or both, reported PTSD treatments were associated with reductions in both PTSD and suicide-related outcomes, with most studies focusing on CPT or PE [336]. Suicide-focused treatments also reduced suicide-related outcomes, but the findings were mixed regarding their impact on PTSD-related outcomes. Rather than avoiding TFPs completely, clinician training regarding assessment and management of suicidal ideation and how to integrate interventions into comprehensive care that also addresses capacity to cope, psychosocial factors, and comorbid conditions such as major depression, are needed. However, there remains a lack of research to inform individualized treatment selection [336].

### 4.4 Dissociation and Treatment Outcome

Dissociation is considered a marker of clinical complexity and treatment challenges. That dissociation may portend poor response to psychotherapy, including exposure-based TFPs, is based on the idea that exposure to traumatic cues would provoke emotional overmodulation, emotional numbing, impaired emotional learning, and poor access to the traumatic memory network [361, 362]. PTSD-DT is associated with greater psychopathology [363, 364], but not elevated re-experiencing symptoms [365], which are the predominant focus for many TFPs and pharmacotherapies. Therefore, a phased approach to trauma treatment has been traditionally suggested for complex populations, especially those with dissociation, wherein patients are stabilised, provided psychoeducation, and taught emotion regulation and coping skills before undertaking trauma-focused memory work [366, 367]. Others argue that the phased model only delays effective TFP [368, 369].

The literature on dissociation and its impact on treatment outcomes, however, are mixed. Some studies demonstrate worse psychotherapy outcomes in those with PTSD-DT or high levels of dissociation [363, 370], and others indicate no difference, including in military samples [365]. A number of studies have reported that dissociative symptoms, including depersonalization and derealization, do not moderate treatment effects for NET [371], EMDR [364, 372], or intensified treatments for PTSD [364, 372]. A recent systematic review [373] reported that those with PTSD with dissociative symptoms benefited from trauma treatment and that both PTSD and dissociation symptoms lessened with treatment. This makes sense if dissociation is viewed as a defense against awareness of painful emotional or sensory stimuli; successfully addressing the threat should reduce the need for dissociation to reduce awareness. Additionally, despite concerns to the contrary, exposure treatments have not been found to be harmful to those with greater dissociative symptoms, nor were there substantially greater dropouts [373]. However, generalization of these findings is difficult; those with the highest levels of dissociation and those who are at risk of engaging in self-harming behaviors may not be represented in studies [373].

Overall, current research indicates that complex trauma populations do not necessarily need to be excluded from PTSD treatment, especially TF-CBT, EMDR, NET, and modified interventions specifically designed for complex populations [363]. CPTSD and PTSD-DT patients often have higher baseline symptoms, but TFP is often at least as effective, emotion dysregulation does not necessarily prevent TFP benefit [374], and emotional and self-regulation often improve after receiving TFP [364, 375]. However, more research is needed regarding how to best optimize or modify treatment to suit these often difficult-to-treat populations [376, 377], taking into account specific biological, psychosocial, and treatment factors.

### 4.5 Substance Use Disorders

Comorbid PTSD and SUD is associated with serious treatment challenges, including an increased likelihood of dropout and treatment resistance [325, 378]. Greater impairment [325, 378] and neurocognitive deficits in verbal and working memory, as well as poorer coping [379], have been reported. Despite recognition that comorbidity is high and worsens outcomes for both disorders, early trials of TFP for PTSD excluded those with SUD, limiting the evidence base in this population. Emotional dysregulation contributes to the maintenance of both SUDs and co-occurring PTSD and may predict response to TFP [380]. Characteristics of the substance may also play a role if attention, emotional engagement, or cognition is impaired.

Studies of TFPs in populations with SUDs include those with standard TFPs, modified TFPs [381], and therapies that incorporate trauma-focused modalities with concurrent treatment for substance abuse or emotional dysregulation [382]. Some of these studies demonstrate good outcomes for both PTSD and SUD, even in severely symptomatic populations [381, 382]. However, a recent metaanalysis indicated that PTSD psychotherapy treatment outcomes are generally worse in populations with comorbid SUD, where dropout rates may be higher and additional interventions to also focus on the SUD may be warranted [378]. Low quality evidence, dropouts, and poor treatment engagement, however, are confounders [378]. Growing evidence supports the “integrated model” over the “sequential model”, such that it may be preferred for patients to simultaneously receive treatments for PTSD and SUD rather than be abstinent prior to engaging in PTSD treatment [383]. There is no pharmacological treatment that targets both PTSD and SUD, and new medications are in high demand. Individuals with earlier trauma, emotional dysregulation, or other characteristics may require additional or unique clinical attention to improve their SUD outcomes [384].

### 4.6 Moral Injury

Moral Injury (MI) [385] has emerged as one of this generation's contributions to understanding and addressing psychological injuries [386, 387]. An evolving concept that is not currently included in the DSM or ICD, MI can be described as a syndrome characterized by trauma-related guilt and shame, intrusive thoughts, anger, and self-condemnation [388] that results from a violation of deeply held morals, ethics, or values [389]. MI may arise as a result of exposure to potentially morally injurious experiences (PMIEs), including perpetrating, witnessing, or failing to prevent an act(s) that transgresses one’s core beliefs [389-391]; acts of commission (*i.e.*, what one has done); acts of omission (*i.e.*, what one has not been able to do); and/or betrayal by a trusted authority [385, 392, 393]. Dynamically linked to greater PTSD symptoms [321, 322, 394], MI is associated with intense emotional, cognitive, and physical reactions [389, 395-399] and can lead to a degree of persistent psychological, social, and spiritual impairment and harm that is significant to extreme [388, 389, 392, 400-405]. The development of MI can parallel that of PTSD [392] and result in a fracturing of personal morals, beliefs, values, character, and relationships [385].

The link between PTSD and MI has been debated, including diagnostic boundaries, especially when trauma is also morally injurious. Overlapping features of PTSD and MI include anger, depression, anxiety, insomnia, nightmares, self-medication [405-407], severe suicidal ideation, and increased suicide attempts [406-411]. Bryan *et al.* separated a PTSD symptom profile that included re-experiencing symptoms, hyperarousal, insomnia and memory loss from MI, which included guilt, shame, anger, anhedonia and social alienation [407]. Predominantly cross-sectional and military-focused studies have found perpetration-based MI in service members with PTSD to be associated with higher levels of PTSD symptoms, compared to those with “life-threat” traumas. For work-related PMIEs, it has been estimated that PMIEs accounted for 9.4% of the variance in PTSD, 5.2% of the variance in depression, and 2.0% of the variance in suicidality [412, 413]. For veterans and active duty military members, MI is correlated strongly with suicide risk, loss of trust, and self-condemnation [386]. High quality studies are yet needed, however, to more clearly understand the relationship between MI, moral distress, and PTSD.

Populations with which MI is associated continue to broaden. Much of the literature has been focused on military populations, with some literature exploring conceptualization and interventions in healthcare, public safety personnel, education, social service settings, and leadership [392, 393, 405, 405, 412, 414-423]. During the COVID-19 pandemic, MI and moral distress have increasingly been linked to emergency and healthcare services. See Griffin *et al.* (2019) for a review of MI in healthcare professionals and its differentiation from moral distress [414].

Addressing MI may well be critical to facilitating recovery from trauma and chronic or treatment-resistant PTSD. Different treatment approaches may be needed based on conceptualizations and presentations of MI among populations exposed to diverse PMIEs. Further, interventions need to be informed by mediators and moderators of, and factors associated with, MI. Mediators include lack of social support, negative cognitions, and meaning-making. Self-compassion and forgiveness, pre-deployment mental health, education, and mindfulness are reported moderators [412, 424]. Organizational, environmental, cultural, individual, and relational factors also need to be considered. Means of addressing MI in organizations and clinical practice, however, raise ethical and clinical concerns in light of cultural norms and the lack of MI-specific training among many helping professions [414]. Potential treatments for MI will be discussed in section 5.4.2.5 (Spiritual and Moral Injury Interventions).

### 4.7 Military PTSD

Military PTSD populations are known to have worse PTSD treatment outcomes [5, 398, 425-427] and often present with non-fear emotional responses [428]. This has led to the recognition that the traditional “fear-based” paradigm of PTSD may not wholly explain the behaviours and suffering of veterans [385, 429]. Internal conflict, guilt, and ongoing distress resulting from military combat [430], moral transgressions [401, 404, 431], and disintegration of personhood [400, 432] are potential drivers for enduring posttraumatic stress and suicide risk [407, 410, 431]. With the number of deaths by suicide outnumbering those killed in action among returning service members in the US and other NATO nations [433], a greater understanding of military service members’ experiences of PTSD and MI is needed. This may apply equally well to other populations.

After receiving the best evidence-based treatment for PTSD, about two-thirds of veterans still meet criteria for PTSD [5]. An Australian latent growth mixture modelling study of military and veteran PTSD across 14 hospitals reported five PTSD outcomes: a) one-third have the most severe PTSD at intake and report minimal change over time, b) a small group (3%) report severe PTSD but large clinical change over time, c) about half have slightly less severe PTSD but large to excellent clinical change, d) and a small group (6.7%) have the least severe PTSD at intake and a large treatment effect. Depression and guilt were found to predict differences in response trajectories, indicating their importance for treatment [324, 434]. Another latent class analysis of heterogeneity in PTSD treatment response for 960 UK veterans following a residential trauma-focused intervention similarly found that: a) 71.3% showed positive treatment responses, b) 1.2% showed initial improvement but later relapsed, and c) 27.5% had treatment-resistant illness. This last group had higher pretreatment PTSD symptoms (re-experiencing, avoidance, and hyperarousal), depression, and anxiety [435].

Potential reasons for worse outcomes in military populations are numerous. These may include greater prevalence of premorbid early childhood trauma, emotion regulation deficits, higher number of traumas experienced, the impact of MI, extent of exposure to death and suffering, comorbid complicated grief [436], and features of the military culture and organizational structures [437]. Veterans with lifetime PTSD are reported to show impaired recognition of all emotions compared to trauma-exposed veterans without PTSD, even after adjusting for confounders such as depressive symptoms. Their offspring may also show impaired emotion recognition, especially for happiness and disgust, raising the possibility of intergenerational transmission of emotional dysregulation [344]. Dissociation is also a prevalent phenomenon among veterans with PTSD that may interfere with treatment effectiveness [365]. Both hyperarousal and pre-deployment dissociation may be predictive of disease severity, and re-experiencing may be a predictor of suicidality in veterans with combat-related PTSD [434]. Military groups are also associated with cognitive avoidance [438], trauma-related guilt, and compartmentalization [439], which may allow military members and veterans to avoid emotionally processing traumatic material needed for recovery [440].

The impact of specific trauma types encountered in military service may mediate or moderate PTSD severity. War zone traumas are heterogeneous and may be compounded by “non-life-threat traumas”, such as MI or loss of comrades. Moral injury, as previously described, is highly associated with guilt, shame, self-blame, and greater peri- and posttraumatic betrayal and humiliation symptoms [441], relative to “life threat” traumas. Traumatic loss is correlated with greater avoidance, guilt, or sense of responsibility, and greater peri- and posttraumatic sadness. Complex grief has also been associated with lower PTSD treatment response and likelihood of remission, greater PTSD severity, increased trauma-related guilt, and three times greater risk of suicidal ideation [436]. Military sexual trauma (MST) is a further high risk trauma type, impacting at least 13% [148]. MST is associated with a seven-fold increase in PTSD, over twice the risk for depression and suicidal ideation, and multiple medical conditions including sleep disorders, such as insomnia and sleep apnea, and chronic pain [148, 353].

Questions have arisen about the extent to which therapies for these populations should be adapted. For example, ought military-related PTSD be considered as 1) a disorder that can be reliably managed by brief (approximately 12 session) monotherapies, or 2) a highly complex and multiform condition requiring more individualized and comprehensive intervention? As has been advocated and will be seen in the later sections of this paper, treatment innovations in this population include enhancing existing treatments, emerging non-trauma-focused interventions, novel pharmacotherapy, personalized medicine approaches, advancing functional outcomes, family intervention and support, and attention to physical health [442].

## 5 EMERGING AND NOVEL APPROACHES TO THE TREATMENT OF PTSD

The following section describes current emerging and novel approaches to PTSD treatment. They will be described in terms of their modality, timing, and individual components. The evidence for these interventions is different across the spectrum. They have been included based on their potential and justification to the current knowledge base of PTSD. Strengths and weaknesses will also be discussed.

### 5.1 The Changing Landscape of PTSD Interventions

Optimizing, combining, and developing new treatments is critical to addressing poor treatment outcomes in PTSD. This necessitates a broader conceptualization of PTSD, attention to its life course and potential subtypes, and recognition of differing pathophysiology. Improved assessment and prediction models [443], including development of biomarkers, are also imperative for identifying those at risk and providing early intervention using optimized approaches. Preclinical research to further clarify subtypes and mechanisms beyond fear circuitry will be important to find new biological targets. The following sections review novel and emerging approaches to address PTSD treatment, including early intervention and secondary prevention strategies, emerging psychopharmacological, psychotherapeutic, and behavioral approaches, as well as medication and psychedelic-assisted psychotherapy, neuromodulation and stellate ganglion blockade. Table **5** provides brief descriptions of these modalities.

### 5.2 Early Intervention and the Golden Hours

Early PTSD secondary prevention work focused on treating Acute Stress Disorder, or those at high risk of PTSD, with abridged TFPs such as EMDR, PE, or TF-CBT interventions. This is efficacious for reducing development of PTSD, depression, and anxiety symptoms after trauma [256, 444]. Offering support, safety, information, and practical assistance (psychological first aid) is also generally supported. Psychological debriefing, however, is not recommended in the first 3 months following a trauma, based on evidence that it may be either ineffective or potentially harmful [256]. Work to prevent PTSD on a population level includes resilience training prior to trauma exposure, and interventions aimed at reducing intergenerational transmission of trauma such as reducing adverse childhood experiences. However, this area is complex, as adverse childhood experiences tend to co-occur and parental psychopathology also needs to be addressed, which is both costly and practically challenging.

Machine learning [445] and other predictive models are expected to amplify precision. Machine learning may assist with identifying those at risk, more clearly understanding the heterogeneous clinical and biological aspects of the illness, and developing precision medicine approaches to PTSD treatment. Work to incorporate subtle linguistic and prosodic information from audiotape or written language samples is underway to find meaningful markers of PTSD and other psychiatric illnesses [446]. Machine learning, by combining the now confusing array of biological, genetic, imaging, and historical data into machine learning algorithms, is likely to be used to establish clinically useful subtypes, more accurately distinguish PTSD from other illnesses, predict outcomes for specific treatments, and allow individualized treatments [445].

The “Golden Hours” is a concept from acute medicine being given consideration for PTSD treatment. The Golden Hours denote the time-limited window of opportunity to administer an intervention to prevent disease, after which it may be either ineffective or harmful [447]. For example, there is a 3-hour window during which thrombolytics have a positive risk-benefit profile for preventing acute stroke [448]. In the case of PTSD prevention, such interventions could theoretically capitalize on peritraumatic vulnerability of reactivated memory traces during reconsolidation, thus interfering with storage of acquired fear. In these early hours, short-term memory traces of the trauma are unstable but at risk of being transformed into a long-term, emotionally distressing memory by high catecholamine levels and failure to mount negative feedback HPA mechanisms. As discussed previously, this can create a vicious cycle of traumatic memory “overconsolidation”, generalization of threat, stress sensitization and chronic stress response. However, at other times or under other conditions, repeated reactivation of memory through recall may entrench the encoding process. This may explain why some interventions such as psychological debriefing and benzodiazepines might worsen PTSD symptoms if given during this early time window. Psychological debriefing may increase accessing of trauma memory and therefore memory consolidation, interfering with normal recovery. This contrasts with TFPs, which theoretically help create a coherent rather than fragmented narrative and incorporate new, adaptive information into the memory network. Benzodiazepines, which interfere with HPA axis activation and may prevent mounting of an appropriate stress response, have also shown to increase the risk of developing PTSD when given early after a traumatic event [449]. Therefore, context matters.

The most studied early pharmacologic interventions for the secondary prevention of PTSD include oxytocin, propranolol, morphine, and hydrocortisone. There is limited data for oxytocin. One randomized, double-blind prevention trial, using oxytocin (40 IU twice daily for eight days), started within 12 days of acute trauma, was negative but those taking oxytocin with higher initial PTSD symptom severity had lower PTSD symptom severity at up to six months follow up [450]. Propranolol, a beta-adrenergic antagonist, can reduce the adrenergic overdrive present in the hours or days after trauma exposure and has therefore been theorized to block noradrenaline-driven fear memory reconsolidation [205, 451]. Despite preclinical data that propranolol may reduce subsequent reactivity to trauma cues, human studies so far have been disappointing [205, 451].

Morphine, an opioid agonist, inhibits the locus coeruleus and therefore noradrenaline, similar to propranolol. Morphine injections into the amygdala impair memory for fear conditioning in animal studies [452]. Those taking opiates during or immediately after trauma exposure have been reported to be less likely to develop PTSD, or to develop milder symptoms, than those not taking opiates [453-456], in both civilian and military samples, although contradictory studies also exist [457]. One observational study compared nitrous oxide against morphine during childbirth and found nitrous oxide administration predicted reduced PTSD symptom severity, for those who later developed it. Nitrous oxide was superior to morphine, which had a positive but non-significant benefit; however, both drugs predicted increased PTSD symptoms when combined with severe pain during labour [457]. Nitrous oxide has NMDA antagonist properties, and is thought to also interfere with memory consolidation through reduced long-term potentiation [457, 458]. Morphine and nitrous oxide might also have a protective effect by reducing peritraumatic pain, which is a risk factor for PTSD development. However, both morphine and nitrous oxide might also, theoretically, contribute to memory disorganization during the encoding process, worsening symptoms. Human RCTs to explore this issue are lacking.

Hydrocortisone administration has also been attempted for PTSD prevention since low levels of cortisol after trauma are associated with PTSD development [459-461], and cortisol reduces sympathetic nervous system activation and facilitates fear extinction. Cortisol, administered to humans immediately after exposure to a stressful event, results in fewer memories of that event [462, 463], in keeping with findings that PTSD develops in the context of faulty HPA axis negative feedback. Meta-analyses provide some support for the efficacy of hydrocortisone to reduce PTSD incidence when used in an acute preventative, rather than curative context, with small effect size [464], especially after high-risk surgery, sepsis, or severe traumatic injury [465]. Larger, high-quality studies are needed to confirm this finding and optimize the approach prior to more widespread application.

Trials of dexamethasone, docosahexaenoic acid [466], escitalopram, imipramine, chloral hydrate, and gabapentin have been negative [467, 468]. Other potential interventions being investigated include losartan [469], N-acetyl cysteine, and corticotropin-releasing factor (CRF) type 1 receptor antagonists [470]. Preclinical trials investigating targets for disrupting memory reconsolidation include NMDAR antagonists, glucocorticoid receptor antagonists, 5-HT5A antagonists, 5-HT6 receptor agonists, GABA receptor modulators, CB1 agonists such as anandamide, MEK inhibitors, mTOR inhibitors and inhibitors of protein synthesis, protein kinases, and transcription factors [205]. In addition to these biological factors, the conditions and timing under which these medications are used are crucial [206, 207]. Further, it is not known whether there are “boundary conditions” that interfere with memory labilization, such as the age or strength of the memory [205]. This is not only important for “Golden Hours” emergency treatment, but also for psychotherapy (to be discussed in a later section). Larger human clinical trials, focusing on clinically relevant outcomes and taking patient factors into account, such as rumination or neuroticism, that might impact frequency of memory reactivation, are needed.

Some guidelines for PTSD now consider interventions targeted at specific symptoms in some circumstances when other interventions are not available. Holmes and colleagues developed a preventative approach targeting just one core clinical feature, intrusive memories after trauma [471]. This brief behavioural intervention to reduce intrusive memories soon after trauma, labelled a ‘cognitive vaccination’, draws from cognitive task interference and memory (re)consolidation theory, rather than traditional exposure models. A brief memory reminder or orientation to re-/activate specific trauma memory ‘hotspots’ is followed by a visuospatial cognitive interference task (*e.g.*, playing a computer game ‘Tetris’ alongside training to engage in ‘mental rotation’ throughout), and is administered according to specific timings and order. Recent results are supportive of changing dysfunctional appraisals as a modifiable cognitive mechanism, and that their proximal modification transfers to downstream PTSD symptoms [472, 473].

Such ‘Golden Hours’ interventions are also relevant for situations such as terrorism, as there are recognizable actions that should and should not be taken. A recent NATO group has developed a first aid to terror web app (firstaidtoterror.com) that offers guides for early interventions with children, adults, the military, and for media and policymakers [474].

### 5.3 Emerging Pharmacological Treatments

#### 5.3.1 Cannabis and Cannabinoids

Cannabis is commonly consumed globally, including anecdotally for PTSD symptoms, with both academic and public interest in its potential for PTSD. The human endocannabinoid system plays an important role in the regulation of mood, pain perception, appetite, learning and memory, and inflammation [192-194, 475]. Important components include endogenous CB1 and cannabinoid type 2 (CB2) receptors, their endogenous cannabinoid ligands (anandamide and 2-arachidonoylglycerol), and enzymes that synthesize and degrade these cannabinoids [476]. Key compounds in the cannabis plant include ∆9-tetrahydrocannabinol (THC) and cannabidiol (CBD). THC, which primarily activates the CB1 receptor, is the main psychoactive and intoxicating component of the Cannabis plant and contributes to the “high” that recreational users seek. CB1 receptors, which are downregulated after chronic stress [193], are concentrated on presynaptic CNS terminals and play a role in sleep through a connection to the HPA axis [193]. Nabilone, a synthetic cannabinoid used in clinical practice, is a THC analog [477]. CBD is a non-psychotomimetic cannabinoid found in cannabis with lower CB1 and CB2 affinity and inverse agonism at the CB2 receptor, with purported neuroprotective, analgesic, sedative, antispasmodic, anti-inflammatory, and anxiolytic properties [478].

The potential role of cannabinoids in PTSD stems from the overlap between PTSD pathophysiology and the role of the endocannabinoid system. CB1 receptors are found throughout the limbic system and modulate mood, stress, learning, and memory. PTSD and associated intrusive symptoms are linked with reduced peripheral anandamide, abnormal CB1 receptor-mediated anandamide signaling, and compensatory increase of CB1 receptor availability [479]. Cannabis is thought to modulate threat-related processing and reconsolidation of fear-based memories [480] through CB1 receptors in the amygdala, PFC, and hippocampus, thereby decreasing hyperarousal and potentially blocking fear memory consolidation, increasing serotonin in the prefrontal cortex, and improving neurogenesis and mood [481]. However, preclinical studies have shown both CB agonism and antagonism associated with disrupted fear memory reconsolidation, hinting at complex interactions [205]. THC lowers hypothalamus reactivity, increases mPFC activation during threat, and increases mPFC-amygdala functional coupling. Cannabinoids may increase BDNF in the hippocampus and the basolateral amygdala, altering long-term potentiation in hippocampal neurons. For a review of the potential role of cannabinoids for PTSD, see Orsolini *et al.* (2019) [479]. Current clinical literature supporting cannabinoids is preliminary and includes case reports and observational and retrospective studies of THC, nabilone, or THC in combination with CBD. Potential benefits include reduction in PTSD symptoms, PTSD-related sleep disturbances, emotional reactivity, and improved quality of life [477, 479, 482-486]. However, impact on PTSD symptoms is variable, with some reports of minimal impact or symptom exacerbation [487]. Dry mouth, headache, agitation, and euphoria are common adverse effects [485].

Concerns about long term risks, including Cannabis Use Disorder, worsening of intrusion symptoms and psychiatric comorbidity, including substance use, suicidal ideation, and suicide attempts, emergent psychotic states, and cognitive deficits, warrant caution [488-494]. There may also be a theoretical risk for worsened dissociative symptoms for those with CPTSD [495]. Chronic THC consumption may impair learning and memory and is associated with grey matter atrophy [496]. Further, there is concern that many PTSD sufferers use cannabis as an avoidant coping strategy, in an attempt to numb responses to trauma reminders and reduce symptoms such as negative emotions, diminished interest, social withdrawal and lack of positive emotions [497, 498]. Given the potential harms of cannabis, THC, and its analogs, CBD has garnered interest, given its proposed anxiolytic, anti-inflammatory, neuroprotective properties, and seemingly lower risk of psychoactive effects; human trials for PTSD have yet to be published [499]. Given the widespread use and legalization of cannabis and CBD in some countries, large-scale clinical trials to determine the safety and efficacy of all cannabinoid products should be established.

#### 5.3.2 Neurosteroids, Neuropeptides and Related Compounds

##### 5.3.2.1 INTRODUCTION

Under stressful conditions, the GR suppresses the ongoing stress reaction, for example by reducing the synthesis of Corticotropin Releasing Factor (CRF). Through negative feedback inhibition of the hypothalamus and pituitary by corticosteroids, the stress response is terminated, and normal basal glucocorticoid levels are restored. However, in PTSD, there may be insufficient corticosteroid at the time of trauma exposure to terminate the stress response and to counteract over-consolidation of traumatic memories mediated by sympathetic nervous system hyperactivation. Therefore, neurosteroids and other compounds impacting this process have been tested for PTSD prevention, monotherapy, and augmentation. These include corticosteroids such as hydrocortisone, the GR antagonist mifepristone, and CRF type 1 antagonists. This category also includes allopregnanolone, oxytocin, and neuropeptide Y (NPY) [500] (discussed in section 5.3.4.2).

##### 5.3.2.2 Hydrocortisone

Hydrocortisone, a corticosteroid receptor agonist, has been trialed for PTSD prevention, monotherapy, and augmentation, with and without psychotherapy [500, 501]. Not only does cortisol reduce the stress response, but it may reduce excessive trauma memory retrieval and reconsolidation. Preliminary findings favoring a single medium to high dose of intravenous hydrocortisone (100 to 120 mg), aimed at altering the trajectory of PTSD, have been reported. Typically, the window of opportunity would be limited to the first 6 hours after trauma exposure, and in the emergency room of a general hospital (see section 5.2 on early intervention). A systematic review of 8 studies including 362 participants showed a large effect for reducing posttraumatic stress symptoms in a preventative context, but not in patients with existing PTSD [464]. While hydrocortisone may be promising for PTSD prevention and augmentation of exposure therapy, pilot trials of oral hydrocortisone for chronic PTSD treatment have produced mixed results [500, 502, 503], and adverse effects would likely hamper its chronic use.

##### 5.3.2.3 Corticotropin Releasing Factor Receptor Antagonists and Glucocorticoid Receptor Antagonists

Mifepristone, a selective antagonist of the GR, increases cortisol and adrenocorticotropic hormone levels by attenuating negative feedback of the HPA axis and reregulating the balance of mineralocorticoid and glucocorticoid receptors. Mifepristone 600 mg/day for one week was reported to reduce PTSD symptoms and increase response and remission rates compared to a placebo. This was associated with increased cortisol and adrenocorticotropic hormone levels and decreased cytosolic GR in lymphocytes [504]. However, more recent RCTs were negative [505, 506].

Corticotropin Releasing Factor receptor 1 antagonists, such as GSK561679, also hold promise, as PTSD is associated with elevated CRF, and CRF agonism induces fear and PTSD-relevant anxiety responses [507, 508]. GSK561679 350mg/day for 6 weeks has been shown to inhibit fear-potentiated startle, but not fear acquisition or altered discrimination between threat and safety cues, in female PTSD participants [470]. Another prior RCT reported similar results, with no overall efficacy but a positive signal for subjects with early life adversity who were also GG homozygotes for rs110402 [509]. Subsequent analysis demonstrated that outcomes were predicted by DNA methylation patterns at the *NR3C1* and *FKBP5* genes, relevant to PTSD, with significant differences in *CRHR1* methylation patterns after GSK561679 treatment in the subgroup of patients with high CRF activity. NR3C1 baseline methylation significantly interacted with child abuse to predict PTSD symptom change following GSK561679 treatment [510]. This result emphasizes the importance of determining PTSD subtypes and factors that impact treatment response of individual interventions.

##### 5.3.2.4 Allopregnanolone

Allopregnanolone, a progesterone metabolite synthesized in the brain that acts at GABA type A receptors, inhibits the HPA axis [195] and has anxiolytic, sedative, anesthetic, analgesic, and anticonvulsant effects [195, 511]. Alterations in allopregnanolone and its stereoisomer pregnanolone have been found in PTSD. These include lower cerebrospinal fluid levels in premenopausal females [512] and lower plasma allopregnanolone and pregnanolone to 5α-dihydropro-gesterone ratios [513]. Allopregnanolone and pregnanolone to dehydroepiandrosterone ratio have also been associated with re-experiencing and depressive symptoms [512]. In animal studies, allopregnanolone induced by norfluoxetine can attenuate contextual fear responses [514], with similar findings for ganaxolone, a synthetic analogue of allopregnanolone [515]. In clinical neuroimaging studies, allopregnanolone was associated with reduced activity in the amygdala and insula and with increased activity in the dorsal medial prefrontal cortex; there was also enhanced connectivity between the amygdala and dorsal medial PFC, a region connected to emotion regulation circuits. Interestingly, this finding was also related to reduced self-reported anxiety [513].

Poor oral bioavailability has led to indirect attempts to raise brain allopregnanolone levels, including through SSRIs like fluoxetine or translocator protein (TSPO) ligands. TSPO facilitates allopregnanolone synthesis by transport of cholesterol into mitochondria, from which allopregnanolone is synthesized [516]. TSPO agents such as AC-5216/XBD 173, etifoxine, and YL-IPA08 may have antidepressant and anxiolytic effects [515]. In animal models of PTSD, TSPO ligands reduced anxiety and contextual fear with increased allopregnanolone levels present in serum, the prefrontal cortex, and hippocampus [517, 518]. Oral ganaxolone, a synthetic 3β-methylated analog of allopregnanolone, was trialed in phase II clinical trial, the only human study related to allopregnanolone for PTSD [519]. This trial was negative but suffered from subtherapeutic blood levels.

##### 5.3.2.5 Oxytocin

Oxytocin is a hypothalamic neuropeptide with a wide range of actions spanning reproduction, social bonding, and regulation of stress and the autonomic nervous system [500, 520]. With respect to PTSD, oxytocin has impacts on traumatic memory and PTSD-associated brain networks and has purported benefits for facilitating social interactions, goal-oriented cognition and behaviour, and improving working memory and executive function [521]. Its receptors are located in the amygdala, brainstem, olfactory nucleus, and anterior cingulate cortex [522]. This coincides with reports that oxytocin may lower coupling between the amygdala and brain regions related to behavioral and autonomic fear expression, such as the periaqueductal gray and reticular formation [523-526]. In human studies, intranasal oxytocin modulates threat salience among childhood trauma-exposed individuals [527], reduces amygdala reactivity in response to emotional faces in PTSD patients [528], and is implicated in the formation and consolidation of intrusive memories [529]. Oxytocin may also increase dlPFC connectivity among individuals with PTSD, relative to trauma-exposed controls, and connectivity between dlPFC and ACC for those with PTSD, compared to when taking placebo [521]. This increased top-down prefrontal control and resulting decreased autonomic and endocrine fear responsiveness may explain its anxiolytic effect [500, 530]. Oxytocin also seems to increase the connectivity between the amygdala and regions related to social cognition and empathy, such as the mPFC and the insular and inferior frontal gyri [526, 531]. Reports indicate that while anterior insula response to social rewards is reported to be decreased in PTSD patients, intranasal oxytocin administration normalizes this and further enhances the right putamen response, which is associated with self-referential processing and both the DMN and CEN. This improved responsiveness to social rewards is theorized to result in enhanced trust and therapeutic alliance and therefore better psychotherapy outcomes [532, 533].

Oxytocin has been investigated in clinical studies for secondary prevention (as discussed previously in section 5.2), as a pharmacologic treatment, and as an adjuvant enhancing exposure interventions [533], which will be discussed later in section 5.5.4 on medication-augmented psychotherapies. Clinical studies related to monotherapy for PTSD treatment are mixed and may be influenced by certain aggravating influences on the acquisition and consolidation of traumatic memories, and possible influences from sex and epigenetic factors [529]. Related to sex and epigenetics, baseline salivary oxytocin levels are reportedly lower in male, but not female, police officers with PTSD [534], and there may be a relationship between oxytocin receptor gene (OXTR) polymorphism and PTSD, which may also interact with attachment style [535]. Oxytocin is also associated with increased prefrontal control over the centromedial amygdala in males but decreased salience processing of the dACC and basolateral amygdala in females [528, 536]. With respect to traumatic memories, a double-blind, placebo-controlled study of healthy women who received either oxytocin or placebo before exposure to a trauma film paradigm, designed to elicit intrusive memories, demonstrated that oxytocin actually induced significantly more intrusive memories than the placebo condition. This effect of oxytocin on intrusive memories was influenced by biological covariates, such as salivary cortisol, heart rate variability, and PTSD polygenic risk scores. Higher polygenic loadings for PTSD and MDD were directly associated with a higher number of reported intrusions after exposure to the trauma film stressor, suggesting that intranasal oxytocin may amplify the acquisition and consolidation of intrusive memories, depending on neurobiological and genetic factors [529]. Until more studies are completed to understand these complexities, oxytocin for chronic PTSD treatment remains premature for routine clinical care.

#### 5.3.3 Ketamine and Other Glutamate Modulators

Ketamine, a NMDA receptor antagonist and “dissociative anesthetic”, has received notoriety because of seminal work demonstrating rapid onset antidepressant effects and antisuicidal potential, including for treatment-resistant MDD [537]. The term “dissociative” here is distinct from trauma-related dissociation. Ketamine is a stimulant that achieves anesthetic effects not through sedation, like other anesthetics, but through dissociation or disconnection between mind and body, as the person cannot receive information from the senses. However, sub-anesthetic doses produce other effects with implications for psychotherapy, which will be discussed later in the section on psychedelic-assisted psychotherapy. NMDA receptor activation has been found to increase the formation of depressive or anxious intrusive memories, through amygdala activation and long-term potentiation, promote reconsolidation of fearful memories upon recall and impact extinction processes, all potentially ameliorated by ketamine [205, 538]. Ketamine has also been shown to upregulate BDNF and subsequent synapse formation and neuronal connections through increased dendritic spine growth [537], promoting formation of neuronal connections damaged by chronic stress, especially in the PFC [539]. Increased prefrontal connectivity has been linked to ketamine and proposed as a mechanism for its rapid-acting antidepressant properties [540]. Ketamine activates the opioid system, and administration of naltrexone has been shown to partially attenuate antidepressant, but not dissociative, effects of ketamine, raising questions about the relative impact of these actions [541].

Given the high comorbidity of MDD with PTSD, and the theoretical benefits to the pathophysiology of PTSD, open-label and small RCT pilot trials of intravenous ketamine for PTSD have been conducted, with promising initial results that require further study [542]. Ketamine treatment regimens in these studies have ranged from a single dose of ketamine to multiple ketamine doses scheduled three times weekly, with doses ranging from 0.2 mg/kg to 1mg/kg. Most of these studies have shown, at least in the short term, rapid improvement of PTSD and MDD symptoms, with large effect sizes, in civilian and military populations with PTSD with or without comorbid MDD [542]. Ketamine is reported as well-tolerated, aside from dissociative symptoms, although relapse often occurs in up to an average of 20 to 40 days [543-549]. However, one larger double-blind, randomized, placebo-controlled, multicenter clinical trial (NCT02655692) with 158 active duty military and combat veterans with persistent antidepressant-resistant PTSD symptoms had less robust findings. Participants were randomized to three groups, each receiving 8 intravenous infusions: a) placebo b) low-dose ketamine (0.2 mg/kg), or c) standard dose ketamine (0.5 mg/kg), administered twice weekly. Outcome measures included PTSD and depression symptoms, measured at baseline, during treatment, and for 4 weeks after the final infusion. This study reported no difference in PTSD symptoms, but depression scores improved following ketamine treatment [548]. It may be that ketamine is better utilized as a catalyst for psychotherapy, rather than a standalone treatment, which will be discussed later in the section on psychedelic-assisted psychotherapy.

Research is beginning to emerge regarding other NMDA antagonists, including ifenprodil, xenon, and lanicemine (BHV-5500) [550-553], and other modulators of the glutamatergic system, including phenytoin, tianeptine, riluzone, and zonasamide [554, 555], indicating potential for reducing hyperarousal symptoms [553, 556]. Modulation of D-serine, a NMDAR co-agonist, is also being investigated in preclinical studies for facilitating fear extinction by increasing D-serine-mediated NMDA receptor transmission. Mechanisms include action on the alanine-serine-cysteine-1 transporter, D-amino acid oxidase enzyme (that breaks down D-serine), or serine racemase, which converts L-serine to D-serine [557].

#### 5.3.4 Analgesic, Anti-inflammatory, and Antioxidant Compounds

##### 5.3.4.1 Opioid Agonists, Opioid Antagonists, and Nitrous Oxide

While opioids and nitrous oxide have been administered just after trauma exposure for secondary prevention of PTSD, as discussed previously, few studies are published regarding the impact of these pain medications for treatment of established PTSD. Potential mechanisms include reduction of fear and pain, as well as interruption of memory reconsolidation [205, 457, 458]. One case series of nitrous oxide for veterans with PTSD demonstrated symptom reduction in two of three participants [558]. Further, there is interest in investigating buprenorphine, a partial mu opioid agonist and full kappa and delta opioid antagonist used clinically as opioid agonist therapy for opioid use disorder. A retrospective chart review of veterans with PTSD demonstrated superiority of opioids and buprenorphine in combination with naloxone, over SSRIs, in a veteran sample for reducing PTSD symptoms [559]. A similar, larger clinical trial is underway (NCT03605342). Another trial, comparing Suboxone (buprenorphine and naloxone) to long-acting injectable naltrexone, an opioid antagonist, for comorbid alcohol use disorder and PTSD is also in recruitment phase (NCT03852628) [560]. Finally, nalmefene, an opioid antagonist, has been investigated for dissociative symptoms in patients with comorbid BPD and CPTSD [561], which follows emerging reports of opioid antagonists being used for BPD and dissociative disorders comorbid with PTSD [562]. Theoretical support from animal and preclinical data suggests a role for the opioid system in dissociation [222], again recognizing the need to assess and study PTSD subtypes and symptom profiles.

##### 5.3.4.2 Neuropeptide Y

Neuropeptide Y (NPY) is a neurotransmitter regulator (*e.g.*, dopamine and glutamate) with myriad functions including pain, circadian rhythms, learning, memory, neurogenesis, neuroprotection and neuropsychiatric conditions such as depression, anxiety, and addiction [500, 563]. NPY expression is elevated in the brain regions related to fear, arousal, and threat detection such as the amygdala, hippocampus, periaqueductal gray, dorsocaudal lateral septum, and locus coeruleus [564], and in the sympathetic ganglia, adrenal medulla, and platelets [565]. NPY counteracts the action of CRF and has anxiolytic effects. In animal models, administration into the amygdala is associated with resilience in the face of stress [500]. Interestingly, an animal study found that the combination of brexpiprazole and escitalopram increased NPY in hypothalamic areas [566]. In humans, low NPY has been found in PTSD patients [567, 568], and levels are negatively correlated with PTSD symptom scores [568]. In a large prospective sample of Dutch service members it was found that pre-deployment plasma NPY levels were not associated with the development of PTSD; NPY trajectories were not associated with the development of PTSD over time and plasma NPY was not identified as a susceptibility marker for the development of PTSD [569]. NPY genotypes may have a relationship with PTSD symptom severity [570], emotional and physiological responsiveness to stress, and susceptibility to anxiety [571]. Poor blood-brain barrier penetration and formulation challenges present barriers to using NPY in clinical trials [572]. Recently, a small phase Ib, double-blind, dose-ranging RCT of single dose intranasal NPY (dose range 1.4 mg to 9.6 mg) for PTSD reported it was well-tolerated and there was a dose-response curve for reduction of Beck Anxiety Inventory scale scores [573].

#### 5.3.4.3 N-acetyl Cysteine (NAC)

N-acetyl cysteine (NAC), clinically used as an antioxidant and an antidote to drug toxicity, is now being considered for PTSD. Its pharmacologic effects include reduction of cytokine activity and inflammation, modulation of dopamine release, reversal of mitochondrial dysfunction, reduced apoptosis, increased neurogenesis, and increased glutamate release [574]. PTSD is overall associated with chronic oxidative stress, higher levels of proinflammatory cytokines and inflammatory mediators, such as IFNɣ, TNF-α, C-reactive protein, and IL-1β [189, 574], and alterations in the glutamatergic system [235, 242, 575]. In rodents, NAC reduces levels of inflammatory cytokines TNF-α, IL-1β, nuclear factor kappa, IL-6, and IL-10 [574]. Antioxidant activity of NAC occurs *via* synthesis of glutathione, enhancing glutathione-S-transferase activity, scavenging free radicals, and stimulating group II metabotropic glutamate receptors to decrease glutamate transmission [574]. Oxidative stress is implicated in many psychiatric disorders, including PTSD and MDD, and may lead to glutathione depletion, mitochondrial dysfunction, cell damage, and chronic inflammation. Antioxidants like NAC may theoretically, therefore, prevent development of later complications of PTSD driven by chronic inflammation (see Bradlow *et al.* (2022) for a review of NAC in psychiatric disorders [574]). Therefore, NAC may benefit PTSD by ameliorating oxidative stress, normalizing corticostriatal glutamate transmission and reducing levels of inflammatory cytokines [574, 576]. While studies are ongoing, one small double-blinded RCT utilizing NAC (2,400 mg/day) for veterans with comorbid PTSD and SUD, in combination with CBT, reported improvement in self-rated PTSD and depressive symptoms. However, these benefits were not sustained at the one month follow-up [576]. Two larger RCTs are underway [577, 578].

#### 5.3.4.4 Other Anti-inflammatory Compounds

Utility of other anti-inflammatory compounds is theoretical and awaits testing in PTSD. Minocycline has been shown to reduce cytokines in the hippocampus, PFC, and hypothalamus and attenuate anxious behavior in a rat model of PTSD [579]. Other candidates include ASA, non-steroidal anti-inflammatory drugs (NSAIDs), and doxycycline, which have yet to be formally tested in human trials [580-582].

#### 5.3.5 Other Psychopharmacological Targets

A variety of other pharmacological approaches are being investigated for PTSD and are in early stages of preclinical development. These include brexpiprazole, asenapine, nepicastat, and orexin receptor antagonist suvorexant [583-587] (Table **5**). For a review of orexin’s role in PTSD-like responses, see Kaplan *et al.* (2022) [588]. Memantine has also been investigated for cognitive impairment associated with PTSD, in veteran populations, and is associated with reductions in PTSD symptoms of numbing, avoidance and hyperarousal, better quality of life, and improved disability scores [589]. The following sections will focus on advances in psychotherapeutic and behavioral interventions.

### 5.4 Psychotherapeutic Innovations

#### 5.4.1 Developments in Optimizing Psychotherapeutic Treatments

Work to improve on current evidence-based treatments, combine TFP strategies, determine factors related to treatment response, and develop new interventions is challenging previous thought regarding essential elements needed to improve PTSD outcomes [590]. This work includes adapting interventions to improve engagement, efficacy, efficiency, and tolerability, to manage avoidance, dissociation and dropout rates, and to discern necessary from unnecessary elements [591-594]. Prolonged Exposure, for example, has been modified in the following ways: a) integration with cognitive restructuring or stress inoculation training, b) shortened exposure times and fewer exposure sessions, c) omitting components, such as homework or in vivo exposure, d) intensive schedules, e) remote delivery, f) virtual reality enhancement, g) adding imagery rescripting components, and h) combination with pharmacotherapy, such as SSRIs. These modifications had limited impact on effect size and dropouts, with the exception of adding cognitive restructuring [592, 595]. However, confounders abound. Factors such as therapy quality, methodological issues, and population characteristics make it difficult to know if similar results would be found for modifications to other psychotherapies. Efforts to reduce costs, improve access, or reduce dropouts have employed interventions that incorporate group therapy formats [596-601], self-help [602], digitally and remotely delivered interventions [603], in-home delivery [604, 605], and lay therapists [606]. While group TFP is considered generally less effective than one-on-one treatment, recent systematic reviews and RCTs indicate similar effectiveness as active controls [597, 598, 601, 607, 608], at least for exposure and cognitive-based group therapy. The following sections spotlight notable trends in psychotherapy optimization for PTSD.

##### 5.4.1.1 Modifying Exposure and Treatment Duration

Exposure interventions, the mainstay of treatment, deserve special mention, as they were developed based on the fear extinction model of PTSD and are the mainstay of current evidence-based treatment. Some research underscores the need for emotional engagement, activation and in-session extinction, while other authors emphasize between-session extinction and memory reconsolidation [609, 610]. Debate about the type, length, and necessity of focused traumatic memory exposure to PTSD recovery is ongoing. Some have reported concerns that exposure leads to emotional dysregulation and treatment dropout, or poor therapeutic relations, though there is inconsistency in this regard in the literature [287]. Prolonged Exposure, one of the earliest TFPs, emphasizes lengthy and repeated imaginal exposure and exposure to recordings of the trauma narrative. However, it appears that written narrative exposure may be a viable alternative to imaginal exposure [611, 612], with lower dropout rates [287]. An abbreviated written exposure protocol of 5 sessions has even shown non-inferiority to standard CPT Therapy [297, 599]. Surprisingly, briefer exposures lasting 10 to 20 minutes may be as effective as longer exposure periods [490, 592, 595, 613, 614]. Emerging therapies that utilize very brief exposures, or pendulation between exposure and disengagement from the memory, such as the Flash Technique [615], Somatic Experiencing [616, 617], Sensorimotor Psychotherapy, and 3MDR [618], and the positive impacts of non-trauma focused therapies [279], are further challenging the necessity of lengthy exposures. Evidence is emerging that bias towards or away from engagement with negative memories may contribute differently to frequency and controllability of re-experiencing symptoms [619]. Further, it has been argued that repeated efforts to stop memory retrieval when trauma reminders are encountered suppresses hippocampal and amygdala activity, engages the prefrontal cortex, and recruits extinction circuitry, leading to reduced intrusions, which are less emotionally intense, and improved emotion regulation [620]. This adds to memory reconsolidation science suggesting that surprise, or the addition of new contextual information (prediction error), is needed to update traumatic memories, wherein upon memory recall there is a mismatch between expectation and experience [206, 207] (also see EMDR 2.0 below). These complexities are highly relevant to PTSD treatment and remain to be fully explored.

##### 5.4.1.2 EMDR 2.0

EMDR 2.0 is a modification of EMDR, which emphasizes motivational techniques to reduce avoidance, optimal activation of the trauma memory network, and multiple, often simultaneous, dual attention tasks to vigorously tax working memory while the memory is desensitized [621]. The patient is asked to place the traumatic event into working memory in all its detail, with psychoeducation regarding the proposed working mechanism of the treatment. Activating the traumatic memory takes place by focusing on the sensory aspects to optimize the arousal level. Arousal may also be altered by, for instance, adding surprising comments or unexpected sounds or gestures. Different memory-taxing tasks are at the disposal of the therapist, in various sensory modalities (*e.g.*, visual, auditory, olfactory, gustatory), during a therapy session; some of these may use trauma reminders if enhancement of arousal is desired. The distractive task is often matched with the predominant sensory modality of the trauma memory, as there are some indications that this has a larger impact [622, 623]. EMDR 2.0 may also incorporate other imaginal or somatic elements, such as modifying posture or using movement. EMDR 2.0 also includes specific techniques to titrate the experience and counter dissociation or extreme avoidance.

##### 5.4.1.3 Combining Trauma-focused Psychotherapy Elements, or Adding Non-trauma-focused Elements

Combinations of trauma-focused, or trauma-focused and non-trauma-focused elements have been tried, especially for complex populations [624]. In essence, this offers “multimodal” therapy, with strategies for different facets of the illness, managing comorbidities, or illness consequences. For example, as mentioned previously, exposure and cognitive elements of TFPs have been combined, to good effect [592]. Integrated treatments for PTSD and SUDs, including Creating Change, Seeking Safety, and Concurrent Treatment of PTSD and Substance Use Disorders Using Prolonged Exposure (COPE) [380, 382, 625-627] are being evaluated, with benefit being reported for PTSD symptoms, SUD outcomes, [381], coping, self-efficacy, and quality of life [628], without deterioration of PTSD or SUD outcomes [378, 382].

TFPs combined with coping or emotion regulation skills are another theme, intended to mitigate distress and dropout rates in those with CPTSD, or PTSD and comorbid disorders such as BPD [366, 591, 624, 629, 630]. Dialectical Behavior Therapy (DBT), a non-trauma therapy for BPD that teaches people to challenge negative thoughts, regulate emotions, and adopt positive behavior patterns, has been combined with PE (*e.g.*, DBT-PE) [624] and, in DBT-PTSD, exposure, TF-CBT, Acceptance and Commitment Therapy (ACT) and compassion-focused therapy components [631]. DBT strategies have also been combined successfully with PE in Skills Training in Affective and Interpersonal Regulation followed by Exposure (STAIR-PE) [591]. DBT-PE has been found more effective than DBT alone for PTSD symptoms, including dissociation, while reducing dropouts, self-injury, and suicide attempts in those with BPD and comorbid PTSD [632]. DBT-PTSD, when compared to CPT, was more effective for reducing PTSD, BPD, and dissociative symptoms in female survivors of childhood abuse [633]. However, adding 8 sessions of emotion regulation skills training has not necessarily led to improved outcomes for PTSD, measures of emotion regulation, interpersonal problems, or dropouts, even when added to intensively scheduled EMDR or PE, which is associated with high emotional arousal [591, 629]. Other non-trauma-focused therapies, or their elements added to TFPs, studied for PTSD include music therapy, imagery, exercise, mind-body practices, creative or artistic elements, or cultural adaptations of therapies [634-644]. These may serve to customize therapy or increase engagement, including adapting therapy to personal, social, or cultural needs or preferences.

##### 5.4.1.4 Intensively Delivered Trauma-focused Psychotherapy

Intensively scheduled psychotherapy, once thought to be too much to be tolerated, is now an area of increasing study, with the aim to accelerate treatment and reduce dropout. At least as effective as weekly treatment, intensives reduce length of treatment without increasing adverse effects [591, 645-647]. Proposed mechanisms include reducing between-session distraction, avoidance, and demotivation that can theoretically result in loss of therapy engagement [648, 649], although this result is not consistent across studies [591, 592, 645]. One massed PE program measuring acceptability reported more positive reactions to intensive treatment (51.27%) than negative reactions (17.7%). Benefits reported by participants included fewer distractions and less avoidance, due to increased therapy structure, and quicker therapy gains, enhancing motivation and engagement. With respect to drawbacks, participants identified that massed PE caused short-term discomfort and created increased time and effort demands [650]. Recent systematic reviews reported that intensive or “massed” PTSD treatments are associated with large PTSD symptom improvements, and low dropout rates ranging from zero to 13.6%, with a pooled dropout rate of 5.51% across studies [651]. A similar review of PE studies also reported lower dropout for PE delivered at least twice weekly compared to less frequently (21% *vs.* 34%). Overall, these results indicate that intensively scheduled TFP leads to faster outcomes without sacrificing safety.

Some intensively administered psychotherapy programs combine elements from various therapeutic approaches, such as PE and EMDR. One such example is an 8-day program consisting of 3 hours per day of PE/EMDR, a psychoeducation group and 6 hours per day of physical activities to occupy time between sessions [649]. This program is also characterized by other adjustments intended to accelerate change: a) PE sessions prior to EMDR sessions, to activate the trauma memory network before processing the event with EMDR [590], b) therapist rotation, to reduce protocol drift [652], and c) EMDR 2.0 techniques, involving maximal working memory taxation with multiple dual attention tasks during desensitization (see also paragraph 5.4.1.2). Over time, this program has also developed specific protocols for case conceptualization and treatment planning, typically starting with the most distressing and intrusive Criteria A traumas, and techniques for handling dissociation and strong avoidance. Good results of the program have been reported in populations with severe CPTSD and high degrees of comorbidity, including those with BPD and suicidal ideation, with very low dropout [653]. Secondary analyses indicated that although those with more complex, dissociative symptoms had higher baseline symptoms, their treatment progressed at the same rate as those without such difficulties [364], and emotion regulation and BPD symptoms improved following treatment [306, 374]. More recently, this program was condensed into a 4 day program, with twice daily PE/EMDR (4 hours per day total therapy time), with similar reported results [649]. Intensive multimodal programs remain to be studied in randomized controlled trials.

#### 5.4.2 Emerging Psychotherapies for PTSD

##### 5.4.2.1 Emerging Trauma-focused Psychotherapies

Emerging TFPs introduced into clinical practice for the treatment of PTSD, with variable evidence base, include Imaginal Rehearsal Therapy (IRT), Accelerated Resolution Therapy (ART), Reconsolidation of Traumatic Memories (RTM), and somatic and body-oriented psychotherapies; this list is not exhaustive. ART, IRT, and RTM contain multiple components, building upon basic tenets of existing TFPs, and include rescripting interventions that allow the person to rewrite aspects of the experience [611]. For example, Accelerated Resolution Therapy (ART) is a manualized therapy that combines features of EMDR with imaginal rescripting of traumatic events, visual imagery and use of metaphors and Gestalt techniques [654]. It is highly protocolized, focuses on reducing somatic sensations and images associated with the traumatic event, and limits narration during therapy. This therapy is expanding in clinical practice and is supported by cohort studies and two small RCTs, in combat-related PTSD [655] and PTSD with complicated grief in hospice caregivers [656]. Dialogical Exposure Therapy, in contrast, also combines exposure with Gestalt theory, but it focuses on “self-processes” distorted by trauma. Its four phases include safety, stabilization, confrontation, and integration, with the major goal of restoring a sense of continuity of the self and agency to shape interactions in the environment. Confrontation not only includes trauma exposure but also “dialogical exposure”, similar to the empty chair technique in Gestalt, where the person has an interactional confrontation with the imagined presence of the experience (*e.g.*, a perpetrator, suicide or disaster) [657].

As mentioned, imaginal rescripting of memories is also a component of IRT and RTM. IRT is a CBT-based intervention, involving exposure to and rescripting of nightmares, with robust evidence for efficacy that rivals prazosin [658, 659]. This is important, as nightmares are linked to suicidality, and are an often ignored and treatment-resistant aspect of the illness [255]. RTM attempts to alter key aspects of the target memory (*e.g.*, color, clarity, speed, distance, perspective) to make it less impactful, and thereby reduce nightmares, flashbacks, and other features of PTSD. The memory is reviewed in the context of an imaginary movie theater, where the person imagines a fast (~45 sec) black-and-white movie of the trauma memory. The person makes adjustments as needed so that it can be viewed comfortably. This may promote emotional distancing from aspects of the memory [660]. Initial results are promising, primarily in military samples, as a brief, well-tolerated, cost-effective, manualized intervention for PTSD that is characterized primarily by intrusive symptoms, with positive follow-ups up to one year [660, 661].

##### 5.4.2.2 Somatic Trauma-focused Therapies

The landmark book The Body Keeps the Score, by van der Kolk and based on a paper in 1994, brought wide attention to mind-body consequences of traumatic stress, and how this inevitably impacts sense of self, human development, and relationships [662]. This book, which resonated with many clinicians and the public at large, introduced large audiences to conventional, lesser-known, and innovative trauma treatments, including mind-body interventions such as yoga, somatic psychotherapies, and neurofeedback, which will be discussed here and in subsequent sections. Somatic trauma-focused therapies, and their adaptations, have since become widely practiced clinically, ahead of an established evidence base. These psychotherapies differ from traditional TFPs in that they focus on bodily experiencing, heavily incorporating titrated, mindful attention to experiencing bodily states, including sensation, posture, motor urges, and other internal cues, with less attention to thoughts and cognitions. Somatic Experiencing [616, 617] and Sensorimotor Psychotherapy [663, 664] emphasize non-judgemental curiosity and pay specific attention to staying within the “Window of Tolerance” [665], meaning the level of nervous system activation wherein a person can maintain awareness of present experience, avoiding excess hypo or hyperarousal, or frank dissociation. Sensorimotor Psychotherapy which developed from Hakomi, another body centred psychotherapy, also incorporates aspects of psychodynamic, cognitive, and attachment-based therapies. While both Sensorimotor Psychotherapy and Somatic Experiencing include techniques aimed at working with and resolving trauma-associated arousal and automatic defensive responses (*i.e.*, action patterns arising from orienting, fight, flight, and freeze responses), Sensorimotor Psychotherapy has a stronger emphasis on healing developmental wounds. Emotional Freedom Technique is another somatic therapy that combines cognitive techniques with bodily tapping of various acupressure points [666-668]. While the evidence base is limited, they are based on evidence-informed practices such as mindfulness, exposure to sensorimotor experience, and neuroscientific findings in traumatology, with preliminary studies demonstrating potential. Given the need to address psychophysiological and somatic aspects of PTSD, and the growing popularity of these therapies, further scientific evaluation is needed.

##### 5.4.2.3 Emerging Non-trauma-focused Therapies for PTSD

In addition to Interpersonal Psychotherapy, Present Centred Therapy, and Stress Inoculation Training, which are currently recommended in some PTSD guidelines as evidence-based therapies, a number of other non-trauma-focused psychotherapies have been investigated for reducing PTSD symptoms. These include the adapted use of existing psychotherapies for PTSD, such as non-trauma-focused CBT [669], Acceptance and Commitment Therapy (ACT) [670-672], Metacognitive Therapy (MCT) [673], Dialectical Behavior Therapy (DBT) [633], and Behavioral Activation [674], with positive results compared to non-TFP controls. Many non-trauma-focused therapies contain common elements of “good therapy”, such as psychoeducation, skills acquisition, self-monitoring, or cognitive behavioral strategies, and may therefore foster safety and improved emotional regulation, and therefore less negative emotion, disrupted attention, rumination, and psychological inflexibility. Stand-alone, present-centered PTSD therapies that incorporate emotion regulation and interpersonal interventions are also included in this category, including Seeking Safety [368] and Trauma Affect Regulation: Guide for Education and Therapy (TARGET) [675]. The “Unified Protocol”, a transdiagnostic approach, is also being evaluated for PTSD [676, 677], given its utility for MDD and anxiety disorders, and because executive and emotional dysregulation are common across many diagnostic categories [677, 678]. The Unified Protocol consists of 5 core modules: a) mindful emotion awareness, b) cognitive flexibility, c) reducing emotion avoidance, d) tolerance of emotion-related physical sensations, and e) interoceptive and situational emotion exposures [677]. A small randomized controlled trial with 43 participants with posttraumatic psychopathology (PTSD, MDE, or an anxiety disorder) following severe injury reported large reductions in PTSD, anxiety, and depression symptoms, compared to usual care, which was maintained at 6 months [677]. Other non-trauma-focused therapies for PTSD, such as creative art therapies, including music therapy, art therapy, dance/movement therapy, drama therapy and expressive writing, have a weak evidence base [635, 637] at this time. While non-trauma-focused therapies generally have been found less effective than TFPs for PTSD symptoms [254, 283, 289, 368], they may still have a positive impact and may be important for those who are not ready for or do not want trauma-focused intervention. The sequence of interventions may also be important [369]; for example, CPT followed by BA has been reported to be superior to CPT alone, or the reverse sequence. How to individualize these components requires further research.

##### 5.4.2.4 Mind-body Interventions: Biofeedback, Mindfulness, Yoga and Acupuncture

With over a thousand studies, Heart Rate Variability Biofeedback (HRVB), a technology supported body-based intervention for self-regulation, has demonstrated efficacy for a broad range of physical, mood, anxiety and stress-related disorders, including reduction of both depression and trauma symptoms in PTSD [679]. Heart Rate Variability (HRV) is associated with the ability of the body to dynamically respond to the environment and return to homeostasis. Decreased HRV is a sign of vagus nerve and autonomic dysregulation, which is observed across psychiatric disorders, including PTSD. Impaired HRV is a pre-deployment predictor of post-deployment PTSD diagnoses and symptom severity in military-related PTSD [680], and a risk factor for cardiovascular disease and all-cause mortality. HRVB involves using a biofeedback device to train autonomic physiology. A HRV monitor displays physiological information on a screen and individuals use breathing and other techniques to alter autonomic responses. Despite its evidence base, it is rarely seen in psychiatric practice, despite the preponderance of physiological symptoms driving PTSD. HRVB is now available as mobile devices, allowing for home-based training and effects in four to 8 weeks.

Other mind-body interventions, such as mindfulness, meditation, and yoga are ubiquitous wellness interventions thought to help a range of stress-related conditions, through activation of the parasympathetic nervous system and reduced activation of the HPA axis, therefore reducing chronic stress-related inflammatory states, which are components of PTSD. Mindfulness-based approaches, such as Mindfulness-Based Stress Reduction (MBSR) and Mindfulness-Based Cognitive Therapy (MBCT), have shown efficacy for MDD and anxiety disorders and are thought potentially effective for PTSD because of the focus on non-judgemental thinking, self-compassion, attentional control, experiencing the moment, rather than avoiding it, and observing, rather than ruminating on thoughts. Trait mindfulness is associated with fewer PTSD symptoms [681], and mindfulness is thought to restore functioning of the SN, CEN, and DMN. Meditators have been found to have increased functional connectivity within the DMN, which is associated with self-referential processing and default responses (related to sense of “self”; see section 2.5). Increased DMN connectivity within the DMN, increased DMN connectivity with the CEN and reduced DMN connectivity with the insula have been shown to improve after mindfulness interventions in those with PTSD. For a detailed review, see Boyd *et al.* (2018) [240].

Similarly, yoga aims to enhance mental tranquility and control physiologic reactivity. Originating in Indian philosophy and practices for wellbeing, yoga has many types and forms. Western forms of yoga are derived from Hatha Yoga, and include physical postures, breathing techniques, and meditation [639], thus incorporating focused attention. The breathing component typically improves autonomic nervous system function and activation of the vagus nerve, improving mood and countering the hyperarousal typically encountered in PTSD. As in mindfulness, there is often a focus on interoceptive and proprioceptive awareness, which may slowly encourage tolerance of sensory experience and integration with higher-order executive systems [682]. Although quite distinct from mindfulness and yoga, acupuncture is thought to work through increased modulation of the vagus nerve, with downstream positive impact on the immune system. Furthermore, some authors have suggested that acupuncture might be viewed as a neuromodulatory treatment. It is associated with increased DMN functional connectivity and reduced amygdala activation. Also, acupuncture can modulate the periaqueductal grey, which has connection to limbic areas and pain pathways related to pain perception, memory, and dissociation, with potential implications for memory reconsolidation.

These mind-body interventions are promising for PTSD, as adjunctive interventions. Moderate to large effect sizes are reported for yoga, relaxation training, and mindfulness-based approaches for PTSD and comorbid anxiety and depressive symptoms [639, 640, 683, 684]. Yoga interventions vary considerably, but studies overall demonstrate a range of physical and psychological benefits, including for improving emotion dysregulation, HRV [685], and neuroimaging findings, such as increased gray matter volume in the insula and hippocampus, increased activation in the PFC, and altered connectivity in brain networks such as the DMN (See van Aalst *et al.* (2020) [686] for a review). However, a recent review of adapted Trauma-Sensitive Yoga interventions on PTSD and depression outcomes amongst women reported marginally significant to no effects [687]. Methodological issues make generalization difficult. However, because of its positive evidence base, acupuncture was given an emerging recommendation in the 2018 International Society for Traumatic Stress Studies PTSD guideline [254, 688], with variable treatment in other guidelines [254, 282] (See Assouline *et al.* (2022) for a review) [689].

##### 5.4.2.5 Spiritual and Moral Injury Interventions

A variety of psychotherapeutic interventions have been developed and investigated to address MI, shame, and/or spiritual consequences of trauma. Some therapies, such as CPT and PE, address distorted cognitions, have been adapted for use with MI, and are associated with reductions in trauma-related guilt and shame [690-694]. Research suggests that CPT may be a particularly well-suited intervention for trauma survivors who endorse self-blame [695]. While moral repair might be promoted by challenging trauma-specific cognitions [696] through CPT, CBT [697], or PE, some authors argue that such interventions may not be well-suited to situations of MI without PTSD [401, 698]. Mid-treatment trauma-related guilt and self-blame may predict trauma therapy outcomes in some populations [431]. There is literature to suggest that reductions in shame predict PTSD outcomes for CPT, but that CPT and exposure-based therapies are not impacted by baseline levels of self-blame [699] or guilt [322]. Better outcomes may be predicted by addressing avoidance and allowing general arousal during sessions, even in the absence of the emotional processing component of treatment [322]. Other authors, however, emphasize that CPT and PE are not traditionally focused on self-forgiveness and self-compassion [398, 700], which potentially reduce PTSD symptoms [321, 701]. Mindfulness-based interventions have also been highlighted and are associated with both elevations in self-compassion and reductions in self-criticism and PTSD symptoms [321]. The precise mechanism of these protective effects, however, is unknown.

Existential and spiritual aspects of therapy may play a critical role in recovery from MI. Spiritually-integrated interventions have been found to reduce PTSD, MI, and related symptoms [702]. One such intervention is Spiritually-oriented CPT (SOCPT) [703, 704], which was developed to validate guilt and shame related to violations of one’s core beliefs and address existential, spiritual, and religious challenges. Acceptance and Commitment Therapy (ACT), a “third wave” CBT focused on increased psychological flexibility, commitment to value-based action, as well as nonjudgmental acceptance of emotions and other internal experiences, has also been associated with reduced shame [705] and shows promise [404]. Further, spirituality and religious beliefs have been inversely associated with PTSD [706] and suicidal behavior [707], and are correlated with posttraumatic growth. Disclosure, empathy, choice, forgiveness, responsibility-taking, and amends-making, if appropriate, are often included in specific spiritual and MI interventions, such as Adaptive Disclosure (AD), Impact of Killing (IOK), and Building Spiritual Strength (BSS). Uncontrolled pilot studies of Adaptive Disclosure [708-710], which also includes forgiveness and reparative action [398, 426, 698, 709] demonstrated decreased PTSD symptoms, depression, and posttraumatic cognitions, and increased posttraumatic growth; other studies are underway [711, 712]. The Impact of Killing intervention for combat veterans, after focusing on thoughts and feelings related to killing, emphasizes self-forgiveness (*e.g.*, *via* letter writing) and amends-making [700, 713]. A pilot trial reported good acceptability, feasibility and improved PTSD symptoms, reduced psychiatric symptoms, improved functional outcomes, and higher rates of confiding personal thoughts and feelings to others. Building Spiritual Strength group therapy for those with military trauma-related religious and spiritual distress focuses on meaning-making [714]. Interventions promoting self-forgiveness and forgiveness of others may be more effective in addressing MI [389, 424, 691, 713, 715].

Numerous treatment modalities used by Chaplains have also been found to be helpful for MI. Chaplains in the US Veteran Affairs, for example, use pastoral counselling, meaning-making activities, forgiveness or repair activities, strategies to address emotional regulation, and spiritual practices [385, 405, 716]. Similarly, Canadian Armed Forces mental health Chaplains facilitate spiritual coping and grounding, reconciling worldviews, resolving anger at a God-figure (not specific to any perspective) and fostering reconciliation to facilitate recovery from MI [424, 717]. Additional spiritual practices that have been used to address MI include: prayer; meditation; spiritual/religious practices; spiritual guidance/direction; and the use of narratives, story-telling, spiritual writing, letter writing, and the writing practice of lament [405, 432, 718-721].

More MI-specific interventions for trauma-associated guilt and shame have also been developed. These include Trauma-informed Guilt Reduction therapy (TrIGR)[722] and Compassion-Focused Therapy [723], with promising initial results demonstrating decreases in guilt, depressive and PTSD symptoms [722], cognitive distortions, and suicidal ideation [723]. Additional interventions that yet require further research into their effectiveness in treating MI include Brief Eclectic Psychotherapy for Moral Trauma, Cognitive Therapy, Self-forgiveness: Addressing MI, Multi-Modal Motion-Assisted Memory Desensitization and Reconsolidation (3MDR), the Warriors Journey Retreat [724], and other novel approaches [725].

##### 5.4.2.6 Animal-assisted Therapy

Animal-assisted therapies, including equine-assisted therapy and canine therapy, have been embraced publicly as adjunctive treatment [726-729], including for children [730]. Veterans’ studies reported that service dogs reduced hypervigilance by alerting and creating boundaries, reducing nightmares, and improving sleep quality and duration. Dogs also helped veterans turn their attention away from invasive trauma-related thoughts and better regulate emotions [731, 732]. Additional reported benefits include improved emotional connections with others, increased community participation and physical activity, reduced suicidal impulses, and less medication use. Demands of training, adjustment to life with a service dog, and the delayed benefits may be challenging for many veterans and caregivers [728, 733]. Equine-assisted therapy, a new adjunctive therapy utilizing horses, often delivered as a 12-week program, is also considered preliminary but promising for PTSD symptoms, with improved treatment engagement and reduced drop outs [734, 735]. Interesting work indicates equine-assisted therapy has been associated with increased caudate functional connectivity and reduction in the gray matter density of the thalamus and the caudate; the increased caudate functional connectivity was positively associated with clinical improvement seen immediately at post-treatment and at 3-month follow-up. Authors suggested that equine-assisted therapy may target reward circuitry responsiveness and produce a caudate pruning effect from pre- to post-treatment [736]. Both biological and social effects may help address the challenges in engaging with and seeking appropriate assistance within social networks and care systems. While potentially beneficial, the evidence is currently weak for animal-assisted therapy for the purpose of treating PTSD [728, 734].

#### 5.4.3 Technology Supported Interventions

##### 5.4.3.1 Telehealth, Digital Treatment, and Related Technologies

Concerns about access to treatment, and the impact of the COVID-19 pandemic, have fostered an explosion of research regarding synchronous remote delivery of traditional TFPs, as well as development of digitally delivered therapies and hybrid interventions, with both synchronous and asynchronous components. Telepsychiatry [737] and remotely delivered psychotherapy by qualified therapists are likely to be equivalent to in-person therapy, although variability exists [738-741]. A variety of relevant reviews exist [603, 737, 742]. Treatment location and modality, and its symbolic meaning, may impact acceptability and engagement, depending on the home or provider environment and technology acceptability. This may impact outcomes. For example, in a military CPT study comparing remotely delivered or in-home treatment to office-based treatment, telehealth was the most acceptable and least often refused delivery format (17%), followed by in-office (29%), and in-home (54%) delivery [605]. Similar studies in other populations are needed, as setting acceptability may vary with a host of factors, such as age, personality factors, and access to technology.

Internet-delivered therapies, apps, and hybrid therapies require further evaluation. Digitally delivered cognitive behavioral interventions are most developed at the present time, although most are for depressive symptoms. Although many positive results exist, a review of fully internet-based cognitive and behavioral therapies, for example, showed poor quality evidence and sub-par outcomes [743]. Internet-based CBT and behavioral therapies may be associated with a clinically important reduction in PTSD post-treatment, compared to waitlist controls, but have not been found more effective at reducing PTSD diagnosis after treatment. This is not surprising, given the interpersonal nature of PTSD, in which people often feel isolated and need help in establishing a sense of safety. Further work is required to: establish non-inferiority to current first-line interventions, explore mechanisms of change, establish optimal levels of guidance, explore cost-effectiveness, measure adverse events, and determine predictors of efficacy and dropout [743]. There is also opportunity to leverage technology to allow remote monitoring of physiological parameters, language and other data that can also be used for machine learning algorithms to screen for, diagnose and identify treatment progress indicators in mental health disorders [744, 745].

##### 5.4.3.2 Attention and Cognitive Training

PTSD is associated with cognitive impairments, especially sustained attention and executive function, which predict development of PTSD and worsen because of it [746]. This bidirectional relationship is multifaceted and multifactorial, involving alterations of the SN and CEN, impacts of chronic stress, and contributions from comorbidities such as TBI, chronic pain, insomnia, MDD, and SUDs, and the impact of intense emotion on executive networks [746, 747]. Cognitive dysfunction drives poor outcomes, including role function, and is not addressed in clinical guidelines. Attentional Bias Variability is emerging as a potential neurocognitive marker of psychopathology, potentially mediated through emotional dysregulation [748], especially for persons with trauma, where it is related to the degree of trauma symptoms [749].

Computerized and non-computerized adjunctive interventions aimed at secondary prevention [750] and cognitive rehabilitation of PTSD are in development to address the impacts of such deficits, including for military populations, where TBIs are common and military training emphasizes ongoing vigilance to threat [751, 752]. Attention Control Training, for instance, normalizes attentional function by targeting fluctuations of attention towards and away from threats, which may underlie hypervigilance, avoidance, and dissociation [750]. Attention Control Training uses a computerized program to briefly display two stimuli, one threatening and one neutral, followed by a probe in the location just above one of the two stimuli. The program measures attentional bias in a person, based on reaction times to the probe, and trains the person to respond more equally to both neutral and threatening cues. Attention Control Therapy developed from a control condition in studies of Attentional Bias Modification, which helps anxious patients to shift attention away from threat. However, it appears at least as, and possibly more effective than Attentional Bias Modification in PTSD [750]. There is a need to personalize such interventions, given the variation of what is threatening to individuals, contextual variables (*e.g.*, re-entering civilian life after leaving military service), whether attentional biases are automatic or conscious [753], and the impact of threat sensitization and accumulating traumas over time. Further, suggestions that PTSD-related attentional biases are relatively selective for emotional stimuli raise the possibility of incorporating affect labeling training [201, 754].

##### 5.4.3.3 Virtual and Augmented Reality

Virtual reality (VR) enhanced therapies combine technology with exposure techniques to increase emotional engagement and counter avoidance that often hampers exposure treatment, often by using multi-sensory trauma-related cues to ensure activation of the trauma memory network [755]. Precursors to VR go back to the earliest days of PTSD therapy post-WWII, when exposure was augmented by playing short movies or sound clips of traumatic reminders such as battle scenes [756]. Technological advances have allowed computer-simulated imagery, addition of sensory elements (*e.g.*, vibration, olfactory cues) [757, 758], and concomitant physiologic monitoring, which has demonstrated reduced physiological hyperarousal following exposure treatment [293, 294]. Augmented Reality Exposure Therapy (ARET) adds digital stimuli, including trauma cues, to the physical world, which may increase emotional engagement [755]. In other words, the person can be present in the real world at the same time as being exposed to fear-related stimuli. Virtual Reality Exposure Therapy (VRET), on the other hand, is more immersive, in that the person is drawn into their digital surroundings, either in the form of a VR headset or large display screens (see Eshuis * et al.,* 2021 for a review [759]). Preliminary studies on VRET are promising, including for treatment-resistant PTSD, but there is a dearth of clinical trials of ARET. VR studies have been performed in civilian and military populations, including pilot trials for interpersonal trauma, such as MST [760]. Newer advances in VR include the use of avatars [761] and remote therapy [741].

##### 5.4.3.4 Multi-Modal Motion-Assisted Memory Desensitization and Reconsolidation (3MDR)

Multi-Modal Motion-Assisted Memory Desensitization and Reconsolidation (3MDR) offers another novel approach to the treatment of PTSD and MI. An exposure-based psychotherapeutic intervention, 3MDR incorporates treadmill walking within a personalized, multi-sensory, immersive virtual reality environment and dual attention tasks from EMDR [762]. 3MDR combines and personalizes therapeutic approaches to improve therapy engagement, break through persistent cognitive avoidance, optimize arousal, engage with trauma memory networks, and leverage memory reconsolidation science [763, 764].

3MDR involves preparatory, treatment, and reconsolidation sessions. During preparatory sessions, patients identify images and music related to traumatic experiences. During each 90-minute treatment session, the participant continually walks on a treadmill for 60 minutes within the VR environment, with the therapist “walking alongside”. During seven successive 3- to 5-minute cycles per session, the participant describes each image. Arising associations, physical sensations, and feelings are identified, projected onto the image, and repeated aloud by the participant. A brief EMDR dual attention task follows. After the seventh cycle, the participant cools down while music is played to help with grounding in the present. A period of debriefing and reconsolidation concludes the session [762].

Initial 3MDR results, including from three randomized controlled trials, are promising, with significant improvement in PTSD, depression, and anxiety symptoms, including in military populations with treatment-resistant PTSD [618, 762, 765-767]. Dropout rates are notably low. Large effect sizes for PTSD are reported, with further improvement up to three months follow-up. Initially high heart rate, respiratory rate, and distress scores reduced over therapy, indicating high emotional engagement during therapy [768]. Cortisol elevations have also been associated with 3MDR response, which may be key to successful treatment outcomes [618, 765]. Improvement in MI symptoms, emotional regulation [769], resilience [762], function, well-being and relationships, sustained up to 6 months post-intervention, have also been reported Findings align with studies combining exposure with exercise to promote extinction retention (possibly *via* increased release of BDNF) [770, 771], showing that exercise both improves PTSD, depressive and anxiety symptoms [772, 773] and increases divergent thinking [764]. Ongoing studies in the Netherlands, United Kingdom, United States, and Canada aim to further explore the underlying mechanisms of 3MDR and its application with various populations.

### 5.5 Medication-augmented Psychotherapy

#### 5.5.1 Introduction

Interest in substance-assisted psychotherapy, including medications and psychedelics, has been particularly robust; interventions investigated to enhance TFP or exposure tasks include cortisol and corticosteroids [774], D-cycloserine (DCS), methylene blue, oxytocin, and yohimbine [775-777]. The earliest substances used to enhance therapy were barbiturates, used to facilitate emotional processing during therapy after WWII [756]. Rationale for pharmacologically augmented psychotherapy includes reducing sympathetic overactivity, facilitating fear extinction or memory reconsolidation processes, improving access to and confrontation of traumatic memories and promoting catharsis [756]. More recent mechanisms touted to be involved in MDMA and psychedelic-assisted psychotherapy (discussed in Section 5.6 Psychedelic-assisted Psychotherapy) include enhancing trust and therapeutic alliance, therapeutic dissociation (allowing access to a third person perspective or specific ego states), enhancing neuroplasticity, altering brain connectivity, and shifting identity [778]. This section will focus on DCS, hydrocortisone, oxytocin, morphine and propranolol, which have been investigated the most in this area, before discussing psychedelic-assisted psychotherapy.

#### 5.5.2 D-cycloserine Augmented Psychotherapy

One of the early pharmacological attempts to enhance TFP was DCS, an antibiotic that acts as an agonist of NMDA receptors and promotes extinction learning in animals. Blocking the NMDA receptor impairs extinction retention whereas enhancing the functioning of the NMDA receptor facilitates fear extinction [779, 780]. However, NMDA receptor subtypes are differentially distributed throughout the central nervous system and play roles in both the formation and reinforcement of fear responses as well as fear extinction [781, 782]. The glutamate (NMDA) ionotropic receptor subunit 2A (GluN2A) may be involved in the initial formation and stabilization of the fear response, whereas the GluN2B and GluN2C receptors are both involved in extinction of fear responses. D-cycloserine has been found to enhance fear extinction during exposure tasks in both animals and humans. Preclinical evidence also suggests that NMDA NR2B receptors are involved in memory reconsolidation after traumatic memory recall, and therefore DCS may increase labilization of old traumatic memories [783]. D-cycloserine as an adjunctive PTSD treatment without psychotherapy yielded disappointing results [784], but it was hoped that by enhancing NMDA activity, the impact of psychotherapy on fear extinction could be enhanced [785] and increased neuroplasticity would facilitate long-term therapy gains. Initial results were promising, but overall, results have been mixed, with effects ranging from faster symptom resolution, no impact, and even detrimental effects [784, 786]. One exception may be PE: DCS administered before PE has been found to significantly lower PTSD symptom scores and modestly lower the dropout rate, compared to PE alone [787]. NMDA receptors and their interactions are therefore complex and require co-agonists glycine and glutamate to be fully activated. Work in this area is ongoing with other NMDA active agents being tested, including ketamine, which is an NMDA antagonist rather than an agonist (as discussed in 5.3.3 and 5.6.2) [784]. Preclinical work combining DCS with midazolam has also been explored, with DCS reactivating long-term fear memory so that its reconsolidation can be blocked with midazolam [788]. These interventions may be combined with strategies to increase prediction error upon recall, to further optimize impact on memory reconsolidation. Again, context matters, as may the order and timing of multi-component interventions.

#### 5.5.3 Hydrocortisone Augmented Psychotherapy

Hydrocortisone-augmented exposure therapy was developed based on preclinical and clinical studies finding that corticosteroids may promote the extinction of fear [789-791]. RCT evidence in combat-related PTSD demonstrates that participants given intravenous hydrocortisone and asked to describe their trauma exhibited more improvement in PTSD-related symptoms one week later, but not at the one month follow-up [790]. These authors repeated the study with 54 veterans and multiple dexamethasone administrations (four times weekly), with a trauma memory reactivation task. There was a greater, but nondurable, reduction in PTSD symptoms at one and three months [776]. In another RCT, oral hydrocortisone 30 mg given prior to PE sessions three through ten improved PTSD symptom reduction and dropout rate compared to placebo-augmented psychotherapy [792]. Although data are limited, hydrocortisone-augmented psychotherapy may be promising [500].

#### 5.5.4 Oxytocin Augmented Psychotherapy

Oxytocin augmentation of psychotherapy is theoretically useful, given oxytocin’s prosocial and anxiolytic effects, and therefore potential for improved therapeutic alliance and willingness to engage in trauma treatment. Oxytocin is associated with altered emotional regulation processes *via* modulation of functional coupling between the PFC and amygdala, thereby impacting fear extinction. In healthy participants, the oxytocin-treated group showed heightened facilitated fear extinction [793]. In female PTSD patients, intranasal oxytocin (24 IU) taken 50 minutes prior to a trauma script challenge was related to reduced provoked total PTSD symptoms, compared with control [794]. However, more recent trials have been disappointing, including a double-blinded RCT of oxytocin-augmented PE [500, 795].

#### 5.5.5 Propranolol Augmented Psychotherapy

Propranolol, a beta-adrenergic antagonist, has been investigated not only for adjunctive PTSD treatment but also for augmenting psychotherapy for PTSD. Propranolol is associated with reduced PTSD symptoms within experimental brief memory reactivation paradigms [796], consistent with the memory reconsolidation theory that retrieval of memory under certain conditions leads to its labilization and subsequent re-storage, which could be disrupted by drugs [205]. A metaanalysis of propranolol interventions for disrupting trauma memory for PTSD analyzed seven studies reporting on PTSD symptom change and three studies on the effects of propranolol on physiological responses. Overall, results indicated that propranolol did not show a beneficial effect on PTSD symptoms, skin conductance, or electromyography response, but did reduce heart rate after trauma memory recall compared to placebo. However, authors cited heterogeneity, variation in propranolol dosage, and inadequate sample sizes as limitations to current evidence [451]. Given emerging evidence on the complexity of memory reconsolidation processes, such as the importance of prediction error, these may need to be considered in future studies.

### 5.6 Psychedelic-assisted Psychotherapy

Psychedelic-assisted psychotherapy is perhaps the most novel and publicized of potential PTSD interventions, although it has a long history, with roots in ritual and religious contexts for millennia across multiple cultures. Cut short by criminalization in the 1980s, clinical exploration of their medical use dates to the 1950s. Positive findings for MDMA-assisted psychotherapy for PTSD, and psilocybin for treatment-resistant MDD, sparked renewed interest. Again, technological and conceptual advances in PTSD and brain research make it possible to begin to understand the potential power of combining these compounds with psychotherapy, and their multi-modal effects throughout the brain. These medicines, in a broad sense, include traditional psychedelics, such as 2A serotonin receptor (5-HT2A) agonists lysergic acid diethylamide (LSD) [797], psilocybin found in “magic mushrooms”, and N,N-dimethyltryptamine (DMT), the active ingredient in ayahuasca, entactogens like MDMA, and other substances like ketamine, with hallucinogenic or empathogenic properties [798-800].

Multiple potentially transdiagnostic mechanisms are proposed, across several levels of analysis, from pharmacological to cultural, which have been reviewed elsewhere [801]. Psychedelics impact a broad range of neurotransmitter systems, act as neuroplastic, immunomodulatory, and anti-inflammatory compounds, and impact brain circuits involved in introspection, self-referential thought, constraints on thinking, and a person’s sense of trust, connection and compassion with self and others [799, 801, 802]. Specific to PTSD, psychedelics may reduce amygdala activity, alter functional brain connectivity, disrupt the DMN, and facilitate neuroplasticity through increased production of BDNF and anti-inflammatory processes [778, 801]. Further, these compounds induce an altered state of consciousness that allows increased self-awareness and experiencing of emotion and sensation, and confrontation with previously avoided and unconscious material. Therapeutic dissociation may occur, especially for ketamine, which allows greater ability for perspective taking and feeling of long unexpressed emotions, often leading to new meaning-making. Mystical experiences may occur, depending on the substance and dose, which are associated with self-compassion and positive clinical outcomes [803-807]. It has been hoped, and initial results indicate, that these unique “mind-manifesting” substances may therefore be useful to target the avoidance, generalized sense of threat, moral injury, rigid trauma and shame-based cognitions, disconnection, and associated alterations in self-identity often complicating PTSD. While one experience may not cure PTSD, these often transformative experiences can, when paired with psychotherapy, open the door to recovery and posttraumatic growth [806, 807]. Current interest for PTSD is focused on MDMA, ketamine, LSD, and psilocybin, which will be discussed in the following sections (see also Krediet * et al.,* 2020 [778]). Research on novel compounds based on modifications to these agents, created to improve tolerability, optimize duration of action and enhance efficacy, is forthcoming [808]. However, what must be emphasized is that all this happens in the context of a relationship, which can either enhance or sabotage these effects. Set and setting (the context of the therapy, environment, intention, and therapeutic relationship) remain integral to outcomes.

#### 5.6.1 MDMA-assisted Psychotherapy

Methylenedioxy-methylamphetamine (MDMA) assisted psychotherapy (MAP) is currently the most supported psychedelic-assisted psychotherapy paradigm in PTSD [809]. Technically not a classical psychedelic, MDMA is an entactogen or “empathogen”, which increases a person’s sense of connection and trust with others, with mild psychedelic properties [810, 811]. However, it is often subsumed under the umbrella of psychedelic-assisted psychotherapies, given its ability to alter consciousness.

Initially used to facilitate therapy in the 1970s until it was made illicit in the 1980s, MDMA-enhanced psychotherapy research was resurrected by the work of the Multidisciplinary Association for Psychedelic Studies (MAPS) [802]. MAPS designed a treatment protocol emphasizing an inner-directed approach that also includes strategies from transpersonal, somatic and Internal Family Systems therapies, and which views each individual as having a natural tendency to move towards healing, given facilitative conditions [812]. MAPS has conducted phase 2 and 3 clinical trials [813, 814], leading to the FDA designation for MDMA as a breakthrough therapy for PTSD. The MAP treatment involves three phases: preparation sessions, experiential medicine sessions, and integration. Preparation includes rapport building, assessment, creating a sense of safety, psychoeducation, and intention setting. Two to three experiential sessions using MDMA follow, supported by the therapist. MDMA dose ranges from low dose (25 mg to 40 mg) to full dose (100 mg to 125 mg) [813-817]. During MDMA sessions, the therapist follows an “inner-directed” model whereby participants focus on noticing and allowing their inner experience to unfold, often wearing eye shades and listening to music, with periods of interaction with the therapist. Integration sessions follow each MDMA session, during which the therapist and patient explore the MDMA session, its meanings, and how to incorporate insights into daily life [812]. One exception to this model is an open pilot study utilizing MDMA-facilitated Cognitive-Behavioral Conjoint Therapy with six couples in which one partner had PTSD. This included a 15-session Cognitive-Behavioral Conjoint Therapy protocol and two MDMA sessions, with both partners participating [818].

Typical effects of MDMA, when combined with psychotherapy, include increased ability to access and process painful or negative emotions, increased trust and empathy, increased sense of connection to self and others, and improved range of positive emotions [811, 819]. Biochemically, MDMA increases release of serotonin, dopamine, noradrenaline, oxytocin, cortisol, prolactin, and vasopressin [820-822], which influence cognition, mood and perception. Receptor functions include agonism at 5HT1A and 5HT2A receptors, and blockade of the SERT, NET, DAT and VMAT2 transporters [205, 799]. Noradrenaline and cortisol may enhance arousal needed for improved fear extinction learning, while reduced amygdala activation and increased vmPFC activity may allow the difficult experiences to be navigated in a tolerable manner [822, 823]. These effects also increase cognitive flexibility [811] and positive responses to emotions, and improve social interactions [824, 825]. Oxytocin release may be involved in increased self-compassion, interpersonal connectedness, empathy and sense of increased intimacy in the therapy room [778, 799, 811]. It is theorized that the positive state induced by MDMA during trauma processing could result in a mismatch between the positive emotions experienced during MAP and the expectation of intolerable negative emotions upon recall of traumatic memory. There may also be a buffering of shame, as MDMA facilitates self-compassion and self-connection, breaking down another barrier to reviewing past traumas. These experiences may therefore allow the memory to “update” upon reconsolidation [206, 826], leading to fear extinction and reduced PTSD symptoms [827]. Posttraumatic growth, consisting of positive changes in self-perception, interpersonal relationships, or philosophy of life, may follow [828]. Interestingly, MDMA-associated increases in trust, but not empathy, may be impacted by common genetic variants of the oxytocin receptor gene [829], indicating interindividual variables yet to be discovered.

Current literature supporting MAP as a safe, well-tolerated, and efficacious treatment for PTSD, including treatment-resistant illness, includes open-label trials, six small phase 2 trials, and one phase 3 randomized controlled trial, spanning both civilian and military populations. Systematic reviews [542, 830-832] report a high rate of clinical response and remission and large effect size for reducing the symptoms of PTSD and depression. Improvements in Sheehan Disability Scale [813] and sleep quality [833] have also been reported. Study quality has been reported as moderate [542, 830]. A dose-response has been demonstrated, as well as high response rates in “placebo” groups undergoing high-quality psychotherapy without MDMA [815, 816, 834]. Adverse events reported include low mood, nausea and jaw-clenching during sessions, and lack of appetite [832]. Importantly, no major drug-related adverse effects or neurocognitive effects have been reported and there are reports of durable improvements for up to six years [835, 836]. Overall, systematic reviews of observational studies and RCTs [542, 834] indicate support for MAP for PTSD, with further larger trials needed to confirm these findings and to investigate the impact of pairing other psychotherapeutic interventions.

#### 5.6.2 Ketamine-assisted Psychotherapy

While most of the data on ketamine as a PTSD treatment is as a stand-alone pharmacologic intervention, some evidence indicates ketamine-assisted therapy may also be beneficial. The glutamatergic system [837] is implicated in the formation of traumatic memory, mediation of the stress response, and pathophysiology of PTSD [543, 546, 838], as previously discussed. Ketamine may also reverse stress-induced reductions in synaptic density and complexity in the PFC and hippocampus in animals [839]. A single low dose of ketamine, administered upon fear retrieval in monkeys, reduced contextual fear memory and attenuated neurogenesis in the hippocampus [840]. These are important findings for considering ketamine as a potential candidate to target traumatic memories in PTSD. Like psychedelics, ketamine is associated with alterations in the DMN and improved depressive symptoms, with additional antisuicidal effects [841]. Ketamine-assisted psychotherapy may also take advantage of prominent dissociative properties. With low doses, there can be a sense of separation from bodily processes, with increased ability to observe mental experiences without rigid defenses. Higher doses are associated with much greater separation from usual mental states and awareness of “here and now’ reality. There can be a greater sense of disembodiment, and more focus on internal experience, often with mystical or archetypal themes [778, 807, 842]. These experiences can be explored with the therapist during subsequent integration sessions.

Early work using ketamine-assisted psychotherapy for PTSD is promising. A feasibility study using three weekly intravenous ketamine (0.5 mg/kg) sessions to augment a 10-week PE intervention in veterans with PTSD was positive [547]. While only 10 participants completed the trial, both PTSD and depressive symptoms decreased from baseline to end of treatment. After controlling for depression scores, differences in total PTSD symptoms and avoidance subscales remained significant [547]. Studies comparing ketamine only versus ketamine-assisted psychotherapy are needed.

#### 5.6.3 LSD, Psilocybin and Other Psychedelics

Classical psychedelics, such as lysergic acid diethylamide (LSD), psilocybin (4-phosphorloxy-N,N-dimethyltryptamine), and N,N-dimethyltryptamine (DMT) [799, 801, 843], are famous for causing psycho-sensory changes such as visual hallucinations, which are thought to occur through 5-HT2A agonism in visual and association cortices. However, these compounds have a wide variety of cognitive, mood, and perceptual effects explained not only by 5-HT2 agonism, but also by their impact on other serotonergic, dopaminergic, and other receptors, which may impact reward and stress sensitivity [801], and downstream effects to increase glutamate in the PFC and neuroplasticity *via* increased BDNF [801, 844, 845]. Psilocybin and LSD are both agonists at 5HT1A and 5HT2C receptors. LSD and 2,5-dimethoxy-4-iodoamphetamine (DOI), another classical psychedelic, also increase oxytocin release, associated with increased empathy, connectedness and sociability. Both ayahuasca, which contains DMT, and LSD have agonist activity at D1 receptors, whereas LSD has broader effects on D2 and D4 receptors. Ayahuasca also has agonist effects at trace amine-associated receptor (TAAR1) and sigma (𝜎1) receptors. Some authors have suggested that classical psychedelics disrupt networks involved in cortical control, increasing functional connections between brain areas not usually connected, and releasing the usual inhibitory feedback loops preventing sensory, interoceptive and other information from overloading awareness, leading to alterations in perception of reality. These experiences may include mystical or life-changing transcendent experiences [806], even ego dissolution, where boundaries of self and others are blurred and there may be a sense of unity [843, 846-848]. As such, when combined with accessing traumatic experiences, these aspects may be incorporated into the traumatic memory network, so that it is stored with new emotional significance [849].

Historical, preclinical and preliminary human data provide support for further investigating ayahuasca, LSD and psilocybin for PTSD. LSD was used in the Netherlands by psychiatrist Jan Bastiaans for survivors of the KZ syndrome [850, 851]. A famous case of LSD treatment was described by Yehiel de Nur, a survivor of Auschwitz, who overcame his survivor guilt with this method [852]. Ayahuasca, used for millennia in ceremonial spiritual medicine practices in the Amazon, has been found to have antidepressant effects, to enhance fear extinction [853, 854], and to reduce inner reactivity [855]. It is associated with markers of mindfulness and psychological flexibility [855], such as creative divergent thinking [856], and increased introspection [857]. Surveys of Special Operations Forces Veterans report reduced retrospective self-reports of suicidal ideation, and symptoms of PTSD and anxiety [805, 858].

LSD, used in psychotherapy since the 1950s [802], has an important history but no randomized controlled trials exist for PTSD, likely partly related to the late establishment of the current PTSD diagnosis in 1980 and changing evidentiary standards. Preclinical and in vivo data suggest LSD is associated with increased neuroplasticity, increased plasma BDNF, reduced amygdala reactivity, enhanced fear extinction, altered sensorimotor gating, empathogenic effects, reduced anxiety, and increased quality of life [859-861].

While LSD has a longer history of clinical use, current interest for PTSD is strongest for psilocybin. While no published trials of psilocybin for PTSD currently exist, trials are now underway. Interest in psilocybin, and its active metabolite psilocin, was sparked by research indicating positive results in end-of-life distress and treatment-resistant MDD [810]. Also, psilocybin’s psychedelic effects, and enhanced ability for introspection, typically last 3 to 6 hours, which is shorter than LSD and better suited to clinical use. Like other psychedelics, psilocybin is associated with reduced amygdala reactivity during emotion processing [542, 862]. Psilocybin interrupts habitual negative patterns of thinking and allows for creative, dialectical thinking where multiple perspectives can be considered. Further, psilocybin has been associated with greater connection to both emotional and somatic sense perception, perhaps allowing more integrated experiential processing of avoided or dissociated material, often with acceptance, self-compassion [863], and emotional empathy [864] rarely found in chronic PTSD. This is thought to emerge from psilocybin’s potential to alter default mode networks [865, 866], and enhance connectivity between a plethora of brain areas, including somatosensory areas [867], while also enhancing serotonergic and dopaminergic transmission, which may help modulate negative emotion [778, 799, 801]. In addition to facilitating fear extinction, psilocybin promises to provide a deeper psychological shift, addressing the hardened trauma-based cognitive distortions that disturb identity and disconnect sufferers from themselves and the world. This may, theoretically, be useful in the case of moral injury. Further, like ketamine, psilocybin has been shown to jump-start neuroplasticity [844], potentially enhancing memory extinction *via* increased hippocampal neurogenesis [868], reversing stress-induced prefrontal changes [869], and providing a window for enhancing insights emerging from psychotherapy, to be consolidated through long-term potentiation.

The following section will address other interventions impacting neurological mechanisms: neuromodulation and nerve blockade.

### 5.7 Development in Targeting the Brain and Nerves: Neuromodulation and Nerve Blockade

Neuromodulation, in the form of Electroconvulsive Therapy (ECT), was one of the first effective somatic psychiatric treatments. The use of invasive and non-invasive neuromodulation for the treatment of neurological and psychiatric disorders has grown exponentially in recent years. Advances in understanding altered neurocircuitry in PTSD, such as altered activation patterns in the PFC, anterior cingulate cortex, and limbic system, has led to potential targets for neuromodulation technologies. These technologies include Deep Brain Stimulation (DBS), seizure induction therapies such as ECT and Low Amplitude Seizure Therapy (LAP-ST), repetitive transcranial magnetic stimulation (rTMS), transcranial direct current stimulation (tDCS), and other treatments such as neurofeedback, vagus nerve stimulation, and stellate ganglion blockade.

#### 5.7.1 Deep Brain Stimulation and Electroconvulsive Therapy

Deep Brain Stimulation (DBS) has theoretical support from animal models and a few case reports, with electrode placement in the amygdala, or in the mPFC and uncinate fasciculus [870]. However, it requires neurosurgical placement of an electrical pulse generator directly into the brain, which limits its use and recruitment into clinical trials. ECT, on the other hand, has a long history and remains an important tool in psychiatry, usually utilized for severe depression. It is associated with increased neurotransmitter release and neuroplasticity, mediated through upregulation of BDNF. One small prospective RCT and an open study support its use in treatment-resistant PTSD [871], in addition to two retrospective studies in comorbid PTSD and MDD [872]. However, its use is limited by cognitive side effects, cost, general anesthesia risk, access and stigma. Low Amplitude Seizure Therapy (LAP-ST) uses markedly lower amplitude electrical pulses to induce seizures, which limits the depth of the current to cortical regions such as the PFC, and therefore reduces cognitive side effects associated with involvement of the hippocampus. A small, proof-of-concept trial of LAP-ST compared with right unilateral ECT has been published, showing promise [873].

Non-invasive technologies are of particular interest for PTSD, including repetitive Transcranial Magnetic Stimulation (rTMS) and transcranial Direct Current Stimulation (tDCS), given the lower risk of adverse effects. Currently, the literature for rTMS and tDCS are most developed, which will be reviewed in the following sections.

#### 5.7.2 Repetitive Transcranial Magnetic Stimulation (rTMS)

Repetitive transcranial magnetic stimulation (rTMS) is a well-tolerated, non-invasive brain stimulation therapy, often used for MDD. rTMS involves briefly passing a magnetic field through the scalp and skull, at specific locations, which alters underlying cortical and subcortical activity in specific brain networks. High frequency (HF) stimulation (>5 Hz) generally increases cortical excitability, or stimulates a brain area, while low frequency (LF) stimulation (≤1 Hz) decreases cortical excitability [874].

Multiple systematic reviews of rTMS have indicated positive results, with large effect sizes, for the reduction of core PTSD and depressive symptoms, including for augmentation treatment of military-related PTSD. However, small sample sizes, methodological heterogeneity and gaps regarding optimal dose, brain location, and long-term outcomes indicate the need for further study [875-878]. Both right dlPFC, left dlPFC, and other targets appear equally effective [876, 877], although recent analyses indicate a higher effect size for HF rTMS, compared to LF TMS, for both PTSD and depressive symptoms. Greater number of sessions (greater than 19) and larger treatment doses were not associated with stronger treatment effects and may be counterproductive [876]. Few studies test durability of effect past two to four weeks [877]. However, one study tested long-term effects of Intermittent Theta Burst Stimulation (iTBS) for PTSD, finding clinically meaningful improvement of PTSD symptoms up to a year after treatment [879]. rTMS has also been used to enhance TFPs and other nonpharmacological interventions. For example, rTMS focusing on the ventromedial and dorsolateral PFC is thought relevant for extinction learning and has been used in small pilot trials to augment CPT [880] or exposure-based interventions [881-883]. Results are too preliminary to draw conclusions, although rTMS given just prior to CPT in combat veterans *via* right dlPFC stimulation (110% MT, 1Hz continuously for 30 minutes, 1800 pulses/treatment) reportedly led to improved PTSD symptom reductions that were sustained for up to six months; this study had high dropout [880].

The success of heterogeneous rTMS protocols can be explained by the broad aspects of PTSD being targeted. Previous data show activation of the right hemisphere during anxious arousal and PTSD symptoms during the processing of trauma-specific information [877], in keeping with the superiority of right prefrontal rTMS to reduce anxiety and PTSD symptoms [878]. That both right and left rTMS, and both HF and LF stimulations, are effective is explained by the possibility that different rTMS protocols are targeting different symptoms or neurological mechanisms underlying PTSD. Right dlPFC stimulation has been associated with greater reductions in core PTSD symptoms [878, 884], possibly by exciting the HPA and therefore reducing amygdala activation [885], or by inhibiting the corpus callosum [886], while left dlPFC stimulation tends to improve mood symptoms [884]. Changes within and between networks such as the DMN, SN, and CEN may be involved [876, 877], and raise exciting possibilities for individualization of treatment. For example, some authors have suggested that the relative severity of symptoms in patients with comorbid PTSD and MDD might determine the decision for applying a left or right stimulation protocol [877].

#### 5.7.3 Transcranial Direct Current Stimulation (tDCS)

Transcranial direct current stimulation (tDCS) for the treatment of PTSD is in its infancy. tDCS leads to sub-threshold shifts of resting membrane potentials by applying direct currents, *via* scalp electrodes, over targeted cortical areas [887]. Anodal tDCS increases the excitability of the cortex whereas cathodal tDCS decreases it. Its low cost, ease of use, portability, and good safety profile create practical advantages over other neuromodulation technologies. One potential target involves increasing the inhibitory control of amygdala activity [877]. Positive studies in anxiety disorders and depression, as well as two PTSD studies, with stimulation of the dlPFC, indicate potential for PTSD [877, 888]. One study used tDCS in combination with working memory training and produced improvements in performance [889]; another involved a fear conditioning paradigm and showed tDCS facilitated consolidation of extinction memory [889]. A pilot trial of veterans receiving virtual reality exposure with or without tDCS was associated with greater reductions in physiological arousal [890].

#### 5.7.4 Vagal, Trigeminal and Auditory Nerve Stimulation

In keeping with the allostatic load model of PTSD, technologies such as non-invasive transcutaneous cervical vagus stimulation (tcVNS), trigeminal nerve stimulation (TNS), and acoustic nerve stimulation [680, 872] target autonomic nervous system dysregulation thought to evolve from chronic stress. The autonomic nervous system allows bidirectional communication and coordination between the brain and peripheral physiology, and altering its function can have broad systemic effects [680]. Trauma-related sympathetic hyperarousal is thought to involve maladaptive right temporal lobe activity, whereas the avoidant and dissociative features have been conceptualized as dorsovagal parasympathetic “freeze” responses driven by the left temporal lobe [680]. Vagus nerve stimulation, which has been demonstrated to be effective in depression, stimulates ascending fibres in the vagus nerve. Electrical stimulation travels to the nucleus tractus solitarius in the brainstem, which is interconnected with the thalamus, hypothalamus, amygdala, locus coeruleus, raphe nuclei, reticular activating system, limbic forebrain, ACC and insula, with downstream activation of the medial temporal lobe and PFC. VNS electrodes may be surgically implanted and wrapped around the cervical vagus nerve or applied transcutaneously to the auricular branch of the vagus at the ear [872]. A pulse generator delivers current, similar to DBS, which has been reported to reduce sympathetic reactivity [891], facilitate extinction and prevent the reinstatement of conditioned fear in animal models [892]. Trigeminal Nerve Stimulation (TNS) also uses cutaneous stimulation of afferent cranial nerve fibres, which project to the brainstem, limbic system, and cortex, also through the nucleus tractus solitarius. Small electrical current stimulation is utilized at night, with electrodes taped to the forehead [872].

Vagus, trigeminal and auditory nerve stimulation have limited clinical evidence supporting its use at present. Transcutaneous cervical (neck) vagal nerve stimulation (tcVNS) has been reported to alter brain activation during trauma scripts, in areas indicating a reversal of changes seen in PTSD, consistent with improved autonomic control. Brain activations were increased in the bilateral frontal and temporal lobes, left hippocampus, posterior cingulate, anterior cingulate (dorsal and pregenual), and right postcentral gyrus. Greater deactivations were observed in the bilateral frontal and parietal lobes and left thalamus, consistent with improved autonomic control [893]. It has been hypothesized that tcVNS decreases neural reactivity to an emotional stressor in limbic and other brain areas involved in stress, with changes over repeated exposures suggesting a shift to cognitive processing of the emotional event [891]. Two small, positive open studies, with a total of 17 patients, exist supporting the use of TNS for PTSD, combined with pharmacotherapy [894, 895]. Closed-loop acoustic stimulation neurotechnology has also been developed, with a published pilot study reporting improvements in depression, anxiety, and posttraumatic symptoms, HRV, baroreflex sensitivity, and indices of inflammation in a military sample [680].

#### 5.7.5 Neurofeedback

Neurofeedback is a non-invasive treatment that may utilize real-time functional neuroimaging technology, such as electroencephalography (EEG) or functional magnetic resonance imaging (fMRI), to train the brain to self-regulate through a form of biofeedback [896]. Electrodes are placed on a person’s head and connected to a computer monitor with EEG software installed. A protocol is selected, based on specific amplitudes and/or frequencies of an individual’s electrophysiological activity. The EEG sends information to neurofeedback software, which provides individual feedback, in the form of audio or visual stimuli. This could be a video game, for example, that the person controls as the person changes brain rhythms to impact the display on the screen. EEG neurofeedback is now clinically feasible in community practice settings and has been evaluated for monotherapy or adjunctive therapy for PTSD.

A review of neurofeedback for PTSD is published elsewhere [897]. While initial studies were small and heterogeneous, results are promising for reduction in PTSD symptoms, anxiety, emotional dysregulation and depersonalization/derealization, with some studies reporting a medium to large effect size for PTSD and emotional dysregulation [897]. More recently, self-administered, mobile neurofeedback has been reported to reduce both PTSD symptoms, comorbid chronic pain, anger, sleep disturbance, depressive symptoms, and suicidal ideation in veterans with PTSD, chronic pain and traumatic brain injury [898], and partly normalize DMN and SN brain connectivity [899]. Another small RCT in children with developmental trauma also showed promise, reporting decreased PTSD symptoms, internalizing, externalizing, and other behavioral symptoms, and significantly improved executive functioning of children with severe histories of abuse and neglect resistant to previous treatment [900]. However, while promising, neurofeedback is early in its development, awaiting larger, more robust clinical trials to determine its place in the armamentarium.

#### 5.7.6 Stellate Ganglion Blockade (SGB)

Ultrasound-guided stellate ganglion block (SGB) is an injection of local anesthetic into the neck to temporarily block the cervical sympathetic trunk, which controls the body's fight-or-flight response [901]. Typically, it is a 30-minute outpatient procedure, which has been reported to result in immediate relief. Both right and left-sided SGB have been studied [902]. The first military, multisite, sham-controlled RCT of two right stellate ganglion block procedures for PTSD, given two weeks apart, showed reduction in PTSD symptoms over 8 weeks [901]. The therapeutic benefits of SGB can be sustained up to 3 to 6 months, despite the procedure’s use of a temporary local anesthetic. It may be hypothesized that SGB’s peripheral effects may work through the vagus nerve by modulating fear-related brain regions responsible for homeostatic autonomic control and fear memory [903].

## 6 PHASE ORIENTATION OF TREATMENT DELIVERY

With a growing number of interventions being recommended by guidelines, as well as a vast number of emerging treatments, there is a clear need to determine which intervention would be appropriate for whom and in which phase of treatment. To help answer these questions, a neurobiologically-driven staging model of PTSD has been proposed, which operationalizes the progression of the disorder over time and grades of therapy resistance [19, 904]. Such a model is necessary to guide research efforts and find successful interventions for those in whom current standard therapies have failed [905]. The model has also been proposed as a method to improve clinical decision making by systematically mapping symptom severity indicators, duration of the disorder, effects of comorbid somatic and psychiatric conditions, and symptom patterns. Treatment recommendations can be derived from the model and tested for specific stages of the disorder. In the future, this may lead to a more sophisticated grid to personalize interventions proportionate to someone’s risk of illness progression.

### 6.1 Introduction to a Stage Orientation to PTSD

Somatic illnesses such as cancer and diabetes have benefitted from clinical staging models for a long time in terms of early detection and disease management. The first efforts to translate the principles of staging from somatic to mental disorders date from 1993, when Fava and Kellner introduced staging methods for unipolar depression, bipolar disorder, panic disorder, and schizophrenia [906]. Descriptions of staging principles in the field of PTSD are even more recent [19, 905]. Based on a large body of research regarding symptom trajectories and associated shifts in neurobiology becoming available in the past years, the staging model for PTSD was recently updated and revised [904].

Longitudinal symptom trajectories have provided an important foundation to define prognostic and therapeutic differences between individuals in staging approaches. Although DSM-5 diagnostic criteria for PTSD specify an acute, chronic, and delayed onset form of the disorder [13], symptom patterns are assumed to be the same for each PTSD trajectory. Emerging evidence from network analyses, however, shows that the internal structure and linkage of symptoms changes as the disorder progresses [907-909]. These studies have pointed to the fact that it is important to consider changes in the phenomenology of the disorder over time as well as effects of secondary phenomena (*e.g.*, comorbid somatic or mental conditions beyond the primary syndrome). The lack of acknowledgment of illness progression and illness extension is considered an important limitation of traditional taxonomies of mental disorders [910-912].

Other critical concepts in delineating prognostic and therapeutic differences in psychiatric staging models have been kindling and sensitization [245, 913] and allostatic load [914]. The principle of kindling holds that long-term central nervous system challenges could permanently alter neuronal activity [913], whereas sensitization refers to a progressive autonomic hyper-excitability to, for instance, trauma-related events or stimuli [915]. Allostatic load serves as a framework to describe up- and downregulation of different activating and inhibiting systems [252]. This ultimately leads to a cumulative process of ‘wear and tear’ explained by multiple factors including genetic load, life stressors, and secondary phenomena. Central in the current staging model is the finding that inflammatory and neuro-hormonal responses in the aftermath of trauma change with time, and can be adaptive or maladaptive depending on the stage they occur in. An example is a high level of inflammatory mediators in the direct aftermath of trauma which appears to have a protective effect [916], while at a later stage, inflammation predicts symptom exacerbation [917]. Processes of kindling, sensitisation, and allostatic load are assumed to underlie a range of phenomena that have been shown to be time-dependent in PTSD: the progressive loss of neural tissue, stimulus generalization, cognitive dysfunctions, and accelerated aging.

### 6.2 International Consensus

To tie in with staging models for other mental disorders [918] and an international consensus statement on staging [912], the current approach for PTSD consists of a four-stage model to categorize illness progression. The progression axis ranges from stage 0: *trauma-exposed asymptomatic but at risk* to stage 4: *severe unremitting illness of increasing chronicity*. To further specify phenomena of illness extension in PTSD, axes were elaborated based on the Research Domain Criteria (RDoC) of the National Institute for Mental Health [919-921], as well as an additional dimension of stress and emotion regulation and maintenance of consciousness [922]. Extension axes were termed neurobiological markers, information processing systems, psychophysiological stress reactivity, and consciousness dimensions.

Table **6** shows the proposed staging model with the operationalization of progression and extension processes for PTSD. The staging model assumes a progressive course of the disorder. Unlike the staging models in chronic somatic diseases, it does not imply that all individuals move toward the end stage and allows for recovery from all stages except the final one. Manifesting symptoms that belong to a certain stage, however, does carry a considerable risk to progress to the next stage.

### 6.3 Tailoring Treatment Approaches

A staging model for PTSD has the potential to assist in answering questions such as at what stage of the disorder and with which phenotype is a treatment likely to be more or less effective [904]. These are important when considering at which point emerging treatments should be offered for optimal therapeutic benefit. Furthermore, without this approach of staging progression and treatment resistance, potentially useful treatments may fail to show benefit as they have been tested selectively in specific patient groups. Both current guideline-recommended treatments and emerging treatments can be tailored to the stage the disorder is in, to potentially lead to better efficacy of treatment approaches. Table **7** provides a proposal on how these could be matched for several treatment approaches we described earlier.

It is important to recognize that more longitudinal studies are needed to investigate assumptions made in the model and to determine the reliability and validity of the dimensions. The neurobiological underpinnings of the clinical picture still need to be matched to the stages with greater specificity in order to work with this model in clinical practice. With this knowledge, a comprehensive neurobiological profile could be composed based on an individual’s position on each of the axes of the proposed model. Machine learning algorithms can assist in defining these stratified approaches, which have the potential to be more cost-effective than standard stepped care approaches [923].

### 6.4 Revisioning Systems of Care - Personalizing Service Delivery

Despite the development of a range of evidence-based pharmacotherapies and psychotherapies, as this review demonstrates, current practices point to both treatment and research-practice gaps in PTSD. We reviewed state-of-the-art therapies in PTSD that have recently achieved prominence and may significantly impact PTSD treatment, as well as novel interventions in early preclinical and clinical research. However, exciting progress in PTSD treatment will be of no use if not integrated into accessible, high-quality systems of mental health care. Chronic underinvestment in prevention, early intervention and clinical treatment must change to address the current prevalence of physical and mental disability, suffering, and suicide. More money alone will not solve the problem. In line with some of the future mental health developments outlined by Stein *et al.* (2022) [924], we foresee the following for PTSD: more personalized pharmacotherapy; a further scale-up of practice-based psychotherapy; digital therapies; and global mental health and task-sharing approaches. The transitions in emerging practices are in some forms different from current practices and reflect both hype and hope. Service innovation is critical, driven by and informing a strategic, central research plan. Nijdam *et al.* proposed building a model of care around a comprehensive biological profiling of each patient [904].

Excitement related to MDMA, ketamine and psychedelic-assisted psychotherapy also comes with questions about how these therapies will be integrated into clinical practice in an equitable way. Regulatory challenges, therapist training and cost, and vulnerabilities inherent in altered states of consciousness need to be addressed. Exciting neurobiological data has the potential to blur the impact of psychosocial factors, including set and setting, in enacting the benefits, which are important for the successful translational implementation of these interventions. As these medicines can alter neural connectivity and boost neuroplasticity, there has been a tendency to overfocus on medication effects, rather than seeing them as catalysts for good psychotherapy. Some argue that the inner-directed MAPS model, which has become somewhat of a standard, is too expensive and nonspecific, calling for the addition of more specific modalities, or group therapy, to reduce costs and boost efficacy. MAPS, which used pairs of highly qualified psychotherapists, demonstrated good outcomes despite high “placebo” effects in the psychotherapy only comparator. However, despite criticisms of MAPS’ expensive two-therapist model, there are published positive cost-benefit analyses arguing the cost is justifiable [925, 926]. Given cost constraints in public systems and access issues, it has been argued that psychedelics should be reserved for the most severe, chronic, and treatment-resistant cases, but the opposite may be true, as psychological and pharmacological effects may help facilitate flexibility and openness to benefit from other evidence-based TFPs. Early evidence indicates there may be epigenetic and other modifiers impacting response to treatment [829]. Further studies are needed to address these questions. However, this may be a moot point if these treatments are not integrated into systems of care that can capture such data. Such an environment encourages the rise of private entities to fill the gap, raising concerns about fidelity, quality control and siloed care. The same can be said for the technology-driven novel treatments for PTSD. These also require investments, training and integration into a system of care.

A stepped care model may be a contribution to deal with these problems, with the establishment of a network of integrated centres of excellence [19], with central coordination. These centres could provide a suite of treatments, including emerging therapies not readily accessible elsewhere and second opinions for complex cases. These centres would also provide clinical leadership in service development, training and research. Systematic patient assessment and symptom monitoring are crucial [145], and should incorporate risk factors and biomarkers, as they become available. This will require systems for optimal, cost-effective electronic data capture and management, and appropriate structural and administrative support, to build the evidence base for causes, consequences and treatment effectiveness for mental illness, across diagnostic categories. This will allow continuous improvement of care pathways, and access to information needed for individualized treatment planning and development of proper machine learning algorithms to improve future care pathways.

This step-wise approach would also include:

- Access to diagnosticians and multidisciplinary services, providing more comprehensive care and addressing psychosocial needs.
- Integration of care for prevention, early intervention, treatment of comorbidities, and system navigation.
- Care pathways across crisis, inpatient, public, and private settings.
- Incorporating a disease staging model, which has the potential to match treatments to the duration and severity of the psychiatric disorder.
- Emphasis on both access and quality of mental health treatment, which is individualized, person-centred, and matched to the stage of illness, with care coordination for those with complex needs.
- Standardized protocols for assessment, symptom monitoring, follow-up, and decision points to inform clinical care pathways, and documentation of treatment response.
- Referral to secondary and tertiary service networks for those with inadequate treatment response, linking with other parts of the health system, including crisis and inpatient care.
- Incorporating research and quality improvement into clinical care. Having an appropriate, knowledge informed and strategic research program, embedded into clinical care, that includes the investigation of suicidality. Priorities include identifying barriers to access and engagement, psychosocial and neurobiological predictors of improved outcomes, patterns of emergence of psychiatric and medical comorbidity and how these relate to treatment response and suicidality.
- Assessment for and provision of emerging treatments, which can be optimally trialled using common protocols, to determine optimal selection and sequence of interventions.
- Over time, database analysis will allow the prediction of optimally directed treatment, and development of assessment protocols and decision trees for treatment options that are personalized to individuals and their families.
  There may be several advantages to this approach:
- Addressing barriers to care, poor response, and suicide risk.
- Potential system-wide cost savings due to earlier treatment, better treatment algorithms, and improved resource utilization
- Potential for secondary prevention of PTSD, comorbidities, and physical health sequelae of trauma/chronic stress. Reduced duration of untreated illness, which is linked to poor outcomes in other disorders [927].
- Integration of physical, mental and social dimensions, and identification of medical factors impairing psychiatric treatment and vice versa.
- Allowing data linking from multiple data sets and machine learning, creating a positive feedback loop for progress.

## CONCLUSION

This paper provided a comprehensive overview of the current state-of-the-art of PTSD treatment. Following a brief overview of the etiology and epidemiology of PTSD, we have endeavoured to synthesize our current understanding of PTSD, PTSD interventions, and associated challenges. The manuscript demonstrates that the neuroscience of PTSD has matured enormously over the last 4 decades. While a myriad of approaches and evidence-based interventions for PTSD exist, no single magic bullet exists and current clinical delivery is exceedingly insufficient, leaving a significant number of individuals struggling with PTSD and its effects. More is needed to push forward our understanding of PTSD, as well as research and clinical practice. While much knowledge has been gained from fundamental and cross-sectional studies, the field will benefit from longitudinal studies with multilevel phenotyping (from genetic to neuronal networks). Conceptually, just as PTSD needed to be reconsidered a decade ago to include a dissociative subtype, recognition of a moral injury subtype of PTSD may be warranted. From a treatment perspective, early interventions, pharmacological treatments, medication-augmented psychotherapies, psychedelic-assisted psychotherapies, neuromodulation, and technology-assisted interventions each have a contribution to make toward personalized medicine and psychotherapy. Just as technology has been a driver for our conceptualization of PTSD, so will it be integral to the delivery of treatment interventions. The following summarizes salient concepts germane to future progress:

- The conceptualization of PTSD is evolving away from the acute, single trauma, fear neurocircuitry model. There is a need to deepen and incorporate our understanding of PTSD in general, and how horror, disgust, shame, guilt and moral injury impact presentation, chronicity, and treatment outcomes. This also involves conceptualizing PTSD from a transdiagnostic perspective and considering the central role of emotional dysregulation as a core component.
- A shift toward recognition of PTSD as a systemic disorder is imperative. PTSD is a dimensional continuum of posttraumatic illness involving dysregulation of multiple systems (stress, inflammation, hormonal, brain circuitry, *etc.*), with epigenetic vulnerability and subsyndromal symptoms that start before PTSD criteria are fully met. This explains the complex, heterogeneous nature of the illness, extensive comorbidity, and poor response to treatments, which are often geared to the acute phase.
- Pharmacological and nonpharmacological interventions can take into account the stage of illness, subtypes and individual factors, such as sex and epigenetic differences. Challenges include increasing engagement, facilitating early identification, understanding predictors of treatment outcome, and developing effective innovations. This will require integration of research into systems of care delivery. RDoC’s dimensional approach in PTSD supports the staging model, with a progression from subsyndromal to chronic and severe forms of PTSD [928].
- Technology offers opportunities to advance assessment, data capture, treatment individualization, service delivery, and research of PTSD. This includes secure data capture technologies, remote delivery, and machine learning.
- PTSD is currently in a “crisis” regarding stagnation of new compounds, and ‘old’ compounds getting a second chance, such as MDMA, psilocybin and LSD. This may lead to novel delivery of treatment, in which the traumatic experience is revisited and utilized for transformative change, potentially boosted by psychoplastogenic changes in neural networks. A new “Golden Hour” is thus created, recognizing that the potential of critical learning periods of the brain in these states can be reinforced by psychotherapy.

It will take the efforts of many, across disciplines and from bench to bedside, to capitalize on this opportunity and rise to the challenges.

## Figures and Tables

**Table 1:** Brain networks involved in PTSD.

-	**Default Mode Network (DMN)**	**Central Executive Network (CEN)**	**Salience Network (SN)**
Core structures	mPFC, PCC, precuneus^1^, medial temporal lobe (including hippocampus)	dlPFC, precuneus, anterior inferior parietal lobule, and part of premotor cortex	dACC, amygdala, insula
Core functions	Self-referential and emotional processing, recollection of prior experiences^2^, default or habitual responses, mentalization and abstract mental state processing^3^	Inhibitory control, emotion regulation, attention regulation	Attention regulation, determining importance of stimuli, regulating other networks
Associated PTSD symptoms	Intrusive symptoms (diminished ability to maintain a calm inner state), altered sense of self or reality (dissociation), avoidance (generalization of fear)	Cognitive deficits, loss of SN regulation. Reduced working memory and emotional control	Hypersensitive threat detection
Circuit alteration found in PTSD	Decreased activity and intrinsic connectivity	Decreased activity and intrinsic connectivity. PTSD-DS shows increased connectivity within CEN	Increased activity and intrinsic connectivity; Hyperconnectivity between SN and DMN^4^; impaired DMN and CEN modulation by SN

**Note:** (Based on Kamiya *et al.* (2020) [208], Akiki *et al.* (2017) [235] and Raichle (2015) [236]).

^1^[236, 237], ^2^[236, 238], ^3^[239], ^4^[240].

**Abbreviations:** CEN, central executive network; dACC, dorsal anterior cingulate cortex; dlPFC, dorsolateral prefrontal cortex; DMN, default mode network; mPFC, medial prefrontal cortex; PTSD, posttraumatic stress disorder; PTSD-DS, dissociative subtype of PTSD; PCC, posterior cingulate cortex, SN, salience network.

**Table 2:** Identifying allostatic load through biological markers.

**Primary Mediators of Allostatic Load (Immediate Correlation with Adrenal Function)**	**Secondary Mediators**	**Tertiary Mediators (Resulting from a Condition of Allostatic Load)**	**Additional Biomarkers ** **(Additional Role in the Allostatic Load Response)**
Cortisol, dehydroepiandrosterone, epinephrine, norepinephrine	Cholesterol, glycosylated hemoglobin, resting systolic and diastolic blood pressure, body mass index, and waist-hip ratio	Cognitive and physical functioning	Glucose levels, lipid profiles, interleukin-6, heart rate variability
“Allostatic load battery”			

**Note**: (based on Seeman *et al*., 2001 [251]).

**Table 3:** Common pharmacotherapies used in clinical practice for PTSD*.

**Class**	**Proposed Mechanism**	**Examples**
Antidepressants	SSRI	Paroxetine, sertraline, fluoxetine, citalopram, escitalopram, fluvoxamine
	SNRI	Venlafaxine, duloxetine
	TCA	Imipramine, desipramine, amitriptyline, nortriptyline
	NaSSA	Mirtazapine
	SARI	Trazodone
	MAOI	Phenelzine, moclobemide
	NDRI	Bupropion
Anxiolytic	5-HT_1a_-agonist	Buspirone
Antiadrenergics	α_1_-antagonist	Prazosin, doxazosin, alfuzosin
	α_2_ agonist	Guanfacine, clonidine
	Beta-antagonist	Propranolol
Antipsychotics	Serotonin dopamine antagonists	Quetiapine, risperidone, olanzapine, aripiprazole, ziprasidone
Anticonvulsants	Mechanisms related to GABA and glutamate, possibly impacting limbic kindling	Valproate, carbamazepine, phenytoin, phenobarbital, lamotrigine, topiramate, tiagabine
Benzodiazepines	Allosteric GABA receptor modulator	Alprazolam, chlordiazepam, chlorazepate, diazepam, lorazepam, oxazepam, temazepam, triazolam
Non-benzodiazepine Hypnotics	Bind to the GABA_A _receptor	Eszopiclone, zopiclone, zaleplon, zolpidem
Hypnotic	Melatonin receptor agonist	Ramelteon

**Abbreviations**: GABA, gamma-aminobutyric acid; MAOI, Monoamine oxidase inhibitor; NaSSA, Noradrenergic, and specific serotonergic antidepressant; NDRI, Norepinephrine and dopamine reuptake inhibitor; SARI, Serotonin antagonist and reuptake inhibitor; SNRI, Serotonin Norepinephrine Reuptake Inhibitor; SSRI, Selective Serotonin Reuptake Inhibitor; TCA, Tricyclic Antidepressant; 5-HT_1a_, serotonin 1A receptor.

**Note**: *[267, 268].

**Table 4:** Common psychotherapies recommended in clinical guidelines for PTSD*.

**Psychotherapy**	**Description**
**Recommended Trauma-Focused** ** Psychotherapies**	
Prolonged Exposure (PE)	A manualized therapy, including trauma psychoeducation, breathing training, *in vivo* exposure, imaginal exposure (repeatedly recounting traumatic memories during sessions and listening to audio recordings of these recollections), and discussion of thoughts and feelings related to those exercises. Uses principles of extinction learning, habituation, and desensitization to ultimately challenge catastrophic expectations.
Cognitive Processing Therapy (CPT)	A manualized adaptation of CBT that focuses on discussion and cognitive reprocessing of key posttraumatic cognitive themes, such as safety, trust, power, control, self-esteem, and intimacy. Also addresses shame, guilt, and mistrust.
Trauma-focused Cognitive Behavioural Therapy (TF-CBT) and Cognitive Therapy (CT) for PTSD	Includes CBT principles combined with trauma processing, which usually involves imaginal or graded *in vivo* exposure. In some studies, this term includes trauma-focused cognitive therapy, which focuses on addressing excessively negative appraisals of trauma, its consequences, or one’s own responses to the trauma.
Eye Movement Desensitization and Reprocessing (EMDR)	Includes exposure to memories while applying a dual attention task, such as alternating eye movements or bilateral body tapping. Dual attention tasks are thought to tax working memory and thereby reduce vividness and emotionality of the memory. This facilitates processing and reconsolidation of new information into the memory. EMDR includes a desensitization phase, cognitive restructuring phase, and a phase that focuses on reducing bodily sensations associated with traumatic memories.
Narrative Exposure Therapy (NET)	Based on modifications to PE and TF-CBT, NET focuses on a person’s life narrative, including that related to the trauma and to positive events, improving coherence and contextualization of the traumatic experiences within a person's whole life and overall identity. Also includes elements of trauma exposure, including experiencing cognitive, emotional, and sensory elements of trauma responses in the present moment.
Brief Eclectic Psychotherapy (BEP)	A manualized approach combining elements of CBT, imaginal exposure, psychodynamic psychotherapy, and grief therapy. It emphasizes expression of trauma-related grief, as well as addressing anger, shame, and guilt, including making meaning of the traumatic experiences. It also includes a ritual of closure (*e.g*., writing a virtual letter to the perpetrator in the case of sexual trauma and burning it at the end of treatment).
**Recommended Non-Trauma-Focused Psychotherapies**	
Interpersonal Psychotherapy (IPT)	Time-limited treatment, developed for Major Depression and adapted for PTSD, focusing on relational aspects contributing to illness, such as complicated bereavement following a death, role dispute (conflict with an important person in the patient’s life), role transition (major life changes), and interpersonal deficits.
Present Centred Therapy (PCT)	Time-limited treatment focuses on increasing adaptive responses to current life stressors and difficulties that are directly or indirectly related to trauma symptoms. Includes common nonspecific psychotherapy techniques, such as psychoeducation, facilitating safety and hope, validation and support, expression of feelings, and problem-solving. Diaries are used to record problems of concern throughout the week.
Stress Inoculation Training (SIT)	Based on CBT, SIT involves helping people identify and track their stress and learn coping skills to better manage symptoms, such as deep muscle relaxation, cognitive restructuring, breathing exercises, assertiveness skills, thought stopping, and role play.

**Note**: *[254, 255, 280-282, 291].

**Table 5:** Novel interventions for PTSD and proposed mechanisms.

**Intervention**	**Description and Proposed Mechanism in PTSD**
**PTSD Prophylaxis (“Golden Hours”)**	
Propranolol	Beta-adrenergic antagonist; blocks NE-driven fear memory reconsolidation.
Morphine	Opioid agonist analgesic; inhibits NE through the locus coeruleus; impairs fear memory conditioning.
Hydrocortisone	Corticosteroid; negative feedback inhibition of adrenergic stress response; reduces trauma memory formation and facilitates fear extinction.
Oxytocin	May increase or decrease the formation and consolidation of trauma-associated memory.
Nitrous oxide (NO)	Analgesic and NMDAR antagonist; may impair memory consolidation.
Cognitive vaccine	Alters dysfunctional appraisals using cognitive bias modification.
**Psychopharmacologic Interventions for PTSD**	
**Modulators of the Endocannabinoid System**	
CB1 receptor agonists (Cannabis, cannabinoids like THC, CBD, nabilone)	The CB1 receptor is involved in regulation of mood, pain perception, appetite, learning, memory, and inflammation. THC reduces amygdala reactivity and increases mPFC activation and mPFC-amygdala functional coupling during threat. May reduce sleep disturbances and memory reconsolidation.
**Neuropeptides, Hormones, and Related Compounds**	
Hydrocortisone, mifepristone	Hydrocortisone causes HPA axis negative feedback inhibition through GRs. Mifepristone is a selective antagonist of the GR.
CRF type 1 receptor antagonists	Blocks CRF-mediated stress response and may enhance fear inhibition.
Allopregnanolone (Ganaxolone, AC-5216/XBD 173, etifoxine, and YL-IPA08)	GABAA receptor modulator, with anxiolytic effects. Enhances HPA negative feedback to restore homeostasis. May reduce amygdala and insula activity, NE and glucocorticoid signalling, and increase mPFC activity.
Oxytocin	Facilitates trust and social interactions, and improves working memory and executive function. May increase or decrease formation and consolidation of trauma-associated memory. Increased connectivity within dlPFC, and between dlPFC and ACC.
**Glutamatergic Modulators**	
NMDA receptor antagonists (ketamine, ifenprodil, lanicemine, xenon)	Blocks NMDA receptor-mediated formation of intrusive memories. Ketamine also has rapid-onset antidepressant and anti-suicidal action, and upregulates BDNF and dendritic spine growth. See below for psychotherapy-enhancing effects.
Glutamatergic modulation (riluzole, zonisamide, phenytoin, tianeptine)	Riluzole reduces glutamate excitotoxicity by increasing neuronal and glial uptake, decreasing release, and inhibiting voltage-gated sodium channels. Zonisamide indirectly reduces glutamate neurotransmission.
D-serine modulators	Potential for facilitating fear extinction by increasing D-serine mediated NMDA receptor transmission.
**Anti-inflammatory, Analgesic, and Related Compounds**	
Opioids (buprenorphine, morphine, nalmefene) and nitrous oxide	Impairs fear memory conditioning. Opioids inhibit noradrenaline through the locus coeruleus; Nitrous oxide is also a NMDA antagonist.
Opioid antagonists (nalmefene, naltrexone)	Block opioid receptors, which may be involved in dissociative symptoms.
Neuropeptide Y	A neurotransmitter regulator impacting pain, circadian rhythms, learning, memory, neurogenesis, neuroprotection, and neuropsychiatric conditions such as depression, anxiety, and addiction. Has anxiolytic effects and counteracts the action of CRF.
N-acetyl cysteine	Antioxidant, anti-inflammatory, and glutamate modulator.
Other anti-inflammatory compounds	Possible role in neuroprotection; minocycline may reduce brain cytokines. Other candidates include ASA, NSAIDs, and doxycycline.
**Other Pharmacological Interventions**	
Memantine	NMDA antagonist investigated for cognitive impairment in PTSD.
Asenapine, brexpiprazole	Antipsychotics with 5-HT2A and alpha-adrenergic antagonist properties.
Orexin receptor antagonists (suvorexant)	May reduce arousal, promote sleep, enhance consolidation of extinction memories, and improve habituation through orexin system antagonism.
Nepicastat	Inhibits dopamine-β-hydroxylase, blocking conversion of dopamine to noradrenaline, thereby reducing catecholamine levels. May disrupt formation and consolidation of traumatic memories.
**Optimization of Current Evidence-Based Psychotherapies**	
Improving efficiency, access and drop-out rates	Shortening treatment by eliminating unnecessary components or improving effectiveness of components. Group therapy formats, or lay therapists, to improve cost-effectiveness. Incorporating self-help or digital components. Remote (telehealth, videoconferencing) delivery, and in-home treatment, to reduce barriers to treatment.
Altering length of exposure element	Conventional wisdom is that lengthy trauma memory exposures are necessary (*e.g*., PE). However, newer interventions with micro exposures (*e.g*., Flash Technique), brief exposures (*e.g*., modified exposure), pendulating exposure (*e.g*., Somatic Experiencing), or fluctuating exposure (*e.g*., 3MDR) have been developed.
Combining TFP components	Combining cognitive strategies to PE, for example, or EMDR with PE.
EMDR 2.0	Includes three core elements: a) motivating the patient to focus on the distressing memory, b) optimizing activation of the memory network and the body, and c) use of multiple and often multi-modality working memory-taxing tasks. May incorporate techniques such as modifying posture, adding music, movement and imaginal interweaves (similar to rescripting), and techniques to titrate exposure.
Adding non-trauma-focused elements	Addition of coping (PE-Stress Inoculation Training), emotion regulation skills (*e.g*., DBT-PE, STAIR-PE), or SUD treatment (Creating Change, COPE).
Intensive scheduling	“Intensive” or “massed” treatment involving multiple sessions per week, in order to accelerate recovery and reduce dropout.
**Emerging Psychotherapies and Behavioral Treatments**	
**Emerging Trauma-Focused Psychotherapies**	
Accelerated Resolution Therapy (ART)	Manualized therapy combines features of EMDR with imaginal rescripting of traumatic events, visual imagery, use of metaphors, and Gestalt techniques.
Imaginal Rehearsal Therapy (IRT)	CBT and exposure-based intervention for trauma-related nightmares. The person is asked to recall and then rescript nightmares, including more adaptive interpretations, active responses, and positive or acceptable endings. This is rehearsed to displace unwanted content.
Reconsolidation of Traumatic Memories (RTM)	Traumatic memories are reviewed in an imaginary movie theater as a rapid black-and-white movie. The patient modifies key aspects of the target memory (*e.g*., color, clarity, speed, distance, perspective) to make it less impactful.
Dialogical Exposure Therapy (DET)	Combines exposure with gestalt theory, with a focus on “self-processes” distorted by trauma. Major goals include self-acceptance, restoring a sense of self-continuity, and regaining the ability to shape interactions in the environment. Four phases: safety, stabilization, confrontation, and integration.
Somatic, body-oriented psychotherapies	Includes Somatic Experiencing and Sensorimotor Psychotherapy; focuses on interoceptive awareness and titrated experiencing of bodily states, including sensation, posture, urges, and defensive motor patterns. Uses mindful attention to regulate bodily arousal.
Emotional Freedom Technique	Combines somatic and cognitive therapy, combining cognitions with bodily tapping of various acupressure points.
**Non-Trauma-Focused Psychotherapies (Non-exposure based)**	
Adapting psychotherapies developed for other disorders	Adapting therapies for other disorders to PTSD. For example, ACT, Metacognitive Therapy, Interpersonal Psychotherapy, DBT, and Behavioral Activation (see below).
Acceptance and Commitment Therapy (ACT)	Cognitive therapy emphasizing psychological flexibility through mindfulness and acceptance strategies, and committed, value-based action to change behavior.
Metacognitive Therapy	Cognitive-based therapy that emphasizes modifying metacognitive beliefs that perpetuate rumination, worry, hypervigilance, and subsequent maladaptive behaviors. Focuses on the person’s reaction to PTSD symptoms, rather than details of the trauma.
Behavioral Activation	A CBT strategy to improve mood through activity scheduling and reinforcement strategies, understanding impacts of behaviors on thoughts and emotions, and developing positive coping responses.
Creative art therapies	An eclectic mix of therapies, which may include artistic expression, dance, music, theatre, and expressive writing, to facilitate psychological and emotional exploration and experiencing.
Skills based treatments	Seeking Safety, TARGET, and the Unified Protocol all incorporate emotion regulation skills training, often with elements of mindfulness and interpersonal components.
Animal-assisted therapy (canine or equine therapy)	Use of dogs or horses to increase social and community engagement, sense of safety, and attentional control.
Moral Injury (MI) Interventions	Interventions used for MI include PE, CPT, Adaptive Disclosure Therapy, ACT, the Impact of Killing in War, and Trauma-Informed Guilt Reduction Therapy. These often include elements of disclosure, empathy, choice, taking responsibility, forgiveness, making amends, and reconnecting with self and others. Emerging treatments for MI include psychedelic-assisted therapy, virtual reality supported psychotherapy (*i.e*., 3MDR), and animal-assisted therapies.
Spiritually oriented interventions	Interventions that integrate spiritual/religious components, such as Spiritual-Integrated CPT, Soul Repair, and Building Spiritual Strength. Chaplains also draw on the following: pastoral counselling, meaning-making activities, forgiveness and relational repair, spiritual/religious coping, and other practices (*e.g*., prayer; meditation; spiritual guidance/direction; narratives, storytelling, spiritual writing, *etc*.).
**Mind-Body Based Interventions**	
HRV Biofeedback	Self-regulation training through modulation of vagus nerve activity, using real-time feedback from measured HRV.
Mindfulness-based treatments, meditation	Non-judgemental, compassionate attention to the present moment, which facilitates emotion regulation and disentanglement from beliefs.
Yoga	May include movement-based and breathing-based interventions to improve interoceptive awareness, emotion regulation, autonomic function, and connection with the body. May improve tolerance of bodily and other experiences.
Acupuncture	Insertion of thin needles at specific bodily locations to modulate autonomic function through the vagus nerve, and, possibly, modulation of periaqueductal gray, amygdala, and DMN.
**Technology Supported Interventions**	
Internet-delivered treatments	Telehealth, videoconferencing, and asynchronous delivery (internet or computer-delivered interventions, hybrid treatments, apps, and bibliotherapy).
Virtual Reality Exposure Therapy (VRET)	Virtual reality and augmented reality interventions counter avoidance by using visual, auditory, and other sensory elements to activate traumatic memory networks and enhance exposure and engagement.
Computerized cognitive interventions	Attention bias modification, attention control training, *etc*., which aims to normalize attention biases towards and away from threat.
3MDR	An exposure-based intervention incorporating treadmill walking within a personalized, multi-modal, immersive virtual reality environment, and dual attention tasks from EMDR.
**Medication Augmented Psychotherapy**	
D-cycloserine (DCS)	NMDA receptor agonist; enhancement of fear extinction during exposure tasks.
Yohimbine	Alpha-2-adrenergic receptor antagonist thought to facilitate fear extinction by increasing noradrenergic activity and arousal in the presence of conditioned stimuli.
Propranolol, hydrocortisone, oxytocin	As per the descriptions above under “PTSD Prophylaxis” section.
**Psychedelic Assisted Psychotherapy**	
MDMA	Increased release of serotonin, catecholamines, oxytocin, cortisol, prolactin and vasopressin; increased cognitive flexibility and ability to access and process painful emotions, improved fear extinction learning, reduced amygdala activation, and increased vmPFC activity. Oxytocin facilitates self-compassion (reduced shame), connection, trust, and empathy. MDMA-induced positive state interrupts the expectation of intolerable negative emotions upon recall of traumatic memory.
Ketamine	NMDA antagonist; in addition to chemical effects highlighted above (rapid antidepressant effects, blockade of intrusive memory formation, upregulation of BDNF), ketamine may disrupt the DMN, altering self-referential processing. Dissociation from usual defenses, bodily senses, and rigid thought patterns allows access and reprocessing of traumatic or unconscious material, and perspective taking. Higher doses can induce mystical or archetypal experiences, like classic psychedelics.
Classical psychedelics (LSD, psilocybin, DMT)	Cognitive, mood and perceptual effects, due to 5-HT2 agonism, impacts on serotonergic, dopamine, and TAAR, and downstream effects on glutamate and BDNF. LSD also increases oxytocin release, associated with increased empathy and connectedness. Network alterations disrupt cortical control, increase functional connectivity between usually unconnected brain areas, and release inhibition over sensory, interoceptive, and other information, leading to alterations in perception of the self and reality, including mystical or life-changing transcendent experiences.
**Neuromodulation and Nerve Blockade**	
DBS	An electrical pulse generator is placed directly into the brain, targeting specific areas.
ECT, LAP-ST	Electrical induction of a seizure, under anaesthesia, which stimulates specific brain areas and is thought to induce neuroplasticity through increased BDNF.
rTMS	A magnetic field is passed through the scalp and skull, at specific locations, which alters underlying cortical and subcortical activity in specific brain networks.
tDCS	Direct current, *via *scalp electrodes, is passed through the skull to specific cortical areas, to inhibit (*e.g*., amygdala) or activate (*e.g*., dlPFC) the brain.
tcVNS, TNS, and acoustic stimulation	Targets autonomic dysregulation through electrical stimulation of the vagus, trigeminal or acoustic nerve.
Neurofeedback	EEG facilitated biofeedback. Individuals learn to self-regulate by changing brain rhythms to impact a video game display on the screen.
Stellate ganglion blockade (SGB)	Ultrasound-guided injection of local anesthetic into the neck to temporarily block the cervical sympathetic trunk, which controls the body's fight-or-flight response.

**Abbreviations:** NE, norepinephrine; NMDAR, NMDA Receptor; CB1, Cannabinoid Receptor type 1; THC, ∆9-tetrahydrocannabinol; CBD, cannabidiol; mPFC, medial prefrontal cortex; HPA, Hypothalamic-pituitary-adrenal; GR, Glucocorticoid Receptor; CRF, corticotropin-releasing factor; GABAA, gamma-aminobutyric acid type A; dlPFC, dorsolateral Prefrontal Cortex; ACC, anterior cingulate cortex; NMDA, N-methyl-D-aspartate; BDNF, Brain Derived Neurotrophic Factor; ASA, Acetylsalicylic acid; NSAID, Non-steroidal anti-inflammatory drug; 5-HT, Serotonin; PE, Prolonged Exposure; 3MDR, Multi-Modal Motion-Assisted Memory Desensitization and Reconsolidation; EMDR, Eye Movement Desensitization and Reprocessing; DBT, Dialectical Behavior Therapy; STAIR-PE, Skills Training for Affective and Interpersonal Regulation combined with PE; SUD, Substance Use Disorder; COPE, Concurrent Treatment of PTSD and Substance Use Disorders Using Prolonged Exposure; CBT, Cognitive Behavioral Therapy; ACT, Acceptance and Commitment Therapy; TARGET; Trauma Affect Regulation: Guide for Education and Therapy; MI, Moral Injury; CPT, Cognitive Processing Therapy; HRV, Heart Rate Variability; MDMA, Methylenedioxy-methylamphetamine; LSD, Lysergic acid diethylamide, DMT, N,N-dimethyltryptamine; TAAR, trace amine-associated receptors; DBS, Deep Brain Stimulation; ECT, Electroconvulsive Therapy; LAP-ST, Low Amplitude Seizure Therapy; rTMS, Repetitive transcranial magnetic stimulation; tDCS, Transcranial Direct Current Stimulation; PFC, Prefrontal Cortex; tcVNS, transcutaneous cervical Vagus Nerve Stimulation; TNS, Trigeminal Nerve Stimulation; EEG, Electroencephalogram.

**Table 6:** Proposed staging model for PTSD*.

**Extension Progression**	**Possible Neurobiological Markers of Stage**	**Information Processing Systems**	**Psychophysiological Stress, Emotional Reactivity, and ** **Consciousness**
**Stage 0** Trauma-exposed asymptomatic but at risk	Downregulation of GR sensitivity, increased amygdala reactivity, 5FKH genotype, changed circadian cycle/melatonin	Transient attention bias to threat; consolidation of traumatic memories ongoing, deficits in extinction learning and habituation, enhanced contextual anxiety	Increased vigilance
**Stage 1a** Undifferentiated symptoms of mild anxiety and distress	Inflammatory cytokine activation, decreasing response inhibition in frontal cognitive systems	Mild attention or memory difficulties	Heightened basic stress level and some disruption of normal sleeping pattern
**Stage 1b** Subsyndromal distress with some behavioral and functional decline	Increased physiological reactivity to trauma-related stimuli, prolonged autonomic arousal on provocation	Recurrent memories of trauma; increased attention bias to threat	Startle response; some reduction in task-oriented attention in the presence of distractors
**Stage 2** First episode of full-threshold symptoms that has different trajectories	Early and potentially reversible neurobiological disinhibition of frontolimbic circuitry.	Impairments in concentration and memory, changes in sleep architecture (more N1, less N3, REM alterations); spectrum from feeling briefly disconnected from reality to losing consciousness, amnesic spells/gaps in memory, poor recall of extinction and over-sensitization	Anxious avoidance; reduced task focus, nervousness, sleeping problems, jumpiness; anhedonia and emotional numbing; loss of interest and emotionality, emotional instability, sometimes with self-injury; spectrum reaching from brief absentmindedness to seizure-like attacks
**Stage 3** Persistent symptoms which may fluctuate with ongoing impairment: a. incomplete remission of first episode b. recurrence or relapse of PTSD & persistent impairments c. multiple relapses or worsening following incomplete remission	Stronger PFC inhibition, decreased anterior cingulate and hippocampal volume, hypertension and metabolic syndrome; stimulus generalization	Similar to stage 2, but more severe or resistant to therapy; decreased cognitive flexibility leads to dysfunctional cognitions and rigidity; overregulation of (frontal) neuronal networks and disruption of default mode network.	Generalized avoidance leads to more isolation and limited task performance; decrease in synchronization in social conversation due to associative thinking, attenuated emotion recognition; loss of feeling connected; erosion of basic trust in oneself, others, and the world; the increasing influence of guilt and shame
**Stage 4** Severe unremitting illness of increasing chronicity	High allostatic load, high levels of inflammation, medical comorbidities, entrenched sensitization of a range of neurobiological systems.	Neurocognitive decay resulting in premature cognitive aging; moderate memory deficits; chronic dysregulated non-regenerative sleep architecture; thinking characterized by psychotic symptoms; persistent overregulation of (frontal) neuronal networks, increasing dysregulation of default mode network; possibly alterations in brain stem nuclei, hypothalamus (in PTSD: abnormal supramarginal gyrus and superior parietal activation)	Permanent limitations in task performance, strong isolation; extreme avoidance; survival mode; guilt/shame as drivers for behavior; loss of (self-) reflective capacity and empathic connection, retreating into logical linear thinking pattern, unstable self-image; persistent affect dysregulation (*e.g*., fear, guilt, shame)

**Abbreviations**: GR; Glucocorticoid Receptor; 5FKH, 5FKH gene; REM, Rapid Eye Movement; PFC, Prefrontal Cortex.

**Note**: *(based on Nijdam *et al.*, 2023 [904]).

**Table 7:** Proposed guideline-based interventions and emerging treatments per PTSD stage*.

**PTSD Stage**	**Current Evidence-based Interventions ** **Recommended in Guidelines**	**Potential Examples of Emerging Treatments**
**Stage 0**: Trauma-exposed asymptomatic but at risk	Watchful waiting (monitoring symptoms over time)	*e.g*., app-based monitoring
**Stage 1a**. Undifferentiated symptoms of mild anxiety and distress	Psychoeducation, support from family and close relatives	*e.g*., a range of emerging interventions: cortisol, “cognitive vaccination”, ACE inhibitor, and attention training (see paragraph 5.2)
**Stage 1b**. Subsyndromal distress with some behavioural and functional decline	Short interventions: *i. *Interaction-based: limited number of PE sessions, writing therapy	*i. *Interaction-based: neuromodulation and neurofeedback and other technology-based interventions (see paragraph 5.4.3.1, 5.7.2-5.7.5) *ii.* non-interaction based interventions, such as (mindful) relaxation (see paragraph 5.4.2.4)
**PTSD Stage**	**Current Evidence-based Interventions ** **Recommended in Guidelines**	**Potential Examples of Emerging Treatments**
**Stage 2**: First episode of full-threshold symptoms that has different trajectories	Relatively straightforward symptom-focused interventions, such as PE, EMDR, TF-CBT, and CPT (see paragraph 3.3)	Range of novel emerging treatments, based on availability and policy
**Stage 3**: Persistent symptoms which may fluctuate with ongoing impairment	-	-
**a.** Incomplete remission of the first episode	*i.* Psychotherapeutic interventions that address multiple aspects of traumatization or sequential traumatization such as Brief Eclectic Psychotherapy for PTSD, Narrative Exposure Therapy (see paragraph 3.3) *ii.* Pharmacotherapeutic options regulating stress reactivity: SSRIs (paroxetine and sertraline; see paragraph 3.2)	*i*. Intensification, changing modality, combination of therapies or modalities (novel psychotherapy plus pharmacotherapy) *ii. *Pharmacotherapeutic options regulating stress reactivity such as SNRIs, stellate ganglion block, prazosin, or mood stabilizers (see paragraphs 3.2 and 5.7.6) *iii.* Targeting emotional dysregulation, for instance with ACT, mindfulness, or medicinal cannabis (see paragraphs 5.3.1 and 5.4.2.3)
**b.** Recurrence or relapse of PTSD and persistent impairments	Psychotherapeutic interventions that address the person in his/her context, such as interpersonal psychotherapy (see paragraph 3.3), schema therapy	-
**c.** Multiple relapses or worsening following incomplete treatment response	Intensified treatment by means of *i.* ‘massed’ interventions such as highly intensive 1- to 3-week trauma-focused treatments (see paragraph 5.4)	ii. *e.g*., interventions with emerging evidence of effect for treatment-resistant populations: 3MDR, MDMA-assisted psychotherapy, and neuromodulation therapies, DBS, rTMS (see paragraphs 5.4.3.4, 5.6 and 5.7)
**Stage 4**: Severe unremitting illness of increasing chronicity	*i.* Physical: effective medical management of comorbidities	*ii.* Specific interventions for social and vocational assistance *iii.* *e.g*., treatment focused on moral injury, in case of a ‘moral injury subtype’ of PTSD (see paragraph 5.4.2.5) *iv.* *e.g*., interventions focused on maintenance and preventing further comorbidity: nonverbal therapy, service dogs, equine therapy, day treatment and stabilization (see paragraph 5.4.2.3-5.4.2.6)

**Note: **(adapted from Nijdam *et al.*, 2023 [904]).

**Abbreviations:** ACE, Angiotensin I Converting Enzyme; PE, prolonged exposure; EMDR, eye movement desensitization and reprocessing; TF-CBT, trauma-focused cognitive behavioral therapy; CPT, cognitive processing therapy; SSRI, selective serotonin reuptake inhibitors; SNRI, serotonin-norepinephrine reuptake inhibitors; ACT, acceptance and commitment therapy; 3MDR, multi-modal motion-assisted memory desensitisation and reconsolidation; MDMA, 3,4-methylenedioxymethamphetamine; DBS, deep brain stimulation; rTMS, repetitive transcranial magnetic stimulation.

## LIST OF ABBREVIATIONS

ACES: Adverse Childhood Experiences Study
AMPAR: α-amino-3-hydroxy-5-methyl-4-isoxazolepropionic Acid Receptors
ARET: Augmented Reality Exposure Therapy
ART: Accelerated Resolution Therapy
BDNF: Brain Derived Neurotrophic Factor
BPD: Borderline Personality Disorder
CB1: Cannabinoid Receptor Type 1
CB2: Cannabinoid Receptor Type 2
CBD: Cannabidiol
CBT: Cognitive Behavioral Therapy
CEN: Central Executive Network
COPE: Concurrent Treatment of PTSD and Substance Use Disorders Using Prolonged Exposure
CPT: Cognitive Processing Therapy
CPTSD: Complex Posttraumatic Stress Disorder
CRF: Corticotropin-releasing Factor
dACC: Dorsal Anterior Cingulate Cortex
DAT: Dopamine Active Transporter
DBS: Deep Brain Stimulation
DBT: Dialectical Behavior Therapy
DCS: D-cycloserine
dlPFC: Dorsolateral Prefrontal Cortex
DMN: Default Mode Network
DMT: N, N-dimethyltryptamine
DSM-5: Diagnostic and Statistical Manual of Mental Disorders, 5^th^ Edition
ECT: Electroconvulsive Therapy
EEG: Electroencephalogram
EMDR: Eye Movement Desensitization and Reprocessing
fMRI: Functional Magnetic Resonance Imaging
GABA: Gamma-aminobutyric Acid
GluN: Glutamate Ionotropic Receptor units (GluN2A, GluN2B, *etc.*)
GR: Glucocorticoid Receptor
HPA: Hypothalamic-pituitary-adrenal
HRV: Heart Rate Variability
HRVB: Heart Rate Variability Biofeedback
5-HT: 5-Hydroxytryptamine (*i.e.*, Serotonin)
ICD-11: International Classification of Diseases, 11^th^ Edition
IFNɣ: Interferon Gamma
IL: Interleukin
IL-1β: Interleukin 1 Beta
IPT: Interpersonal Psychotherapy
IRT: Imaginal Rehearsal Therapy
iTBS: Intermittent Theta Burst Stimulation
LAP-ST: Low Amplitude Seizure Therapy
LSD: Lysergic Acid Diethylamide
MDD: Major Depressive Disorder
MDMA: Methylenedioxy-methylamphetamine
mGluR: Metabotropic Glutamate Receptor
MI: Moral Injury
mPFC: Medial Prefrontal Cortex
MST: Military Sexual Trauma
3MDR: Multi-Modal Motion-Assisted Memory Desensitization and Reconsolidation
NAC: N-acetyl cysteine
NE: Norepinephrine
NET: Narrative Exposure Therapy
NET: Norepinephrine Transporter
NMDA: N-methyl-D-aspartate
NMDAR: N-methyl-D-aspartate Receptor
NPY: Neuropeptide Y
PE: Prolonged Exposure
PFC: Prefrontal Cortex
PMIEs: Potentially Morally Injurious Experiences
PTSD: Posttraumatic Stress Disorder
PTSD-DT: Posttraumatic Stress Disorder, Dissociative Subtype
RCTs: Randomized Controlled Trials
RDoC: Research Domain Criteria Project
RTM: Reconsolidation of Traumatic Memories
rTMS: Repetitive Transcranial Magnetic Stimulation
SERT: Serotonin Reuptake Transporter
SGB: Stellate Ganglion Block
SN: Salience Network
SSRIs: Selective Serotonin Reuptake Inhibitor
STAIR: Skills Training for Affective and Interpersonal Regulation
SUDs: Substance Use Disorders
TAAR: Trace Amine Associated Receptors
TBI: Traumatic Brain Injury
tcVNS: Transcutaneous Vagus Nerve Stimulation
tDCS: Transcranial Direct Current Stimulation
TF-CBT: Trauma-focused Cognitive Behavioural Therapy
TFP: Trauma Focused Psychotherapy
THC: ∆9-tetrahydrocannabinol
TNF-α: Tumour Necrosis Factor alpha
TNS: Trigeminal Nerve Stimulation
TSPO: Translocator Protein
VMAT2: Vesicular Monoamine Transporter 2
vmPFC: Ventromedial Prefrontal Cortex
VR: Virtual Reality
VRET: Virtual Reality Exposure Therapy
